# TaPHT1;9‐4B and its transcriptional regulator TaMYB4‐7D contribute to phosphate uptake and plant growth in bread wheat

**DOI:** 10.1111/nph.17534

**Published:** 2021-07-02

**Authors:** Pengfei Wang, Gezi Li, Guangwei Li, Shasha Yuan, Chenyang Wang, Yingxin Xie, Tiancai Guo, Guozhang Kang, Daowen Wang

**Affiliations:** ^1^ The National Engineering Research Center for Wheat College of Agronomy Henan Agricultural University Longzi Lake Campus Zhengzhou 450046 China; ^2^ The State Key Laboratory of Wheat and Maize Crop Science College of Agronomy Henan Agricultural University Zhengzhou 450002 China

**Keywords:** functional marker, MYB transcription factor, phosphate transporter, phosphorus nutrition, *Triticum aestivum*

## Abstract

- Efficient phosphate (Pi) uptake and utilisation are essential for promoting crop yield. However, the underlying molecular mechanism is still poorly understood in complex crop species such as hexaploid wheat. Here we report that TaPHT1;9‐4B and its transcriptional regulator TaMYB4‐7D function in Pi acquisition, translocation and plant growth in bread wheat.
- TaPHT1;9‐4B, a high‐affinity Pi transporter highly upregulated in roots by Pi deficiency, was identified using quantitative proteomics. Disruption of TaPHT1;9‐4B function by BSMV‐VIGS or CRISPR editing impaired wheat tolerance to Pi deprivation, whereas transgenic expression of TaPHT1;9‐4B in rice improved Pi uptake and plant growth. Using yeast‐one‐hybrid assay, we isolated TaMYB4‐7D, a R2R3 MYB transcription factor that could activate *TaPHT1;9‐4B* expression by binding to its promoter. Silencing *TaMYB4‐7D* decreased *TaPHT1;9‐4B* expression, Pi uptake and plant growth.
- Four promoter haplotypes were identified for *TaPHT1;9‐4B*, with *Hap3* showing significant positive associations with *TaPHT1;9‐4B* transcript level, growth performance and phosphorus (P) content in wheat plants. A functional marker was therefore developed for tagging *Hap3*.
- Collectively, our data shed new light on the molecular mechanism controlling Pi acquisition and utilisation in bread wheat. TaPHT1;9‐4B and TaMYB4‐7D may aid further research towards the development of P efficient crop cultivars.

## Introduction

Phosphorus (P) is one of the essential mineral nutrients required by plants. However, Pi concentration in soil solution is frequently below the critical level (<10 μM) needed by plants (Raghothama, 1999). To improve crop yields, millions of tons of Pi fertilisers are applied annually to agricultural fields worldwide. However, only 10–20% of the applied Pi is effectively absorbed by plants, while the remaining is lost, which not only increases agricultural costs but also causes environmental pollution (eutrophication of surface water) and acidification of soil (Zhang *et al*., 2013). To ameliorate the reliance of crop production on Pi fertilisers, it is essential to develop P efficient cultivars with enhanced Pi absorption and use efficiency (Grierson *et al*., 2011).

Plants have evolved sophisticated mechanisms to adapt to Pi fluctuations in growth environments. Under low‐Pi conditions, various morphological, physiological and molecular changes, such as formation of finer roots and root hairs, secretion of more organic acids, phosphatases and phytases, and expression of Pi‐starvation‐induced (PSI) genes, are augmented (Heuer *et al*., 2017; Ajmera *et al*., 2019). These events have led to increased Pi availability in the rhizosphere and higher rate of Pi uptake by root cells. Central to Pi absorption is the function of the PHT1 family of high‐affinity Pi transporters (Gu *et al*., 2016; Kopriva & Chu, 2018; Wang *et al*., 2018a). Higher plants often express multiple PHT1 proteins, which play pivotal roles in plant adaptation to Pi‐deficiency conditions because they are rapidly upregulated at low‐Pi concentrations and account for nearly all Pi uptake from the soil and translocation within plants (Gu *et al*., 2016).

To date, nine and 13 PHT1s have been found in the model plants *Arabidopsis thaliana* and rice (*Oryza sativa*), respectively, many of which have been characterised in terms of spatial and temporary expression patterns, transport properties, and roles in Pi acquisition and plant growth and development (Gu *et al*., 2016; Młodzińska & Zboińska, 2016). Furthermore, substantial insights have been gained into the molecular mechanism regulating the transcript and protein levels of PHT1s in the two model species (Gu *et al*., 2016; Młodzińska & Zboińska, 2016; Wang *et al*., 2018a; Ajmera *et al*., 2019). Various transcriptional factors (TFs) have been found to regulate the transcription of PHT1s and many other PSI genes. For example, AtPHR1 and OsPHR1 to OsPHR4, which carry a MYB‐coiled‐coil (MYB‐CC) domain in their proteins, are key transcriptional activators of PSI genes in *Arabidopsis* and rice (Rubio *et al*., 2001; Zhou *et al*., 2008; Guo *et al*., 2015; Ruan *et al*., 2017). In addition, several MYB TFs possessing R2R3 or R3 domain have also been shown to contribute to PHT1 transcriptional regulation, such as OsMYB1, OsMYB2P‐1, OsMYB4P and OsMYB5P in rice and CPC and ETC1 in Arabidopsis (Dai *et al*., 2012; Yang *et al*., 2014, 2018; Chen & Schmidt, 2015; Gu *et al*., 2017).

Bread wheat (*Triticum aestivum* L., BBAADD, 2n = 6x = 42), the most widely cultivated staple food crop (Shewry & Hey, 2015), was evolved through two polyploidisation events; the first one involved the einkorn wheat *T*. *urartu* (AA, 2n = 2x = 14) and a species related *Aegilops speltoides*, which gave rise to a tetraploid species *Triticum turgidum* (BBAA); the second one occurred between tetraploid wheat and *A*. *tauschii* (DD, 2n = 2x = 14), resulting in allohexaploid wheat (Feldman & Levy, 2012). Therefore, the bread wheat genome carries three homoeologous subgenomes (A, B and D), with most genes having two to three homoeologs (IWGSC *et al*., 2018). Compared with *A. thaliana* and rice, bread wheat has considerably more *PHT1* genes (Teng *et al*., 2017; Grün *et al*., 2018; Zhang *et al*., 2019). However, very few of them have been functionally analysed in depth (Secco *et al*., 2017). Yeast complementation assay has been used to demonstrate the Pi transport function of several TaPHT1s (Davies *et al*., 2002; Zeng *et al*., 2002; Guo *et al*., 2014; Teng *et al*., 2017). Positive correlations were found between *TaPHT1* transcript levels and P accumulation in some studies (Teng *et al*., 2013, 2017; Aziz *et al*., 2014; Shukla *et al*., 2016; Deng *et al*., 2018; Grün *et al*., 2018; de Souza Campos *et al*., 2019). Consistent with these results, transgenic overexpression of two *TaPHT1* genes have been found to enhance plant growth and Pi acquisition in bread wheat (Liu *et al*., 2013; Guo *et al*., 2014). Under low‐Pi conditions, at least seven *TaPHT1* genes are transcriptionally upregulated (Teng *et al*., 2017; Grün *et al*., 2018). But to date, it is still unknown if their proteins may accumulate in bread wheat roots, and little molecular insight has been gained into their transcriptional control in response to P supply status, although overexpression of two TFs, TaPHR‐A1 and TaNFYA‐B1, have been shown to stimulate the expression of several *TaPHT1* genes in transgenic wheat plants (Wang *et al*., 2013; Qu *et al*., 2015).

Based on the information presented above, the main objectives of this work were to analyse active TaPHT1s in elite commercial bread wheat cultivars and to identify the TF regulating TaPHT1 transcription in wheat. Therefore, we examined Pi‐deficiency responsive proteins (PDRPs) in bread wheat roots using iTRAQ (isobaric tagging for relative and absolute quantification)‐based proteomic analysis. Analysis of PDRPs enabled us to find four different TaPHT1 proteins whose protein levels were significantly upregulated by Pi deficiency. Detailed analysis on one of the four TaPHT1s, that is TaPHT1;9‐4B, led to the identification of TaMYB4‐7D, a R2R3 type of MYB TF that could bind to the promoter region of *TaPHT1;9‐4B* and promote its transcript level. Together, these results suggest that TaPHT1;9‐4B and its transcriptional regulator TaMYB4‐7D contribute positively to Pi uptake and wheat growth. An elite promoter haplotype of *TaPHT1;9‐4B* was therefore mined for improving Pi uptake and use efficiencies in wheat.

## Materials and Methods

### Plant materials and growth conditions and oligonucleotide primers

The plant materials used in this study included 62 bread wheat cultivars, 11 tetraploid wheat accessions, and 28 diploid wheat relatives (*Aegilops bicornis*, *A*. *longissima*, *A*. *sharonensis*, *A*. *speltoides* and *A*. *tauschii*) (Supporting Information Table S1). Interestingly, cv Zhoumai 18, an elite bread wheat variety widely cultivated in China (Zhou *et al*., 2014), was used for proteomic analysis. Seeds were sterilised and germinated as detailed in Methods S1. Two‐wk‐old wheat seedlings with three leaves, at the autotrophic stage and sensitive to abiotic stresses (Li *et al*., 2017), were divided into two groups, one remained in Hoagland solution containing sufficient Pi (1 mM, as control), and the other was transferred to Pi‐deficient Hoagland medium (0 mM Pi, with KH_2_PO_4_ replaced by KCl) (Secco *et al*., 2010). The nutrient solution was replenished every day for both groups. Determination of plant growth parameters, total P concentration and anthocyanin content are described in Methods S1. All oligonucleotide primers in this work are listed in Table S2.

### Proteomic analysis and validation by parallel reaction monitoring (PRM)

Total proteins were extracted from the roots of the wheat seedlings grown on Pi‐sufficient or Pi‐deficient media for 8 d using the trichloroacetic acid/acetone method, and quantified using the Bradford protein assay (Bio‐Rad, Hercules, CA, USA) (Wiśniewski *et al*., 2009). Three biological replicates were executed, with at least six plants per replicate. The iTRAQ‐based proteomics experiment is described in detail in Methods S1. A PRM experiment, conducted to validate the expression changes of 11 PDRPs found in iTRAQ analysis, was described in Methods S1.

### Identification of PDRPs

Peptide data were searched against the protein database of Chinese Spring (CS) (IWGSC *et al*., 2018, RefSeq v.1.0) using Blastp (v.2.6) by applying an identity cut‐off of 100% (McGinnis & Madden, 2004). To increase accuracy, the proteins specified by the A, B or D subgenomes were separately searched, and homoeologous loci encoding PDRPs were identified and illustrated (Fig. S1), followed by analysis of potential expression bias among the homoeologs (Hu *et al*., 2014) (Methods S1).

### Functional analysis of TaPHT1;9‐4B

Function analysis of TaPHT1;9‐4B was conducted using yeast complementation assay, virus induced gene silencing (VIGS), CRISPR/Cas9‐mediated genome editing and transgenic expression experiments. To perform the complementation assay, *TaPHT1;9‐4B* coding sequence was cloned into the yeast expression vector Yp112A1NE and transformed into the Pi absorption defective yeast mutant MB192, as described previously (Bun‐Ya *et al*., 1991; Teng *et al*., 2017). Barley stripe mosaic virus induced VIGS (BSMV‐VIGS) (Tufan *et al*., 2011), as well as pWMBX110‐SpCas9‐mediated genome editing (Ma *et al*., 2015; Liu *et al*., 2020), were used to investigate the effects of disrupting *TaPHT1;9* function on Pi absorption and plant growth (Methods S1). Transgenic expression of *TaPHT1;9‐4B* in *japonica* rice (cv Nipponbare) was carried out to assess the effects of overexpressing TaPHT1;9‐4B on Pi uptake and plant growth as outlined in Methods S1.

### Identification and functional investigation of TaMYB4‐7D

Total RNAs (2 μg) isolated from the roots of wheat seedlings grown in Pi‐deficient medium for 8 d were used to construct a cDNA library for yeast‐one‐hybrid (Y1H) assay to identify potential *trans*‐factors binding to the promoter region of *TaPHT1;9‐4B*, which was amplified from the cv Zhoumai 18. Screening this library led to the finding of the transcription factor TaMYB4‐7D capable of binding to the *TaPHT1;9‐4B* promoter. Details of the screening and related validation experiments are described in Methods S1. The transcriptional activation potency of TaMYB4‐7D was tested in the yeast strain AH109 as described in the *Yeast Handbook* (Clontech, Palo Alto, CA, USA). Functional analysis of TaMYB4‐7D by BSMV‐VIGS is outlined in Methods S1.

### Nucleotide diversity analysis of *TaPHT1;9‐4B* and *TaMYB4‐7D*

Genomic DNA samples, isolated from the 62 bread wheat cultivars (21 landraces and 41 modern cultivars) and 39 wheat relatives (Table S1), were used for nucleotide diversity (π) analysis (Methods S1).

### Chromosomal assignment of genes, subcellular localisation of proteins, qRT‐PCR assay, haplotype analysis and development of CAPS marker

Details of these experiments are provided in Methods S1.

### Statistics

Numerical values were calculated as means ± standard deviation (SD), which were statistically analysed using either Student’s *t*‐test or one‐way analysis of variance (ANOVA) followed by Duncan's multiple range test installed in the spss statistics package (SPSS Inc., Chicago, IL, USA).

## Results

### Identification of PDRPs

After 8 d culture under Pi‐deficiency (0 mM Pi, NP) conditions, wheat seedlings showed inhibited leaf growth but enhanced root length relative to those grown in the presence of 1 mM Pi (Fig. S2a). Quantitative analysis confirmed these phenotypic changes (Fig. S2b–e). Pi‐deficiency treatment decreased P concentrations in root and shoot tissues (Fig. S2f,g). Because decrease in P concentration and change of plant growth were both observed at 8 d after Pi deficiency (Fig. S2), we therefore considered that the 8 d time point was suitable for studying the effects of Pi deficiency on wheat root proteome using the iTRAQ approach.

**Fig. 1:**
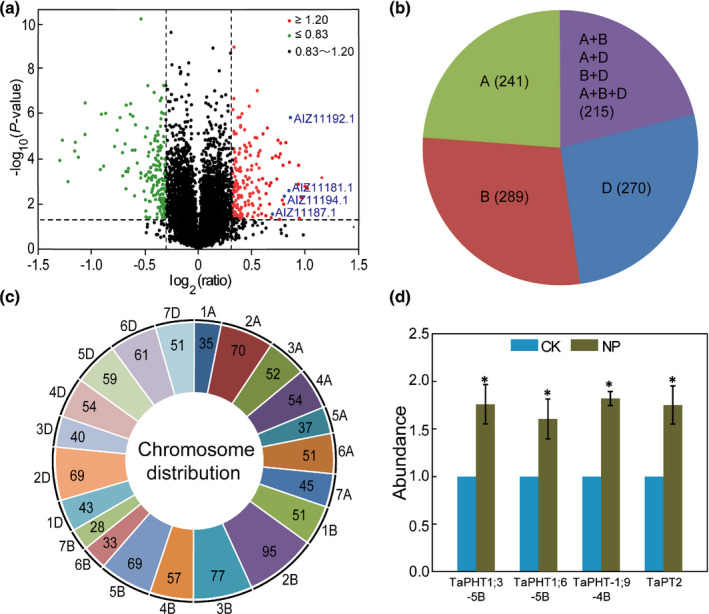
Characterisation of wheat PDRPs. Two‐week‐old wheat seedlings were transferred to Pi‐deficient (0 mM Pi, NP) or ‐sufficient (1 mM Pi, CK) Hoagland media, with their root proteins examined by iTRAQ‐based proteomic analysis at 8 d after transfer. (a) Volcano plot of the identified PDRPs. Blue dots represent the four identified phosphate transporter proteins (AIZ11181.1, TaPHT1;3‐5B; AIZ11187.1, TaPHT1;6‐5B; AIZ11192.1, TaPHT1;9‐4B; AIZ11194.1, TaPT2). (b) Classification of the 1015 PDRPs mapped to specific wheat chromosomal loci, with 241, 289 and 270 PDRPs expressed from single homeologs in the A, B or D subgenomes and the remaining 215 PDRPs specified by two or three homeologs in the three subgenomes. A + B, A + D and B + D: PDRPs specified by two homeologs; A + B + D: PDRPs expressed from all three homeologs. (c) Distribution of homeolog‐specific PDRPs on 21 wheat chromosomes. (d) Relative changes in the abundance of TaPHT1;3‐5B, TaPHT1;6‐5B, TaPHT1;9‐4B and TaPT2 proteins in the roots of the wheat seedlings cultured under Pi‐deficient (NP) or ‐sufficient (CK) conditions. The amount of each protein in Pi‐sufficient roots was set as 1.0 to facilitate the comparison. Data represent means ± standard deviation (SD) of three biological replicates. Asterisks indicate statistically significant differences (*P* < 0.05; Student's *t*‐test).

In total, 20 733 peptides were identified from 151 654 mass spectra, which corresponded to 6842 expressed proteins when searched against the NCBI nonredundant database (Dataset S1). Of them, 4306 were detected in all three biological replicates of both control and Pi‐deficiency treatments (Dataset S1). In total, 323 PDRPs showed comparatively larger changes in their abundance (indicated by unique peptide LC‐MS/MS reporter peak area), being upregulated (≥1.20‐fold) or downregulated (≤0.83‐fold) (Fig. 1a; Dataset S1). The PDRPs were functionally involved in many biological processes (e.g. protein metabolism, signal transduction and transport) (Dataset S1). Eleven PDRPs were selected for validation by a PRM experiment. A high consistency was found between the expression changes revealed using iTRAQ or PRM approaches for nine of the 11 examined proteins (Fig. S3; Dataset S2), therefore confirming the reliability of the iTRAQ dataset generated in this work.

### Expression characteristics of PDRPs

Genomic distribution of PDRPs was studied using the CS genome assembly (Methods S1). Among the 20 733 peptides and 4306 PDRPs, 15 044 peptides and 4013 PDRPs were matched to the predicted proteins of CS (Dataset S3). Furthermore, 1015 PDRPs could be assigned to specific homoeologs in the A, B, or D subgenomes (Dataset S3); the remaining could not be assigned to specific subgenomes. Markedly, of the 1015 PDRPs, 738 (72.7%) were strongly affected by homoeolog expression bias, with each PDRP specified by only one of the two, three or more homoeologs; 62 (6.1%) were expressed from the single genes (located in A, B or D) without homoeologs in the other two subgenomes (Dataset S3). Among the single homoeolog‐derived PDRPs (738 + 62), 241, 289 and 270 belonged to the A, B and D subgenomes, respectively (Fig. 1b; Dataset S3). The remaining 215 PDRPs, each specified by two or three homoeologs (Fig. 1b; Dataset S3), were not affected by homoeolog expression bias, with 65 of them showing unbalanced expression in one or two homoeologs (*P* < 0.05) (Fig. 1b; Dataset S3). Clearly, single homoeolog‐derived proteins dominated the 1015 PDRPs. Additional analysis showed that the 1131 PDRPs were distributed mainly on chromosomes 2B, 3B, 2A, 5B, 2D, 6D, 5D and 4B (Fig. 1c).

### Primary structure, subcellular location and yeast complementation assay of TaPHT1;9‐4B

Among the identified PDRPs, four high‐affinity PHT1 proteins (TaPHT1;3‐5B, TaPHT1;6‐5B, TaPHT1;9‐4B and TaPT2), each being specified by a single homoeolog, were significantly upregulated by Pi deficiency, with the average fold of induction of TaPHT1;9‐4B being relatively high (Fig. 1d). The high induction of TaPHT1;9‐4B by Pi deficiency was also found in the PRM analysis (Fig. S3; Dataset S2). In the study by Teng *et al*. (2017), the transcripts of TaPHT1;3 and TaPHT1;6, named as TaPHT1.3 and TaPHT1.6, respectively, were detected in both wheat roots and shoots, whereas those of TaPHT1;9 (TaPHT1.9) were found predominantly in the roots by qRT‐PCR. Amino acid sequence comparisons indicated that TaPHT1;3‐5B, TaPHT1;6‐5B and TaPHT1;9‐4B were identical to TaPht1;4‐Chr5BL, TaPht1;6‐Chr5BL and TaPht1;1a‐Chr4BL, respectively, in the wheat PHT1 nomenclature system proposed by Grün *et al*. (2018). Searching the genomic sequence of CS revealed that *TaPHT1;3‐5B*, *TaPHT1;6‐5B* and *TaPHT1;9‐4B* corresponded to the chromosome loci *TraesCS5B02G512000*, *TraesCS5B02G470100* and *TraesCS4B02G317000*, respectively. TaPT2, renamed as TaPHT1.10‐U by Teng *et al*. (2017) or TaPht1;2a‐Chr4DL by Grün *et al*. (2018), corresponded to *TraesCS4D02G313800* in the CS genome sequence; it has been shown to function as a high‐affinity Pi transporter using yeast complementation assay and by analysing overexpression and RNAi transgenic wheat lines (Davies *et al*., 2002; Zeng *et al*., 2002; Guo *et al*., 2014). Because TaPHT1;9‐4B was found expressed in wheat roots by both previous study (Teng *et al*., 2017) and our work (Figs 1d, S3), and yet its role in Pi transport and utilisation had not been well studied, we therefore focused on analysing the function of TaPHT1;9‐4B in subsequent research.

**Fig. 2:**
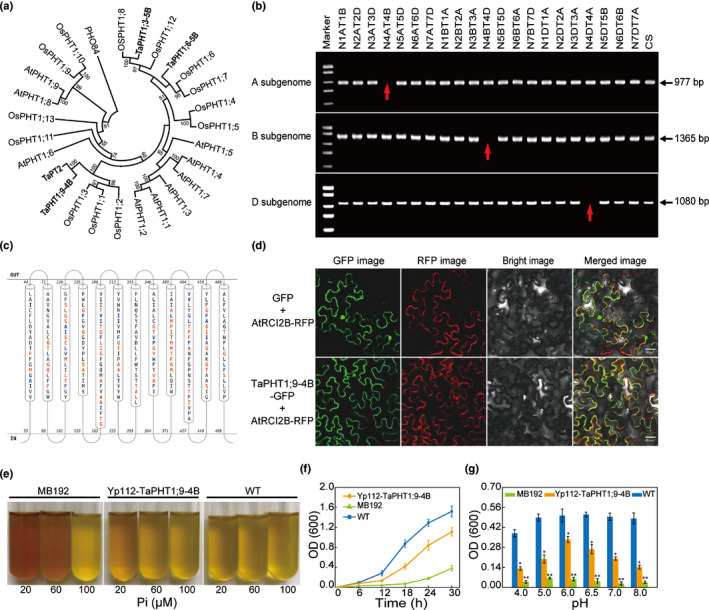
Characterisation of TaPHT1;9‐4B. (a) Phylogenetic tree of representative plant PHT1 proteins (Supporting Information Table S3), which was constructed using the neighbour‐joining software with bootstrap values calculated using 1000 permutations. The four TaPHT1 proteins identified in this work are written in bold. (b) Chromosomal location of three *TaPHT1;9* homeologs. PCR mapping was performed using the genomic DNA samples of Chinese Spring (CS) and derivative nulli‐tetrasomic (NT) lines. No amplicons were obtained for the NT lines N4AT4B, N4BT4D and N4DT4A (arrowed), indicating that *TaPHT1;9* homeologs were located on chromosomes 4A, 4B and 4D, respectively. (c) Structure of the predicted transmembrane domains of TaPHT1;9‐4B using the software rhythm (http://proteinformatics.charite.de/rhythm). In total, 12 transmembrane domains with both N‐ and C‐termini facing the cytosolic side were predicted. (d) Subcellular localisation of TaPHT1;9‐4B. The green fluorescence of TaPHT1;9‐4B‐GFP fusion protein was localised to the plasma membrane, whereas that of GFP was distributed throughout the cell. An AtRCI2B‐RFP fusion protein was used as a positive plasma membrane marker. Bars, 20 μm. (e–g) Functional complementation assay of TaPHT1;9‐4B using the yeast strain MB192 with mutated high‐affinity Pi transporter PHO84. Expression of TaPHT1;9‐4B restored MB192 growth under low‐Pi conditions (20 and 60 μM) as indicated by colour change of growth medium (e). Measurement of cell growth revealed partial complementation of PHO84 by TaPHT1;9‐4B (f), with the highest degree of complementation achieved at pH 6.0 (g). Data represent means ± SD of three biological replicates. Asterisks indicate statistically significant differences (*, *P* < 0.05; **, *P* < 0.01; Student’s *t*‐test).

Phylogenetic analysis with previously characterised *Arabidopsis* and rice PHTs, as listed in Table S3, showed that TaPHT1;9‐4B and TaPT2 were tightly clustered with the three rice PHT1 proteins OsPHT1;1, OsPHT1;2 and OsPHT1;3 (Ai *et al*., 2009; Sun *et al*., 2012; Chang *et al*., 2019) (Fig. 2a). TaPHT1;3‐5B was closely related to OsPHT1;8 (Jia *et al*., 2011), while TaPHT1;6‐5B was aggregated with OsPHT1;6 and OsPHT1;7 (Ai *et al*., 2009) (Fig. 2a). TaPHT1;9‐4B and TaPT2 were 99.04% identical, and they exhibited 78.69–80.04% identities to OsPHT1;1, OsPHT1;2 and OsPHT1;3 (Table S4). TaPHT1;3‐5B was 87.59% identical to OsPHT1;8, while the identities of TaPHT1;6‐5B to OsPHT1;6 and OsPHT1;7 were 77.16% and 75.19%, respectively (Table S4).

PCR amplification of *TaPHT1;9* homoeologs using subgenome‐specific primer pairs produced three amplicons in wild‐type (WT) CS but only two amplicons in the N4AT4D, N4BT4D and N4DT4B lines that lacked 4A, 4B and 4D chromosomes, respectively (Fig. 2b), confirming that *TaPHT1;9* homoeologs are located on group 4 chromosomes. Consistent with this result, three *TaPHT1;9* homoeologs are present on the 4A, 4B and 4D chromosomes of CS, respectively, with *TaPHT1;9‐4B* physically mapped to the 606.81 Mb position on chromosome 4B.

*TaPHT1;9‐4B* did not have intron in its coding region or the 5′ and 3′ untranslated regions; its coding sequence was 1566 bp, whose deduced protein contained 521 amino acids (Fig. S4). Consistent with the previous finding (Teng *et al*., 2017), we observed that *TaPHT1;9‐4B* was predominantly expressed in wheat root, with its transcript level dramatically upregulated by Pi‐deficiency treatment (Fig. S5). *In silico* analysis predicted that the secondary structure of TaPHT1;9‐4B contained 12 putative transmembrane (TM) domains (Fig. 2c). Expression of *TaPHT1;9‐4B‐GFP* fusion cistron under the control of *CaMV35S* promoter produced GFP fluorescence only at the plasma membrane (PM), suggesting that TaPHT1;9‐4B was targeted to the PM (Fig. 2d), which was marked by the RFP fusion protein of a known hydrophobic and PM‐located protein AtRCI2B (Medina *et al*., 2007).

Expression of *TaPHT1;9‐4B* in the yeast strain MB192, which carries a mutation in the high‐affinity Pi transporter PHO84 (Bun‐Ya *et al*., 1991), restored its growth under low‐Pi conditions (20 and 60 μM) (Fig. 2e). Quantitative growth curves demonstrated that TaPHT1;9‐4B partially complemented the function of PHO84 (Fig. 2f). However, the functional complementation by TaPHT1;9‐4B was affected by pH, with the highest degree of complementation observed at pH 6.0 (Fig. 2g).

### Analysis of *TaPHT1;9‐4B* using BSMV‐VIGS in wheat and ectopic expression in rice

**Fig. 3:**
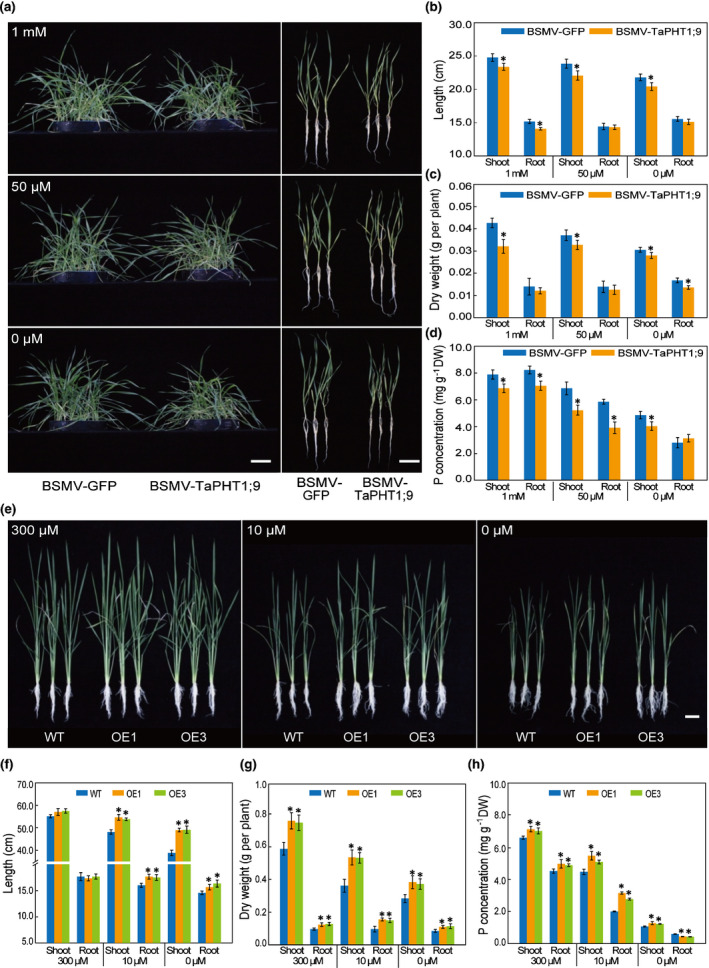
Functional analysis of *TaPHT1;9‐4B* using BSMV‐VIGS in wheat and ectopic expression in rice. (a–d) Phenotype and growth parameters in BSMV‐VIGS experiment. The wheat plants infected by BSMV‐GFP or BSMV‐TaPHT1;9, cultured under Pi‐sufficient (1 mM), low‐Pi (50 μM) or Pi‐deficient (0 μM) Hoagland solutions for 10 d, were examined for overall growth morphology (a) and shoot and root related parameters including lengths (b), dry weights (c) and P concentrations (d). (e–h) Phenotype and growth parameters in transgenic rice experiment. Two *TaPHT1;9‐4B* overexpressing transgenic rice lines (OE1 and OE3), cultured under Pi‐sufficient (300 μM), low‐Pi (10 μM) or Pi‐deficient (0 μM) nutrient solutions for 21 d, were analysed for overall growth performance (e) and shoot and root related parameters including lengths (f), dry weights (g) and P concentrations (h). Bars, 5 cm. Data represent means ± SD of three biological replicates. Asterisks indicate statistically significant differences (*, *P* < 0.05; Student’s *t*‐test).

We analysed the effects of silencing *TaPHT1;9* expression in bread wheat using BSMV‐VIGS. A 256‐bp cDNA fragment conserved among all three *TaPHT1;9* homoeologs was used to construct the recombinant virus BSMV‐TaPHT1;9 for eliciting VIGS (Methods S1; Fig. S6). BSMV‐GFP was used as a control for VIGS. At 8 d after viral inoculation, BSMV‐TaPHT1;9‐infected leaves showed mild chlorosis, and *TaPHT1;9* expression level was decreased by > 62.0% (Fig. S7). Subsequently, BSMV‐TaPHT1;9‐ and BSMV‐GFP‐infected seedlings were separately transferred to Hoagland solution containing different concentrations of Pi (0 μM, 50 μM or 1 mM) for 10 d. As shown in Fig. 3a–c, BSMV‐TaPHT1;9‐infected plants were negatively affected in morphology and growth parameters, including shoot and root length and dry weight, compared with the controls infected by BSMV‐GFP under both Pi‐replete (1 mM Pi) or Pi‐deprived (0 or 50 μM Pi) conditions. P concentrations determined for the shoot and root samples of BSMV‐TaPHT1;9‐infected plants tended to be lower than those measured for the controls, but for the roots treated with 0 μM Pi, P concentration was higher for BSMV‐TaPHT1;9‐infected plants than those infected by BSMV‐GFP (Fig. 3d). The transcript levels of *TaPHT1;3* and *TaPHT1;6* were upregulated in *TaPHT1;9* silenced plants (Fig. S8), implying possible functional interaction between *TaPHT1;9* and the two examined *TaPHT1* genes. We also observed that *TaIPS1*.*1* was upregulated in both *TaPHT1;9* silenced plants and the controls under low‐Pi or Pi‐deficient conditions, with the scale of the upregulation being much larger in the former (Fig. S8). This finding was consistent with the fact that *TaIPS1.1* expression was induced by low‐Pi treatment (Teng *et al*., 2017). The higher induction of *TaIPS1*.*1* in *TaPHT1;9* silenced plants may be associated with a stronger Pi‐deficiency stress in these individuals.

Furthermore, we ectopically expressed *TaPHT1;9‐4B* coding sequence in rice using the ubiquitin gene promoter by *Agrobacterium*‐mediated transformation. Independent T_2_ transgenic rice lines expressing *TaPHT1;9‐4B*, identified by PCR analysis (Fig. S9), were analysed. In general, the two *TaPHT1;9‐4B* overexpression lines (OE1 and OE3) exhibited improved growth parameters than WT control under either Pi‐replete (300 μM Pi) or Pi‐deprived (0 or 10 μM Pi) conditions (Fig. 3e–g). P concentrations in the root and shoot tissues of *TaPHT1;9‐4B* overexpression plants were markedly higher than those of WT controls with the supply of 300 or 10 μM Pi (Fig. 3h). In the absence of external Pi, P concentration increased significantly in the shoots but decreased in the roots of the OE lines (Fig. 3h), suggesting that the overexpressed TaPHT1;9‐4B enhanced Pi translocation from root to shoot tissues under Pi‐deficiency conditions.

### Verification of *TaPHT1;9‐4B* function using CRISPR mutants in wheat

**Fig. 4:**
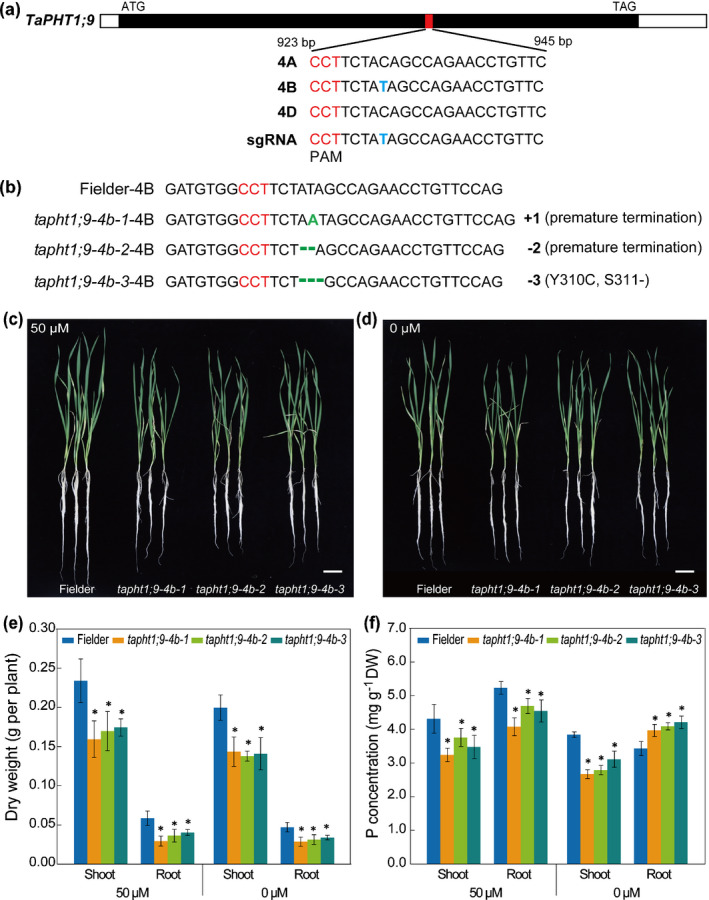
Verification of *TaPHT1;9‐4B* function using CRISPR mutants in wheat. (a) Schematic representation of *TaPHT1;9‐4B* coding region and the single guide RNA (sgRNA) used to generate CRISPR mutants. The protospacer‐adjacent motif (PAM) was highlighted in red. (b) Nucleotide mutations in the three CRISPR mutants (*tapht1;9‐4b‐1*, *‐2* and *‐3*) used for analysing *TaPHT1;9‐4B* function. The green letter and dashed lines represent inserted or deleted nucleotides. A detailed description of the consequences of these mutations on TaPHT1;9‐4B protein was provided in Supporting Information Fig. S10. (c, d) Phenotypes of three CRISPR mutants and WT Fielder control grown under low‐Pi (c), or Pi‐deficient (d) conditions. Two‐wk‐old mutant and WT Fielder plants were transferred to low‐Pi (50 μM) or Pi‐deficient (0 μM) media, with the images shown photographed at 3 wk after the transfer. Bars, 5 cm. (e, f) Shoot and root dry weights (e) and P concentrations (f) of CRISPR mutant and WT Fielder plants cultured in low‐Pi (50 μM) or Pi‐deficient (0 μM) media for 3 wk. Data represent means ± SD of three biological replicates. Asterisks indicate statistically significant differences (*P* < 0.05; Student’s *t*‐test).

We developed three CRISPR mutants for *TaPHT1;9‐4B* using a sgRNA targeting the coding region with the wheat cultivar Fielder (Fig. 4a). While the mutations in *tapht1;9‐4b‐1* and *‐2* led to premature termination of protein sequence, removal of three nucleotides in *tapht1;9‐4b‐3* resulted in one amino acid substitution (Y310C) and deletion of the serine residue at position of 311 (Figs 4b, S10). Through DNA sequencing, we verified that the 4A and 4D homoeologs of *TaPHT1;9* were not edited (mutated) in *tapht1;9‐4b‐1*, *‐2* and *‐3*. Subsequently, 2‐wk‐old CRISPR mutant and WT Fielder plants cultured under normal conditions were transferred to Pi‐sufficient (1 mM), low‐Pi (50 μM) or Pi‐deficient (0 μM) media, and examined after a 3‐wk culture. The three mutants were clearly weaker than the control (Figs 4c,d, S11a), and their shoot and root dry weights were significantly smaller than those of the control (Figs 4e, S11b). The shoot and root P concentrations of the three mutants were all significantly lower than those of the control in the presence of 1 mM or 50 μM Pi (Figs 4f, S11c). Under Pi‐deficient conditions, the shoot P concentration was decreased in all three mutants relative to that of control, but the root P concentration behaved in an opposite manner (Fig. 4f), indicating that the mutation of *TaPHT1;9‐4B* might hinder the translocation of Pi from root to shoot under Pi‐deficiency conditions. These results verified the data obtained by analysing *TaPHT1;9* silenced wheat plants (Fig. 3a–d), and confirmed the function of *TaPHT1;9‐4B* in Pi uptake and translocation in wheat.

### Identification and functional analysis of TaMYB4‐7D

A Y1H assay was conducted using a 1513‐bp fragment of *TaPHT1;9‐4B* promoter as a bait to screen the cDNA library derived from Pi‐deficient roots (Fig. S12). Of the 23 cDNA clones showing Y1H interactions, three represented the same gene coding for a R2R3‐type MYB‐like TF (Table S5). The coding sequence of this gene was 705 bp and yielded a protein of 234 amino acids upon conceptual translation (Fig. S13a), which was identical to TaMYB4 (GenBank accession AEG64799.1) previously found to be expressed in wheat stem and root tissues (Ma *et al*., 2011) (Fig. S13b). In CS genomic sequence, the gene (*TraesCS7D02G272400*) encoding TaMYB4 was located on chromosome 7D. This was validated by PCR analysis of CS and associated nulli‐tetrasomic (NT) lines (Fig. S14a). Therefore, it was designated as TaMYB4‐7D to facilitate further functional analysis. The TaMYB4‐7D‐GFP fusion protein was found only in the nucleus when transiently expressed in tobacco leaf cells (Fig. S14b). When TaMYB4‐7D was expressed in yeast cells using the pGBKT vector, designed to test the transactivation activity of TFs (Zhu *et al*., 2015), the transformed cells showed growth on the synthetic defined medium lacking tryptophan and histidine (SD/–Trp/–His), and the yeast colonies turned blue when grown on SD/−Trp/–His medium containing X‐α‐Gal (Fig. S14c). These results indicated that TaMYB4‐7D was likely to be a functional TF with transactivation activity.

**Fig. 5:**
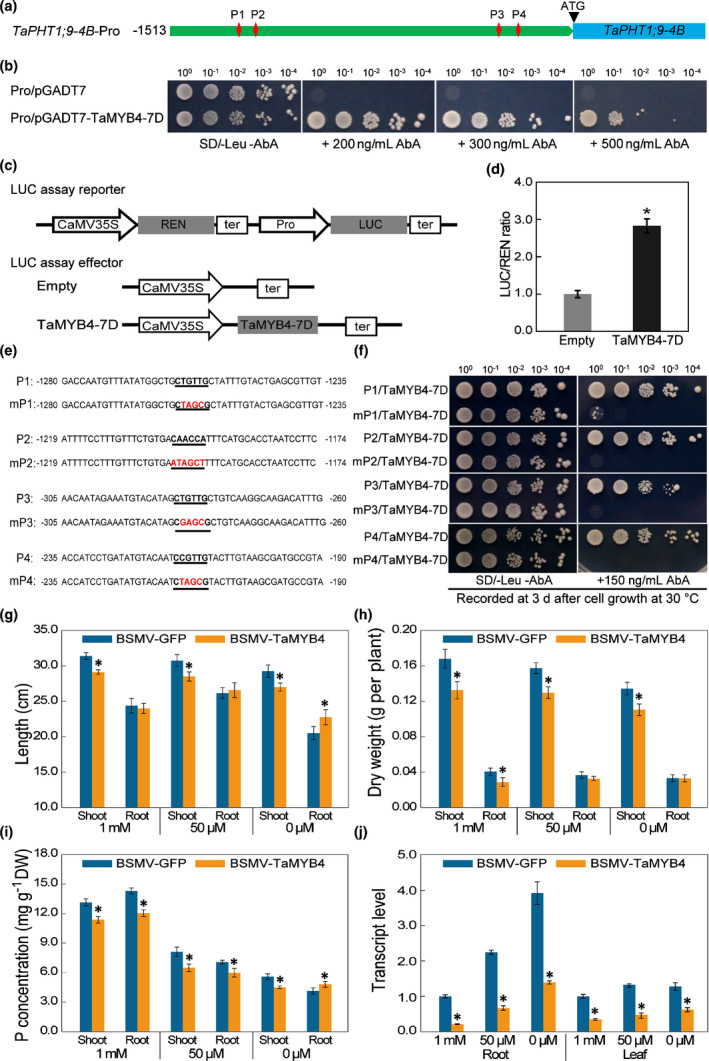
Functional analysis of TaMYB4‐7D. (a) Four predicted MBSs (P1–P4) in *TaPHT1;9‐4B* promoter region. (b) Y1H assay between *TaPHT1;9‐4B* promoter and TaMYB4‐7D, with positive interaction indicated by yeast cell growth in the presence of aureobasidin A (AbA). Pro/pGADT7 and Pro/pGADT7‐TaMYB4‐7D mark the yeast cells carrying *TaPHT1;9‐4B* promoter with the empty pGADT7 vector or the construct pGADT7‐TaMYB4‐7D. (c) Diagrams of effector and reporter constructs used in dual‐luciferase assay. (d) Ratios of LUC to REN activities obtained in the absence or presence of TaMYB4‐7D. (e, f) Binding of TaMYB4‐7D to the four putative MBSs in *TaPHT1;9‐4B* promoter. Y1H assay, conducted as above, showed that P1–P4, but not their mutants (mP1–mP4) (e), interacted with *TaPHT1;9‐4B* promoter (f). (g–j) Examination of TaMYB4 silenced wheat plants. The plants infected by BSMV‐GFP or BSMV‐TaMYB4, cultured under Pi‐sufficient (1 mM), low‐Pi (50 μM) or Pi‐deficient (0 μM) Hoagland solutions for 10 d, were examined for shoot and root lengths (g), dry weights (h) and P concentrations (i), as well as *TaPHT1;9* transcript levels in leaf and root tissues. Data represent means ± SD of three biological replicates. Asterisks indicate statistically significant differences (*, *P* < 0.05; Student’s *t*‐test).

*TaPHT1;9‐4B* promoter carried four predicted MYB TF binding sites (MBSs, P1 to P4) (Figs 5a, S15). Y1H assay showed that the yeast cells harbouring this promoter (bait) and the pGADT7‐TaMYB4 vector (prey) grew well on the SD/−Leu medium containing different concentrations (200, 300 and 500 ng ml^−1^) of aureobasidin A (Fig. 5b). Furthermore, dual‐luciferase assay showed that TaMYB4‐7D could bind to, and activate, the promoter of *TaPHT1;9‐4B* in tobacco leaf cells (Fig. 5c,d). To further characterise the binding sites of TaMYB4‐7D, the four potential MBSs in *TaPHT1;9‐4B* promoter, that is P1–P4, were individually mutated (Fig. 5e), followed by cloning into the bait vector. The combination of TaMYB4‐7D with P1–P4, but not with their mutants (mP1 to mP4), enabled the growth of yeast cells on the SD/−Leu medium containing 150 ng ml^−1^ aureobasidin A (Fig. 5f), therefore validating the binding of TaMYB4‐7D to P1–P4 inside the cells.

Under Pi‐deficient conditions, *TaMYB4* transcript levels were substantially upregulated in both root and shoot tissues (Fig. S16). To investigate the effects of TaMYB4‐7D on wheat response to low‐Pi supply, BSMV‐VIGS was used to silence its expression. A cDNA fragment (217 bp) of *TaMYB4‐7D* was used to develop the silencing virus BSMV‐MYB4 (Fig. S13a). The wheat plants infected by BSMV‐MYB4 showed chlorosis and decreased expression of *TaMYB4* (Fig. S17a,b), and exhibited enhanced sensitivity to low‐Pi or Pi‐deficiency stresses compared with the controls inoculated with BSMV‐GFP (Fig. S17c). Relative to the controls, BSMV‐MYB4‐inoculated plants exhibited notable decreases in root and shoot lengths and dry weights (Fig. 5g,h); they also exhibited significant reductions in P concentrations in the presence of 1 mM or 50 μM external Pi (Fig. 5i). Under Pi‐deficiency (0 μM Pi) conditions, P concentration in the shoots of BSMV‐MYB4‐infected plants remained lower relative to the controls, but P concentration in the roots of these plants was higher (Fig. 5i). The expression levels of *TaPHT1;9* were strongly upregulated by decreasing Pi supply in the roots of control plants, but this induction was clearly attenuated in the plants infected by BSMV‐MYB4 (Fig. 5j). *TaPHT1;9* expression was also reduced in the shoots of BSMV‐MYB4‐infected plants compared with the controls, although in this organ *TaPHT1;9* was expressed at a much lower level than in the roots, and was not highly induced by decreasing Pi supply (Fig. 5j). Obviously, silencing *TaMYB4* decreased Pi uptake from the external environment and Pi translocation from the root to shoot under Pi‐deficiency conditions. Additionally, the foliar anthocyanin contents of BSMV‐MYB4‐infected plants were significantly decreased compared with those of controls under Pi‐deprived conditions (Fig. S18). In line with this result, the transcript levels of four anthocyanin biosynthesis genes (*TaCHS*, *TaCHI*, *TaDFR* and *TaANS*) were significantly downregulated in the leaves of *TaMYB4*‐silenced plants compared with the controls (Fig. S19).

### Nucleotide diversity and haplotype analysis of *TaPHT1;9‐4B*

**Fig. 6:**
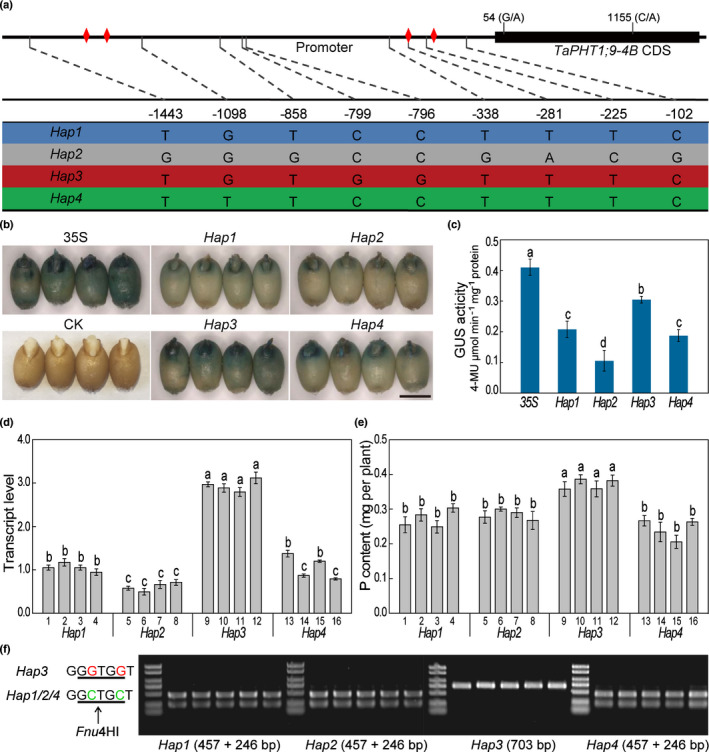
Analysis of the molecular haplotypes of *TaPHT1;9‐4B* promoter. (a) The four haplotypes (*Hap1*, *Hap2*, *Hap3* and *Hap4*) of *TaPHT1;9‐4B* promoter identified based on nine SNPs located in the 5′ proximal region (−1513 bp to +1 bp, relative to the translation start codon ATG). The two SNPs, that is G/A (54) and C/A (1155), in the coding sequence were also shown, although they did not cause amino acid changes. The four predicted MBSs were indicated by red diamonds. (b, c) Comparison of the activities of four promoter haplotypes based on the their potency to drive GUS expression after being introduced into germinating wheat grains by particle bombardment. The 35S promoter was used as a positive control. Unbombarded WT grains were used as a negative control (CK) for GUS staining. Bar, 5 mm. (d, e) Transcript levels of *TaPHT1;9‐4B* and P contents under low‐Pi (50 μM) conditions in four sets of bread wheat cultivars (1–16, four lines per set, Supporting Information Table S1) with different *TaPHT1;9‐4B* promoter haplotypes. Data represent means ± SD of three biological replicates. Different letters indicate statistically significant differences (*P* < 0.05, one‐way ANOVA followed by Duncan's multiple range test). (f) Development of a CAPS marker for *TaPHT1;9‐4B* promoter haplotype *Hap3* by digesting PCR amplicons with the restriction endonuclease *Fnu*4HI (arrow).

Nucleotide diversities in the promoter and coding regions of *TaPHT1;9‐4B* were investigated in 62 bread wheat cultivars, which led to the finding of nine single nucleotide polymorphisms (SNPs) in the promoter and two SNPs in the coding region (Fig. 6a). Nucleotide diversity (π) values of the two regions in the examined bread wheat lines were 1.33 × 10^−3^ and 7.10 × 10^−4^ (Fig. S20). By contrast, the orthologues of *TaPHT1;9‐4B* in tetraploid wheat (*TaPHT1;9‐4B^Tt^*) and four S genome‐containing *Aegilops* species (*TaPHT1;9‐4S*) showed much higher genetic diversities. For *TaPHT1;9‐4B^Tt^*, the π values of promoter and coding regions were 5.13 × 10^−3^ and 2.73 × 10^−3^, respectively (Fig. S20). For *TaPHT1;9‐4S*, the corresponding π values were 2.07 × 10^−2^ and 1.81 × 10^−2^ (Fig. S20). The genetic diversity of this gene showed a clear decline during the evolution from diploid to polyploid species.

The two SNPs (54 G/A and 1155 C/A) in the coding sequence of *TaPHT1;9‐4B* did not cause amino acid change. We therefore focused on the nine SNPs in its promoter region. The nine SNPs formed four promoter haplotypes (*Hap1*–*Hap4*) among the cultivars analysed in this work (Fig. 6a). Compared with *Hap1*, *Hap2*, *Hap3* and *Hap4* contained six, two and one SNPs, respectively (Fig. 6a). The promoter activities of the four haplotypes were tested by their potency to drive GUS expression in germinating wheat grains, The results showed that the *Hap3* promoter conferred the highest GUS signals, followed by *Hap1* and *Hap4* promoters, with the *Hap2* promoter showing the lowest activity (Fig. 6b,c). Among the six SNPs of *Hap2*, one (−281) occurred in a putative MBS element (Fig. 6a), which might be responsible for its decreased promoter activity.

The above results prompted us to investigate if the four promoter haplotypes may be associated with differences in *TaPHT1;9‐4B* expression level and P accumulation in bread wheat cultivars. To test this possibility, we selected four modern bread wheat cultivars for each haplotype (Table S1), and cultured their seedlings under low‐Pi (50 μM) conditions. Among the cultivars tested, the *Hap3* promoter was associated with the highest *TaPHT1;9‐4B* expression level, better growth performance with higher biomass, and elevated P content in whole plants (Figs 6d,e, S21a–c). However, P concentrations did not differ substantially among the promoter haplotypes (Fig. S21d), implying that the *Hap3* type of cultivars could efficiently utilised P to promote plant growth.

To distinguish *Hap3* from the other three haplotypes, we developed a CAPS marker based on the two SNPs at positions −799 (C/G) and −796 (C/G) (Fig. 6a). A 703‐bp fragment was amplified from the wheat cultivars examined in this work using TaPHT1;9‐4B‐CAPS‐799 F/R primers (Table S2), with the resulting sequence data described in Fig. S22. Digestion by *Fnu*4HI restriction endonuclease cleaved the amplicons of *Hap1*, *Hap2* and *Hap4* into two fragments (457 and 246 bp), whereas no cleavage occurred to the amplicons from *Hap3* (Fig. 6f). We also investigated the genetic diversity of *TaMYB4‐7D*, which exhibited no polymorphism in either its promoter or coding regions in the 62 bread wheat cultivars examined.

## Discussion

In this study, we characterised TaPHT1;9‐4B and its transcriptional regulator TaMYB4‐7D to increase the understanding of bread wheat PHT1s and to gain functional insight into these important proteins in crop plants.

### TaPHT1 proteins functioning in Pi uptake and translocation in bread wheat roots

It is well known that bread wheat carries a large and complex family of *PHT1* genes (Teng *et al*., 2017; Grün *et al*., 2018). Here we identified four TaPHT1s (i.e. TaPHT1;3‐5B, TaPHT1;6‐5B, TaPHT1;9‐4B and TaPT2) whose protein levels were significantly upregulated in the roots of an elite bread wheat cultivar by Pi deficiency using quantitative proteomic analysis. The presence of TaPT2 among the four Pi‐deficiency upregulated TaPHT1s (Fig. 1d) is consistent with the important function of this transport in Pi uptake and plant growth in wheat (Davies *et al*., 2002; Zeng *et al*., 2002; Guo *et al*., 2014). More importantly, we conducted new functional analysis on TaPHT1;9‐4B using a variety of genetic and molecular methods. Based on the data gathered in this and previous studies, several suggestions can be made on the TaPHT1 proteins functioning in Pi uptake and translocation in bread wheat.

First, TaPHT1;9‐4B is an active high‐affinity Pi transporter predominantly expressed in bread wheat roots, and is required for maintaining Pi uptake and wheat plant growth under both Pi‐sufficient and Pi‐limiting conditions. This is supported by its complementation of *pho84* mutation in yeast cells, strong induction of *TaPHT1;9* expression in the roots by decreased Pi supply, and substantial reductions in the growth of wheat shoots and roots when TaPHT1;9 function was disrupted by BSMV‐VIGS or CRISPR/Cas9 editing. Furthermore, transgenic overexpression of *TaPHT1;9‐4B* in rice improved plant growth and P accumulation under both Pi‐sufficient and Pi‐deprived conditions, therefore adding further evidence on the active role of TaPHT1;9‐4B in mediating Pi uptake and promoting plant growth. It is worth noting that under Pi‐deficiency conditions (0 μM Pi), disrupting TaPHT1;9 function by either BSMV‐VIGS or CRISPR editing led to higher accumulation of P in the roots than in the shoots (Figs 3d, 4f), whereas the reverse was observed when *TaPHT1;9‐4B* was overexpressed in rice (Fig. 3h). This indicates that TaPHT1;9‐4B functions in Pi transport from root to shoot. In agreement with our results, previous research also showed that *TaPHT1;9* (*TaPht1;1a*) was mainly expressed in bread wheat roots and upregulated by low‐Pi treatment (Teng *et al*., 2013, 2017; Grün *et al*., 2018), although in these studies no efforts were made to analyse the function of specific *TaPHT1;9* homoeologs.

Second, TaPHT1;9‐4B shows both functional similarity and difference with TaPT2. TaPT2 is also primarily expressed in bread wheat roots, partially complements *pho84* mutation in yeast cells, and can improve (suppress) plant growth and P accumulation when overexpressed (silenced) (Davies *et al*., 2002; Zeng *et al*., 2002; Guo *et al*., 2014). Therefore, TaPHT1;9‐4B and TaPT2 may act similarly in Pi uptake in bread wheat roots under low P conditions. This functional similarity is additionally supported by the high amino acid sequence identity between the two proteins (99.04% identical, Table S4) and tight clustering of the two proteins in phylogenetic tree. However, TaPHT1;9‐4B functions under both Pi‐sufficient and Pi‐deprived conditions, whereas TaPT2 does not appear to do so because altering its expression in transgenic plants did not affect plant growth and P level when Pi supply was adequate (Guo *et al*., 2014).

Finally, multiple TaPHT1s are activated in the roots of Pi‐starved bread wheat plants. In addition to TaPHT1;9‐4B and TaPT2, the protein levels of TaPHT1;3‐5B and TaPHT1;6‐5B were also upregulated by Pi deficiency, indicating the function of multiple TaPHT1 proteins in Pi‐starved wheat roots. Moreover, *TaPHT1;3* and *TaPHT1;6* may compensate for the function of *TaPHT1;9* because they were upregulated in *TaPHT1;9* silenced wheat plants (Fig. S8). Of the 13 *OsPHT1* genes, eight were transcriptionally elevated in rice roots in response to decreased Pi supply (Secco *et al*., 2013). Among the nine *AtPHT1* genes, eight were expressed in the roots (Ayadi *et al*., 2015; Młodzińska & Zboińska, 2016). Therefore, the rich knowledge on rice and *Arabidopsis* PHT1 proteins may aid further functional analysis of the four TaPHT1s activated by Pi deprivation in wheat roots.

### Regulation of TaPHT1;9‐4B transcription by TaMYB4‐7D

In bread wheat, only one MYB family TF, TaPHR1, has so far been demonstrated to upregulate a subset of PSI genes and to enhance Pi uptake and root growth when overexpressed in transgenic plants (Wang *et al*., 2013). Here we isolated TaMYB4‐7D and generated several lines of evidence suggesting that TaMYB4‐7D positively regulates the transcription of TaPHT1;9‐4B in bread wheat. First, TaMYB4‐7D was located in the nucleus and possessed transcription activation properties. Second, TaMYB4‐7D could bind to the four MBSs in the promoter region of TaPHT1;9‐4B in a cellular environment. Third, silencing *TaMYB4‐7D* expression decreased wheat plant growth and P accumulation, which resembled highly the negative effects brought about by reducing the expression of *TaPHT1;9‐4B*. Finally, the upregulation of *TaPHT1;9‐4B* expression by low‐Pi or Pi‐deficiency treatment was strongly attenuated in *TaMYB4‐7D* silenced wheat plants. Therefore, it is likely that TaMYB4‐7D and TaPHT1;9‐4B may form a functional module that takes an active part in Pi uptake in wheat, especially in the roots where TaPHT1;9‐4B is predominantly expressed and significantly elevated by inadequate Pi supply. Other TFs, such as PHR and WRKY proteins, may also contribute to the control of TaPHT1;9‐4B expression as P1BS and W‐box, to which PHR and WRKY TFs bind, respectively, are present in the promoter region of many *TaPHT1* genes, including *TaPHT1;9* (*TaPht1;1a*) homoeologs (Grün *et al*., 2018).

Two studies in rice suggest that the R2R3 MYB TFs OsMYB4P and OsMYB5P can positively regulate the transcription of multiple OsPHT1s (Yang *et al*., 2014, 2018). For OsMYB5P, direct binding of OsMYB5P to the promoter region of nine *OsPHT1* genes was demonstrated using ChIP assay (Yang *et al*., 2018). In this context, it will be interesting to study if TaMYB4‐7D may also regulate the expression of the other three Pi‐deficiency upregulated TaPHT1s. In a preliminary analysis, MBSs were found to be present in the promoter region of *TaPHT1;3‐5B*, *TaPHT1;6‐5B* and *TaPT2*, and TaMYB4‐7D could interact with, and activate, the promoter of the three genes (Figs S23, S24). These data reinforce the importance of TaMYB4‐7D in the regulation of TaPHT1s.

Ma *et al*. (2011) showed that TaMYB4 (TaMYB4‐7D) could repress the genes involved in lignin biosynthesis by binding to the AC‐II *cis*‐element, which suppressed lignin accumulation but stimulated flavonoid biosynthesis. P deficiency often leads to strong upregulation of flavonoid and anthocyanin productions in higher plants (Raghothama, 1999; Wang *et al*., 2018b). Under Pi‐deprived conditions, the silencing of *TaMYB4* inhibited efficient anthocyanin accumulation and the expression of several anthocyanin biosynthesis genes (Figs S18, S19). Therefore, we speculated that TaMYB4 may play a dual role under Pi‐limiting conditions, that is promoting Pi uptake by increasing the expression of PHT1s and increasing flavonoid and anthocyanin biosynthesis by elevating the expression of anthocyanin biosynthesis genes but suppressing those involved in lignin accumulation.

### Efficient identification of Pi‐deficiency regulated wheat proteins using iTRAQ‐based quantitative proteomics

The iTRAQ‐based proteomic analysis is emerging as an efficient approach for uncovering the pathways and proteins functioning in plant responses to changing Pi supplies. In Arabidopsis, iTRAQ analysis permitted the quantification of >10 000 proteins, and clarified the involvement of chromatin reorganisation and redox homeostasis in the regulation of root growth (Suen *et al*., 2018), and identified flavonoid biosynthesis as the most significantly enhanced metabolic process, under Pi‐starvation conditions (Wang *et al*., 2018b). In rice, iTRAQ analysis has facilitated the functional characterisation of two vacuolar phosphate efflux transporters (Xu *et al*., 2019), and the roles of multiple types of kinases, in Pi‐starved plants (Yang *et al*., 2019). Here we used this approach and identified 4306 PDRPs with 1015 of them mapped to specific homoeologs in A, B or D subgenomes. These PDRPs may become a valuable resource for systematically dissecting the complex proteomic changes required for bread wheat to adapt to low‐Pi availability.

Markedly, the majority of the 1015 assigned PDRPs were the products of single homoeologs, with no expression detected for their remaining homoeologs in our iTRAQ analysis. Therefore, homoeolog expression bias may underpin a large proportion of the bread wheat proteome under Pi‐deficiency conditions. A previous transcriptomic study on bread wheat plants infected by a fungal pathogen also reported that a high proportion of the biotic stress responsive gene expression resulted from single homoeologs from one subgenome alone (subgenomes A, B or D) (Powell *et al*., 2017). The finding of homoeolog expression bias in our work provides a new direction for studying mechanism underlying the dynamic gene expression patterns regulated by Pi availability in wheat.

### Haplotype variation and potential value of TaPHT1;9‐4B in improving Pi uptake and use efficiencies in bread wheat

By examining the promoter and coding regions of *TaPHT1;9‐4B* and its orthologues in hexaploid and tetraploid wheat lines and diploid relatives, a dramatic decrease in nucleotide diversity was observed, suggesting that strong selection occurred on *TaPHT1;9‐4B* during the evolution of polyploid wheat. Nevertheless, the promoter region of *TaPHT1;9‐4B* displayed a moderate level of haplotype variation, with *Hap3* showing significant positive associations with *TaPHT1;9‐4B* transcript levels, growth performance and P content in bread wheat cultivars. Considering the active role of TaPHT1;9‐4B in Pi acquisition as demonstrated by this work, it is necessary to test its potential value in improving wheat P acquisition and use efficiencies. The CAPS marker, *CAPS‐799*, capable of distinguishing *Hap3* from the other three haplotypes, may be used to transfer the *Hap3* allele of *TaPHT1;9‐4B* into the elite bread wheat background to evaluate its effects on P acquisition and plant growth. Alternatively, transgenic expression may be used to explore the breeding values of TaPHT1;9‐4B and its transcriptional regulator TaMYB4‐7D, as ectopic overexpression of certain PHT1 proteins and regulatory TFs has been shown to enhance P accumulation, root and shoot biomass and yield performance in crop plants (Liu *et al*., 2013; Wang *et al*., 2013; Guo *et al*., 2014; Yan *et al*., 2014; Zhang *et al*., 2014; Qu *et al*., 2015; Dai *et al*., 2016; Yang *et al*., 2016; Kopriva & Chu, 2018).

In summary, our work has uncovered four PHT1 proteins upregulated in bread wheat roots by Pi deficiency. The high‐affinity transporter TaPHT1;9‐4B and its transcriptional regulator TaMYB4‐7D contributed to efficient Pi acquisition and plant growth under Pi‐limiting conditions. This two genes may stimulate further research into the molecular mechanism controlling Pi homoeostasis and utilisation in wheat, which together with the superior promoter haplotype of *TaPHT1;9‐4B* may facilitate the development of P efficient wheat cultivars in the future.

## Accession numbers

The amplified sequences of *TaPHT1;9‐4B* homoeolog for diploids, and tetraploids have been submitted to GenBank (accession nos. MN043994–MN044002). Proteomic data were submitted to ProteomeXchange with the dataset identifier PXD003570.

## Author contributions

GZK conceived the project. PFW performed and analysed the experiments. GZL, GWL and DWW helped with data analysis. SSY helped with the preparation of wheat materials. CYW, YXX and TCG evaluated wheat cultivars. DWW, PFW and GZK wrote the manuscript.

## Supporting information


**Dataset S1** Identification and analysis of phosphate deficiency responsive proteins (PDRPs) in bread wheat roots using iTRAQ‐based proteomics.
**Dataset S2** Quantification analysis of 11 Pi‐deficiency responsive proteins (PDRPs) using PRM analysis in bread wheat roots.
**Dataset S3** Mapping PDRPs to the genomic loci of Chinese Spring.
**Fig. S1** Examples illustrating homoeolog mapping of proteomic peptides in this work.
**Fig. S2** Phenotype and growth parameters of wheat seedlings cultured under Pi‐deficient conditions for 10 d.
**Fig. S3** Comparison of 11 PDRPs with respect to their expression changes induced by Pi deficiency revealed using iTRAQ or PRM approaches.
**Fig. S4** Nucleotide and deduced amino acid sequence of *TaPHT1;9‐4B*.
**Fig. S5** Transcript level of *TaPHT1;9* in the root and shoot tissues of the wheat plants cultured under Pi‐sufficient or ‐deficient media for 8 d.
**Fig. S6** The coding sequences of *TaPHT1;9* homoeologs (4A, 4B and 4D) in Chinese Spring.
**Fig. S7** Functional analysis of *TaPHT1;9* in bread wheat using BSMV‐VIGS.
**Fig. S8** Evaluation of the transcript levels of three *TaPHT1* genes and *TaIPS1.1* in the roots of the wheat plants infected by BSMV‐GFP or BSMV‐TaPHT1;9.
**Fig. S9** Molecular identification of transgenic rice lines expressing *TaPHT1;9‐4B*.
**Fig. S10** The effects of nucleotide mutations in three CRISPR mutants on TaPHT1;9‐4B protein.
**Fig. S11** Phenotypes, dry weights and P concentrations of three CRISPR mutants and WT Fielder control cultured under Pi‐sufficient conditions.
**Fig. S12** Y1H screening using *TaPHT1;9‐4B* promoter as bait.
**Fig. S13** Sequences and phylogenetic tree of TaMYB4‐7D.
**Fig. S14** Chromosomal location, subcellular localisation and transcriptional activation activities of TaMYB4‐7D.
**Fig. S15** Sequence of *TaPHT1;9‐4B* promoter.
**Fig. S16** Transcript level of *TaMYB4* in the root and shoot tissues of the wheat plants cultured under Pi‐sufficient or ‐deficient media for 8 d.
**Fig. S17** Analysis of the bread wheat plants with *TaMYB4* expression silenced by BSMV‐VIGS.
**Fig. S18** Evaluation of foliar anthocyanin contents in the wheat plants infected by BSMV‐GFP or BSMV‐TaMYB4.
**Fig. S19** Evaluation of the transcript levels of four anthocyanin biosynthesis genes in the leaves of BSMV‐GFP‐ or BSMV‐TaMYB4‐infected wheat plants.
**Fig. S20** Analysis of nucleotide diversity of *PHT1;9‐4B* promoter and its genomic coding sequence in bread wheat and relatives.
**Fig. S21** Phenotypes, dry weights, P contents and P concentrations of the 16 wheat varieties with different promoter haplotypes of *TaPHT1;9‐4B* under low‐Pi conditions.
**Fig. S22** Nucleotide sequence comparison of the DNA fragments used to differentiate four *TaPHT1;9‐4B* promoter haplotypes (*Hap1* to *Hap4*).
**Fig. S23** Nucleotide sequence of the promoter region of *TaPHT1;3‐5B*, *TaPHT1;6‐5B* and *TaPT2*.
**Fig. S24** Binding of TaMYB4‐7D to the promoter region of *TaPHT1;3‐5B*, *TaPHT1;6‐5B* and *TaPT2*.
**Methods S1** Additional description of methods.
**Table S1** Accessions of hexaploid bread wheat and its relative species used in this study.
**Table S2** All primers used in this study.
**Table S3** Accession numbers of PHT proteins for constructing phylogenetic tree of TaPHT1;9‐4B.
**Table S4** Identities of PHT proteins used for constructing the phylogenetic tree of TaPHT1;9‐4B.
**Table S5** Potential proteins interacting with the promoter of TaPHT1;9‐4B obtained using Y1H screening.
Please note: Wiley Blackwell are not responsible for the content or functionality of any Supporting Information supplied by the authors. Any queries (other than missing material) should be directed to the *New Phytologist* Central Office.

## Data Availability

The data and resources supporting the findings of this study are available on request from the corresponding authors.

## References

[nph17534-bib-0001] Ai P , Sun S , Zhao J , Fan X , Xin W , Guo Q , Yu L , Shen Q , Wu P & Miller AJ & *et al*. 2009. Two rice phosphate transporters, OsPht1;2 and OsPht1;6, have different functions and kinetic properties in uptake and translocation. The Plant Journal 57 798–809. 18980647 10.1111/j.1365-313X.2008.03726.x

[nph17534-bib-0002] Ajmera I , Hodgman TC , Lu C . 2019. An integrative systems perspective on plant phosphate research. Genes 10: 139. 30781872 10.3390/genes10020139 PMC6410211

[nph17534-bib-0003] Ayadi A , David P , Arrighi JF , Chiarenza S , Thibaud MC , Nussaume L , Marin E . 2015. Reducing the genetic redundancy of *Arabidopsis* phosphate transporter1 transporters to study phosphate uptake and signaling. Plant Physiology 167 1511–1526. 25670816 10.1104/pp.114.252338 PMC4378149

[nph17534-bib-0004] Aziz T , Finnegan PM , Lambers H , Jost R . 2014. Organ‐specific phosphorus‐allocation patterns and transcript profiles linked to phosphorus efficiency in two contrasting wheat genotypes. Plant, Cell & Environment 37: 943–960. 10.1111/pce.12210 24191900

[nph17534-bib-0005] Bun‐Ya M , Nishimura M , Harashima S , Oshima Y . 1991. The *PHO84* gene of *Saccharomyces cerevisiae* encodes an inorganic phosphate transporter. Molecular and Cell Biology 11: 3229–3238. 10.1128/mcb.11.6.3229-3238.1991 PMC360175 2038328

[nph17534-bib-0006] Chang MX , Gu M , Xia YW , Dai XL , Dai CR , Zhang J , Wang SC , Qu HY , Yamaji N , Ma JF . 2019. OsPHT1;3 mediates uptake, translocation, and remobilization of phosphate under extremely low phosphate regimes. Plant Physiology 179: 656–670. 30567970 10.1104/pp.18.01097 PMC6426419

[nph17534-bib-0007] Chen CY , Schmidt W . 2015. The paralogous R3 MYB proteins caprice, triptychon and enhancer of TRY and CPC1 play pleiotropic and partly non‐redundant roles in the phosphate starvation response of *Arabidopsis* roots. Journal of Experimental Botany 66: 4821–4834. 26022254 10.1093/jxb/erv259 PMC4507782

[nph17534-bib-0008] Dai X , Wang Y , Yang A , Zhang WH . 2012. OsMYB2P‐1, an R2R3 MYB transcription factor, is involved in the regulation of phosphate‐starvation responses and root architecture in rice. Plant Physiology 159: 169–183. 22395576 10.1104/pp.112.194217 PMC3375959

[nph17534-bib-0009] Dai X , Wang Y , Zhang WH . 2016. OsWRKY74, a WRKY transcription factor, modulates tolerance to phosphate starvation in rice. Journal of Experimental Botany 67: 947–960. 26663563 10.1093/jxb/erv515 PMC4737085

[nph17534-bib-0010] Davies TGE , Ying J , Xu Q , Li ZS , Li J , Gordon‐Weeks R . 2002. Expression analysis of putative high‐affinity phosphate transporters in Chinese winter wheats. Plant, Cell & Environment 25: 1325–1339.

[nph17534-bib-0011] Deng Y , Teng W , Tong YP , Chen XP , Zou CQ . 2018. Phosphorus efficiency mechanisms of two wheat cultivars as affected by a range of phosphorus levels in the field. Frontiers in Plant Science 9: 1614. 30459796 10.3389/fpls.2018.01614 PMC6232341

[nph17534-bib-0012] Feldman M , Levy AA . 2012. Genome evolution due to allopolyploidization in wheat. Genetics 192: 763–774. 23135324 10.1534/genetics.112.146316 PMC3522158

[nph17534-bib-0013] Grierson CS , Barnes SR , Chase MW , Clarke M , Grierson D , Edwards KJ , Jellis GJ , Jones JD , Knapp S , Oldroyd G *et al*. 2011. One hundred important questions facing plant science research. New Phytologist 192: 6–12. 21883238 10.1111/j.1469-8137.2011.03859.x

[nph17534-bib-0014] Grün A , Buchner P , Broadley MR , Hawkesford MJ . 2018. Identification and expression profiling of Pht1 phosphate transporters in wheat in controlled environments and in the field. Plant Biology 20: 374–389. 29148171 10.1111/plb.12668 PMC5887882

[nph17534-bib-0015] Gu M , Chen A , Sun S , Xu G . 2016. Complex regulation of plant phosphate transporters and the gap between molecular mechanisms and practical application: what is missing? Molecular Plant 9: 396–416. 26714050 10.1016/j.molp.2015.12.012

[nph17534-bib-0016] Gu M , Zhang J , Li H , Meng D , Li R , Dai X , Wang S , Liu W , Qu H , Xu G . 2017. Maintenance of phosphate homeostasis and root development are coordinately regulated by MYB1, an R2R3‐type MYB transcription factor in rice. Journal of Experimental Botany 68: 3603–3615. 28549191 10.1093/jxb/erx174 PMC5853628

[nph17534-bib-0017] Guo CJ , Guo L , Li XJ , Gu JT , Zhao M , Duan WW , Ma C , Lu W , Xiao K . 2014. TaPT2, a high‐affinity phosphate transporter gene in wheat (*Triticum aestivum* L.), is crucial in plant Pi uptake under phosphorus deprivation. Acta Physiology Plantrum 36: 1373–1384.

[nph17534-bib-0018] Guo M , Ruan W , Li C , Huang F , Zeng M , Liu Y , Yu Y , Ding X , Wu Y , Wu Z *et al*. 2015. Integrative comparison of the role of the phosphate response1 subfamily in phosphate signaling and homeostasis in rice. Plant Physiology 168: 1762–1776. 26082401 10.1104/pp.15.00736 PMC4528768

[nph17534-bib-0019] Heuer S , Gaxiola R , Schilling R , Herrera‐Estrella L , López‐Arredondo D , Wissuwa M , Delhaize E , Rouached H . 2017. Improving phosphorus use efficiency: a complex trait with emerging opportunities. The Plant Journal 90: 868–885. 27859875 10.1111/tpj.13423

[nph17534-bib-0020] Hu G , Koh J , Yoo MJ , Pathak D , Chen S , Wendel J . 2014. Proteomics profiling of fiber development and domestication in upland cotton (*Gossypium hirsutum* L.). Planta 240: 1237–1251. 25156487 10.1007/s00425-014-2146-7

[nph17534-bib-0021] International Wheat Genome Sequencing Consortium (IWGSC) , Appels R , Eversole K , Feuillet C , Keller B , Rogers J , Stein N , Pozniak CJ , Stein N , Choulet F *et al*. 2018. Shifting the limits in wheat research and breeding using a fully annotated reference genome. Science 361: 661. 10.1126/science.aar7191 30115783

[nph17534-bib-0022] Jia H , Ren H , Gu M , Zhao J , Sun S , Zhang X , Chen J , Wu P , Xu G . 2011. The phosphate transporter gene *OsPht1;8* is involved in phosphate homeostasis in rice. Plant Physiology 156: 1164–1175. 21502185 10.1104/pp.111.175240 PMC3135946

[nph17534-bib-0023] Kopriva S , Chu C . 2018. Are we ready to improve phosphorus homeostasis in rice? Journal of Experimental Botany 69: 3515–3522. 29788117 10.1093/jxb/ery163

[nph17534-bib-0024] Li G , Wu Y , Liu G , Xiao X , Wang P , Gao T , Xu M , Han Q , Wang Y , Guo T *et al*. 2017. Large‐scale proteomics combined with transgenic experiments demonstrates an important role of jasmonic acid in potassium deficiency response in wheat and rice. Molecular & Cellular Proteomics 16: 1889–1905. 28821602 10.1074/mcp.RA117.000032 PMC5671998

[nph17534-bib-0064] Liu H , Wang K , Jia Z , Gong Q , Liu, Z , Du L , Pei X , Ye X . 2020. Efficient induction of haploid plants in wheat by editing of *TaMTL* using an optimized *Agrobacterium*‐mediated CRISPR system. Journal of Experimental Botany 71: 1337–1349. 31760434 10.1093/jxb/erz529 PMC7031065

[nph17534-bib-0025] Liu XM , Zhao XL , Zhang LJ , Lu WJ , Li XJ , Xiao K . 2013. *TaPht1;4*, a high‐affinity phosphate transporter gene in wheat (*Triticum aestivum*), plays an important role in plant phosphate acquisition under phosphorus deprivation. Functional Plant Biology 40: 329–341. 32481111 10.1071/FP12242

[nph17534-bib-0026] Ma QH , Wang C , Zhu HH . 2011. TaMYB4 cloned from wheat regulates lignin biosynthesis through negatively controlling the transcripts of both cinnamyl alcohol dehydrogenase and cinnamoyl‐CoA reductase genes. Biochimie 93: 1179–1186. 21536093 10.1016/j.biochi.2011.04.012

[nph17534-bib-0027] Ma X , Zhang Q , Zhu Q , Liu W , Chen Y , Qiu R , Wang B , Yang Z , Li H , Lin Y *et al*. 2015. A robust CRISPR/Cas9 system for convenient, high‐efficiency multiplex genome editing in monocot and dicot plants. Molecular Plant 8: 1274–1284. 25917172 10.1016/j.molp.2015.04.007

[nph17534-bib-0028] McGinnis S , Madden TL . 2004. BLAST: at the core of a powerful and diverse set of sequence analysis tools. Nucleic Acids Research 32: W20–W25. 15215342 10.1093/nar/gkh435 PMC441573

[nph17534-bib-0029] Medina J , Ballesteros ML , Salinas J . 2007. Phylogenetic and functional analysis of *Arabidopsis RCI2* genes. Journal of Experimental Botany 58: 4333–4346. 18182435 10.1093/jxb/erm285

[nph17534-bib-0030] Młodzińska E , Zboińska M . 2016. Phosphate uptake and allocation‐A closer look at *Arabidopsis thaliana* L. and *Oryza sativa* L. Frontiers in Plant Science 7: 1198. 27574525 10.3389/fpls.2016.01198 PMC4983557

[nph17534-bib-0031] Powell JJ , Fitzgerald TL , Stiller J , Berkman PJ , Gardiner DM , Manners JM , Henry RJ , Kazan K . 2017. The defence‐associated transcriptome of hexaploid wheat displays homoeolog expression and induction bias. Plant Biotechnology Journal 15: 533–543. 27735125 10.1111/pbi.12651 PMC5362679

[nph17534-bib-0032] Qu B , He X , Wang J , Zhao Y , Teng W , Shao A , Zhao X , Ma W , Wang J , Li B *et al*. 2015. A wheat CCAAT box‐binding transcription factor increases the grain yield of wheat with less fertilizer input. Plant Physiology 167: 411–423. 25489021 10.1104/pp.114.246959 PMC4326744

[nph17534-bib-0033] Raghothama KG . 1999. Phosphate acquisition. Annual Review of Plant Physiology and Plant Molecular Biology 50: 665–693. 10.1146/annurev.arplant.50.1.665 15012223

[nph17534-bib-0034] Ruan W , Guo M , Wu P , Yi K . 2017. Phosphate starvation induced OsPHR4 mediates Pi‐signaling and homeostasis in rice. Plant Molecular Biology 93: 327–340. 27878661 10.1007/s11103-016-0564-6

[nph17534-bib-0035] Rubio V , Linhares F , Solano R , Martín AC , Iglesias J , Leyva A , Paz‐Ares J . 2001. A conserved MYB transcription factor involved in phosphate starvation signaling both in vascular plants and in unicellular algae. Genes & Development 15: 2122–2133. 11511543 10.1101/gad.204401 PMC312755

[nph17534-bib-0036] Secco D , Baumann A , Poirier Y . 2010. Characterization of the rice *PHO1* gene family reveals a key role for *OsPHO1;2* in phosphate homeostasis and the evolution of a distinct clade in dicotyledons. Plant Physiologist 152: 1693–1704. 10.1104/pp.109.149872 PMC2832267 20081045

[nph17534-bib-0037] Secco D , Bouain N , Rouached A , Prom‐U‐Thai C , Hanin M , Pandey AK , Rouached H . 2017. Phosphate, phytate and phytases in plants: from fundamental knowledge gained in Arabidopsis to potential biotechnological applications in wheat. Critical Reviews in Biotechnology 37: 898–910. 28076998 10.1080/07388551.2016.1268089

[nph17534-bib-0038] Secco D , Jabnoune M , Walker H , Shou H , Wu P , Poirier Y , Whelan J . 2013. Spatio‐temporal transcript profiling of rice roots and shoots in response to phosphate starvation and recovery. Plant Cell 25: 4285–4304. 24249833 10.1105/tpc.113.117325 PMC3875719

[nph17534-bib-0039] Shewry PR , Hey SJ . 2015. The contribution of wheat to human diet and health. Food and Energy Security 4: 178–202. 27610232 10.1002/fes3.64 PMC4998136

[nph17534-bib-0040] Shukla V , Kaur M , Aggarwal S , Bhati KK , Kaur J , Mantri S , Pandey AK . 2016. Tissue specific transcript profiling of wheat phosphate transporter genes and its association with phosphate allocation in grains. Scientific Reports 6: 39293. 27995999 10.1038/srep39293 PMC5172359

[nph17534-bib-0041] de Souza Campos PM , Cornejo P , Rial C , Borie F , Varela RM , Seguel A , López‐Ráez JA . 2019. Phosphate acquisition efficiency in wheat is related to root: shoot ratio, strigolactone levels, and PHO2 regulation. Journal of Experimental Botany 70: 5631–5642. 31359044 10.1093/jxb/erz349 PMC6812720

[nph17534-bib-0042] Suen DF , Tsai YH , Cheng YT , Radjacommare R , Ahirwar RN , Fu H , Schmidt W . 2018. The deubiquitinase OTU5 regulates root responses to phosphate starvation. Plant Physiology 176: 2441–2455. 29301952 10.1104/pp.17.01525 PMC5841733

[nph17534-bib-0043] Sun S , Gu M , Cao Y , Huang X , Zhang X , Ai P , Zhao J , Fan X , Xu G . 2012. A constitutive expressed phosphate transporter, OsPht1;1, modulates phosphate uptake and translocation in phosphate‐replete rice. Plant Physiology 159: 1571–1581. 22649273 10.1104/pp.112.196345 PMC3425197

[nph17534-bib-0044] Teng W , Deng Y , Chen XP , Xu XF , Chen RY , Lv Y , Zhao YY , Zhao XQ , He X , Li B *et al*. 2013. Characterization of root response to phosphorus supply from morphology to gene analysis in field‐grown wheat. Journal of Experimental Botany 64: 1403–1411. 23382547 10.1093/jxb/ert023 PMC3598426

[nph17534-bib-0045] Teng W , Zhao YY , Zhao XQ , He X , Ma WY , Deng Y , Chen XP , Tong YP . 2017. Genome‐wide identification, characterization, and expression analysis of PHT1 phosphate transporters in wheat. Frontiers in Plant Science 8: 543. 28443126 10.3389/fpls.2017.00543 PMC5386973

[nph17534-bib-0046] Tufan HA , Stefanato FL , McGrann GRD , MacCormack R , Boyd LA . 2011. The barley stripe mosaic virus system used for virus‐induced gene silencing in cereals differentially affects susceptibility to fungal pathogens in wheat. Journal of Plant Physiology 168: 990–994. 21315476 10.1016/j.jplph.2010.11.019

[nph17534-bib-0047] Wang F , Deng M , Xu J , Zhu X , Mao C . 2018a. Molecular mechanisms of phosphate transport and signaling in higher plants. Seminars in Cell & Developmental Biology 74: 114–122. 28648582 10.1016/j.semcdb.2017.06.013

[nph17534-bib-0048] Wang J , Sun J , Miao J , Guo J , Shi Z , He M , Chen Y , Zhao X , Li B , Han F *et al*. 2013. A phosphate starvation response regulator Ta‐PHR1 is involved in phosphate signaling and increases grain yield in wheat. Annals of Botany 111: 1139–1153. 23589634 10.1093/aob/mct080 PMC3662521

[nph17534-bib-0049] Wang ZQ , Zhou X , Dong L , Guo J , Chen Y , Zhang Y , Wu L , Xu M . 2018b. iTRAQ‐based analysis of the *Arabidopsis* proteome reveals insights into the potential mechanisms of anthocyanin accumulation regulation in response to phosphate deficiency. Journal of Proteomics 184: 39–53. 29920325 10.1016/j.jprot.2018.06.006

[nph17534-bib-0050] Wiśniewski JR , Zougman A , Nagaraj N , Mann M . 2009. Universal sample preparation method for proteome analysis. Nature Methods 6: 359–362. 19377485 10.1038/nmeth.1322

[nph17534-bib-0051] Xu L , Zhao H , Wan R , Liu Y , Xu Z , Tian W , Ruan W , Wang F , Deng M , Wang J *et al*. 2019. Identification of vacuolar phosphate efflux transporters in land plants. Nature Plants 5: 84–94. 30626920 10.1038/s41477-018-0334-3

[nph17534-bib-0052] Yan W , Chen GH , Yang LF , Gai JY , Zhu YL . 2014. Overexpression of the rice phosphate transporter gene *OsPT6* enhances tolerance to low phosphorus stress in vegetable soybean. Scientia Horticulturae 177: 71–76.

[nph17534-bib-0053] Yang J , Xie MY , Yang XL , Liu BH , Lin HH . 2019. Phosphoproteomic profiling reveals the importance of CK2, MAPKs and CDPKs in response to phosphate starvation in rice. Plant Cell Physiology 60: 2785–2796. 31424513 10.1093/pcp/pcz167

[nph17534-bib-0054] Yang T , Hao L , Yao S , Zhao Y , Lu W , Xiao K . 2016. TabHLH1, a bHLH‐type transcription factor gene in wheat, improves plant tolerance to Pi and N deprivation via regulation of nutrient transporter gene transcription and ROS homeostasis. Plant Physiology and Biochemistry 104: 99–113. 27107183 10.1016/j.plaphy.2016.03.023

[nph17534-bib-0055] Yang WT , Baek D , Yun DJ , Hwang WH , Park DS , Nam MH , Chung ES , Chung YS , Yi YB , Kim DH . 2014. Overexpression of OsMYB4P, an R2R3‐type MYB transcriptional activator, increases phosphate acquisition in rice. Plant Physiology and Biochemistry 80: 259–267. 24813725 10.1016/j.plaphy.2014.02.024

[nph17534-bib-0056] Yang WT , Baek D , Yun DJ , Lee KS , Hong SY , Bae KD , Chung YS , Kwon YS , Kim DH , Jung KH *et al*. 2018. Rice OsMYB5P improves plant phosphate acquisition by regulation of phosphate transporter. PLoS ONE 13: e0194628. 29566032 10.1371/journal.pone.0194628 PMC5864048

[nph17534-bib-0057] Zeng YJ , Ying J , Liu JZ , Sun JH , Li B , Xiao HS , Li ZS . 2002. Function analysis of a wheat phosphate transporter in yeast mutant. Acta Genetica Sinica 29: 1017–1020 (in Chinese with English abstract). 12645267

[nph17534-bib-0058] Zhang F , Chen X , Vitousek P . 2013. Chinese agriculture: an experiment for the world. Nature 497: 33–35. 23636381 10.1038/497033a

[nph17534-bib-0059] Zhang F , Wu XN , Zhou HM , Wang DF , Jiang TT , Sun YF , Cao Y , Pei WX , Sun SB , Xu GH . 2014. Overexpression of rice phosphate transporter gene *OsPT6* enhances phosphate uptake and accumulation in transgenic rice plants. Plant and Soil 384: 259–270.

[nph17534-bib-0060] Zhang Y , Hu L , Yu D , Xu K , Zhang J , Li X , Wang P , Chen G , Liu Z , Peng C *et al*. 2019. Integrative analysis of the wheat *PHT1* gene family reveals a novel member involved in arbuscular mycorrhizal phosphate transport and immunity. Cells 8: pii: E490. 10.3390/cells8050490 PMC6562588 31121904

[nph17534-bib-0061] Zhou B , Sanz‐Sáez Á , Elazab A , Shen T , Sánchez‐Bragado R , Bort J , Serret MD , Araus JL . 2014. Physiological traits contributed to the recent increase in yield potential of winter wheat from Henan Province, China. Journal of Integrative of Plant Biology 56: 492–504. 10.1111/jipb.12148 24373600

[nph17534-bib-0062] Zhou J , Jiao F , Wu Z , Li Y , Wang X , He X , Zhong W , Wu P . 2008. *OsPHR2* is involved in phosphate‐starvation signaling and excessive phosphate accumulation in shoots of plants. Plant Physiology 146: 1673–1686. 18263782 10.1104/pp.107.111443 PMC2287342

[nph17534-bib-0063] Zhu N , Cheng S , Liu X , Du H , Dai M , Zhou DX , Yang W , Zhao Y . 2015. The R2R3‐type MYB gene *OsMYB91* has a function in coordinating plant growth and salt stress tolerance in rice. Plant Science 236: 146–156. 26025528 10.1016/j.plantsci.2015.03.023

