# Supplementary material for: TaPHT1;9‐4B and its transcriptional regulator TaMYB4‐7D contribute to phosphate uptake and plant growth in bread wheat
Source: New Phytol. 2021 Jul 2;231(5):1968–83. doi: 10.1111/nph.17534 (PMC8489284; doi:10.1111/nph.17534)
Supplement: Supplementary file 1 — Dataset S1 Identification and analysis of phosphate deficiency responsive proteins (PDRPs) in bread wheat roots using iTRAQ‐based proteomics. Dataset S2 Quantification analysis of 11 Pi‐deficiency responsive proteins (PDRPs) using PRM analysis in bread wheat roots. Dataset S3 Mapping PDRPs to the genomic loci of Chinese Spring. Fig. S1 Examples illustrating homoeolog mapping of proteomic peptides in this work. Fig. S2 Phenotype and growth parameters of wheat seedlings cultured under Pi‐deficient conditions for 10 d. Fig. S3 Comparison of 11 PDRPs with respect to their expression changes induced by Pi deficiency revealed using iTRAQ or PRM approaches. Fig. S4 Nucleotide and deduced amino acid sequence of TaPHT1;9‐4B. Fig. S5 Transcript level of TaPHT1;9 in the root and shoot tissues of the wheat plants cultured under Pi‐sufficient or ‐deficient media for 8 d. Fig. S6 The coding sequences of TaPHT1;9 homoeologs (4A, 4B and 4D) in Chinese Spring. Fig. S7 Functional analysis of TaPHT1;9 in bread wheat using BSMV‐VIGS. Fig. S8 Evaluation of the transcript levels of three TaPHT1 genes and TaIPS1.1 in the roots of the wheat plants infected by BSMV‐GFP or BSMV‐TaPHT1;9. Fig. S9 Molecular identification of transgenic rice lines expressing TaPHT1;9‐4B. Fig. S10 The effects of nucleotide mutations in three CRISPR mutants on TaPHT1;9‐4B protein. Fig. S11 Phenotypes, dry weights and P concentrations of three CRISPR mutants and WT Fielder control cultured under Pi‐sufficient conditions. Fig. S12 Y1H screening using TaPHT1;9‐4B promoter as bait. Fig. S13 Sequences and phylogenetic tree of TaMYB4‐7D. Fig. S14 Chromosomal location, subcellular localisation and transcriptional activation activities of TaMYB4‐7D. Fig. S15 Sequence of TaPHT1;9‐4B promoter. Fig. S16 Transcript level of TaMYB4 in the root and shoot tissues of the wheat plants cultured under Pi‐sufficient or ‐deficient media for 8 d. Fig. S17 Analysis of the bread wheat plants with TaMYB4 expression silenced by BSMV‐VIGS. [file NPH-231--s001.zip › nph17534-sup-0004-FigsS1-S24.pdf]

New Phytologist Supporting Information

Article title: TaPHT1;9-4B and its transcriptional regulator TaMYB4-7D contribute to phosphate uptake and plant growth in bread wheat

Authors: Pengfei Wang, Gezi Li, Guangwei Li, Shasha Yuan, Chenyang Wang, Yingxin Xie, Tiancai Guo, Guozhang Kang, Daowen Wang.

Article acceptance date: 25 May 2021

The following Supporting Information is available for this article:

Fig. S1 Examples illustrating homoeolog mapping of proteomic peptides in this work.

Fig. S2 Phenotype and growth parameters of wheat seedlings cultured under Pi deficient conditions for 10 d.

Fig. S3 Comparison of 11 PDRPs with respect to their expression changes induced by Pi deficiency revealed using iTRAQ or PRM approaches.

Fig. S4 Nucleotide and deduced amino acid sequence of *TaPHT1;9-4B*.

Fig. S5 Transcript level of *TaPHT1;9* in the root and shoot tissues of the wheat plants cultured under Pi sufficient or deficient media for 8 d.

Fig. S6 The coding sequences of *TaPHT1;9* homoeologs (4A, 4B, and 4D) in Chinese Spring.

Fig. S7 Functional analysis of *TaPHT1;9* in bread wheat using BSMV-VIGS.

Fig. S8 Evaluation of the transcript levels of three *TaPHT1* genes and *TaIPS1.1* in the roots of the wheat plants infected by BSMV-GFP or BSMV-TaPHT1;9.

Fig. S9 Molecular identification of transgenic rice lines expressing *TaPHT1;9-4B*.

Fig. S10 The effects of nucleotide mutations in three CRISPR mutants on TaPHT1;9-4B protein.

Fig. S11 Phenotypes, dry weights, and P concentrations of three CRISPR mutants and WT Fielder control cultured under Pi sufficient conditions.

Fig. S12 Y1H screening using *TaPHT1;9-4B* promoter as bait.

Fig. S13 Sequences and phylogenetic tree of TaMYB4-7D.

Fig. S14 Chromosomal location, subcellular localization, and transcriptional activation activities of TaMYB4-7D.

Fig. S15 Sequence of *TaPHT1;9-4B* promoter.

Fig. S16 Transcript level of *TaMYB4* in the root and shoot tissues of the wheat plants cultured under Pi sufficient or deficient media for 8 d.

Fig. S17 Analysis of the bread wheat plants with *TaMYB4* expression silenced by BSMV-VIGS.

Fig. S18 Evaluation of foliar anthocyanin contents in the wheat plants infected by BSMV-GFP or BSMV-TaMYB4.

Fig. S19 Evaluation of the transcript levels of four anthocyanin biosynthesis genes in the leaves of BSMV-GFP or BSMV-TaMYB4 infected wheat plants.

Fig. S20 Analysis of nucleotide diversity of *PHT1;9-4B* promoter and its genomic coding sequence in bread wheat and relatives.

Fig. S21 Phenotypes, dry weights, P contents, and P concentrations of the 16 wheat varieties with different promoter haplotypes of *TaPHT1;9-4B* under low Pi conditions.

Fig. S22 Nucleotide sequence comparison of the DNA fragments used to differentiate four *TaPHT1;9-4B* promoter haplotypes (*Hap1* to *Hap4*).

Fig. S23 Nucleotide sequence of the promoter region of *TaPHT1;3-5B*, *TaPHT1;6-5B*, and *TaPT2*.

Fig. S24 Binding of TaMYB4-7D to the promoter region of *TaPHT1;3-5B*, *TaPHT1;6-5B*, and *TaPT2*.

Methods S1 Additional description of methods (see separate file).

Table S1 Accessions of hexaploid bread wheat and its relative species used in this study (see separate file).

Table S2 All primers used in this study (see separate file).

Table S3 Accession numbers of PHT proteins for constructing phylogenetic tree of TaPHT1;9-4B (see separate file).

Table S4 Identities of PHT proteins used for constructing the phylogenetic tree of TaPHT1;9-4B (see separate file).

Table S5 Potential proteins interacting with the promoter of TaPHT1;9-4B obtained using Y1H screening (see separate file).

Dataset S1 Identification and analysis of phosphate deficiency responsive proteins (PDRPs) in bread wheat roots using iTRAQ based proteomics (see separate file).

Dataset S2 Quantification analysis of 11 Pi deficiency responsive proteins (PDRPs) using PRM analysis in bread wheat roots (see separate file).

Dataset S3 Mapping PDRPs to the genomic loci of Chinese Spring (see separate file).

(a)

```
TaFOMT-5A MAAEMTVPSDAQLIKAQADLQRHSLTYLTSMSLRCAIELGIPTAIHRLGGTASLPDLMAALSLPPPKEPFLSRVLRLLAKSDALACTEDG  90
TaFOMT-4B MAAHMTVPSDAQLIKAQADLQRHSLTYLTSMALRCAIELGIPTAIHRLGGTASLPDLMAALSLPPPKAPFLSRVLRLLAKSNALACTDAG  90
TaFOMT-4D MAAHMTVPSDAQLIKAQADLQRHSLTYLTSMALRCAIELGIPTAIHRLGGTASLADLMAALSLPPPKAPFLSRVLRLLAKSDALSCTEDG  90
                                                                *

TaFOMT-5A VYSLTPLSYILVDGVLIDGEARQIAFPLAVTSRYHMESGLGLADWFKNDRALPVPSPFEHVHAAAPFDESMTLLDPETDKLFYEALAAHD 180
TaFOMT-4B IYSLTPLSYILVDGVLIDGEARQTAFPLAVTSRYHMESGLGLADWFKNDRALPVPSPFEHVHAAAPFDESMTLLDHETDKLFYEGLAAHD 180
TaFOMT-4D VYSLTPLSYILVDGVLIDGEARQIAFPLAVTSRYHMESGLGLADWFKNDRALPVPSPFEHVHAAAPFDESMTLLDPETDKLFYEALAAHD 180
                                 *                                                   *

TaFOMT-5A HMGIGTVVRECRGLFNGLQSLTDCCGGDGTTARAIVKAFPHIKCNVLDLPKVIEKVPSDGVVNYVAGDMFHTVPPAQAVMLKI...FFSI 267
TaFOMT-4B HMGIGTVVRECRGLFNGLESLTDCCGGDGTTARAIVKAFPHIKCNVLDLPQVIEKVPSDGVVNYVAGDLFHTVPPAQAVMLKLVLHFWSD 270
TaFOMT-4D HMGIGTVVRECRGLFNGLESLTDCCGGDGTTARAIVKAFPHIKCNVLDLPKVIEKVPSDGVVNYVAGDLFHTVPPAQAVMLKLVLHFWSD 270
                                                                              *

TaFOMT-5A PACSTLLERRGLHQDPRSMQ.........EGHTFP................RNGRKSDHHRHSAWIFSRDNI.................. 314
TaFOMT-4B DDCIKILAQCKKAIPSREMGGKVIIIDIVLGSSLETITETELLMDMLMFICTRGRQRDEKEWST.IFMKAGFSDYKIVKKLGHRGVIEVY 359
TaFOMT-4D EDCIKILAQCKKAIPSREMGGKVIIIDIVLGSSLETITETELLMDMLMFICTRGRQRDEKEWST.IFTKAGFSNYKIVKKLGHRGVIEVY 359

TaFOMT-5A .                                                                                          314
TaFOMT-4B P                                                                                          360
TaFOMT-4D P                                                                                          360
```

(b)

```
TaAPX2-1A MMRTVAVVAVAVLLAAAVAAEAGELKVGYYDKSCRGVENVVKWHVARAIKANRKSGAALVRLIFHDCFVRGCDASVLLDPTPENPKTEKT  90
TaAPX2-1B MMRTVAAVAVAVLLAAAVAAEASELKVGYYDKSCRGVENVVKWHVARAIKANRKSGAALVRLIFHDCFVRGCDASVLLDPTPENPDTEKT  90
TaAPX2-1D MMRTVAAVAVAVLLAAAVAAEASELKVGYYDKSCRGVENVVKWHVARAIKANRKSGAALVRLIFHDCFVRGCDASVLLDPTPENPHTEKT  90
                                                                                               *

TaAPX2-1A APINIGLAAFDLLDDIKAAVEDRCPGVVSCADILIFAARDAASLLSNGHVHFDALAGRLDGMHSHAAEAQQDLPDSTFTIAELIQNFKRK 180
TaAPX2-1B APINIGLAAFDLLDDIKSAVEDRCPGVVSCADILIFAARDAASLLSNGHVHFDALAGRLDGMHSHAAEAQQDLPDSTFTIAELIQNFKRK 180
TaAPX2-1D APINIGLAAFDLLDDIKAAVEDRCPGVVSCADILIFAARDAASLLSNGHVHFDALAGRLDGVHSHAAEAQQDLPDSTFTIAELIQNFKRK 180
                                                                       *

TaAPX2-1A NFTIEELVILSGAHAVGVGHCSSLRARLTAPADQILPAYRGLLAGKCAKGPDPIVPNNIRDEDAGAVAAAVPGFLPKLRKVKDFLDNSYY 270
TaAPX2-1B NFTIEELVILSGAHAVGVGHCSSLRARLTAPADQILPAYRGLLAGKCAKGPDPIVPNNIRDEDAGAVAAAIPGFLPKLRKVKDFLDNSYY 270
TaAPX2-1D NFTIEELVILSGAHAVGVGHCSSLRARLTAPADQILPAYRGLLAGKCAKGPDPIVPNNIRDEDAGAVAATIPGFLPKLRKVKDFLDNSYY 270
                                                                               **

TaAPX2-1A HNNLARIVTFNSDWQLLTEKEARGHVHEYADNGTLWDEDFSDSLVKLSKLPMPHGSKGEIRKMCRFVNH.                     339
TaAPX2-1B HNNLARIVTFNSDWQLLTEKEARGHVHEYADNGTLWDEDFSDSLVKLSKLPMPHGSKGEIRKMCRFVNHH                     340
TaAPX2-1D HNNLARIVTFNSDWQLLTEKEARGHVHEYADNGTLWDEDFSDSLVKLSKLPMPHGSKGEIRKMCRFVNHH                     340
```

(c)

```
TaCRT-2A  MAIRRGPSCAALALLALASVAAVSADVFFQEKFEDGWESRWVKSEWKKDENMAGEWNHTSGKWHGDAEDKGIQTSEDYRFYAISAEYPEF  90
TaCRT-2B  MAIRRGPSCAVLALLALASVAAVSADVFFQEKFEDGWESRWVKSEWKKDENMAGEWNHTSGKWHGDAEDKGIQTSEDYRFYAISAEYPEF  90
TaCRT-2D  MAIRRGSSCAVLALLALASVAAVSADVFFQEKFEDGWESRWVKSEWKKDENMAGEWNHTSGKWHGDAEDKGIQTSEDYRFYAISAEYPEF  90

TaCRT-2A  SNKDKTLVLQFTVKHEQKLDCGGGYVKLLGGDVDQKKFGGDTPYSIMFGPDICGYSTKKVHTILTKDGKNHLIKKDVPCETDQLSHVYTL 180
TaCRT-2B  SNKDKTLVLQFTVKHEQKLDCGGGYVKLLGGDVDQKKFGGDTPYSIMFGPDICGYSTKKVHTILTKDGKNHLIKKDVPCETDQLSHVYTL 180
TaCRT-2D  SNKDKTLVLQFTVKHEQKLDCGGGYVKLLGGDVDQKKFGGDTPYSIMFGPDICGYSTKKVHTILTKDGKNHLIKKDVPCETDQLSHVYTL 180

TaCRT-2A  IIRPDATYSILIDNEEKQTGSIYEHWDILPPKEIKDPEAKKPVDWDDKEYIPDPEDVKPEGYDDIPKEVTDPDAKKPEDWDDEEDGEWTA 270
TaCRT-2B  IIRPDATYSILIDNEEKQTGSIYEHWDILPPKEIKDPEAKKPEDWDDKEYIPDPEDVKPEGYDDIPREVTDPDAKKPEDWDDEEDGEWTA 270
TaCRT-2D  IIRPDATYSILIDNEEKQTGSIYEHWDILPPKEIKDPEAKKPEDWDDKEYIPDPEDVKPEGYDDIPKEVTDPDAKKPEDWDDEEDGEWTA 270
                                                                            *

TaCRT-2A  PTIPNPEYKGPWKQKKIKNPNYQGKWKAPMIANPDFKDDPYIYAFDSLKYIGIELWQVKSGTLFDNILITDDAALAKTFAEETWAKHKEA 360
TaCRT-2B  PTIPNPEYKGPWKQKKIKNPNYQGKWKAPMIANPDFKDDPYIYAFNSLKYIGIELWQVKSGTLFDNILITDDAALAKTFAEETWAKHKEA 360
TaCRT-2D  PTIPNPEYKGPWKQKKIKNPNYQGKWKAPMIANPDFKDDPYIYAFDSLKYIGIELWQVKSGTLFDNILITDDAALAKTFAEETWAKHKEA 360
                                                       *

TaCRT-2A  EKAAFDAAEKKKEEEDASKAGEDDDDLDDEDADDEDKDDKAGSDVESDDEKHDEL                                    415
TaCRT-2B  EKAAFDAAEKKKEEEDASKAGEDDDDLDDEEADDEDKDDKAGSDAESDDEKHDEL                                    415
TaCRT-2D  EKAAFDAAEKKKEEEDASKAGEDDDDLDDEEADDEDKDDKAGSDAESDDEKHDEL                                    415
```

Specific to A; **Specific to B**; **Specific to D**; **Indistinguishable**

Fig. S1 Examples illustrating homoeolog mapping of proteomic peptides in this work. (a) Homoeolog mapping of the five proteomic peptides obtained for a wheat flavonoid *O*-methyltransferase-like protein (FOMT, NCBI accession no. XP_020149867.1). Based on the peptide mapping result, this protein was expressed from all three subgenome homoeologs (*TraesCS5A02G521000*, *TraesCS4B02G352200*, and *TraesCS4D02G346800*). (b) Homoeolog mapping of the five proteomic peptides obtained for a wheat peroxidase 2 protein (APX2, NCBI accession no. EMS53366.1). Judging the peptide mapping data, this protein was expressed from the A (*TraesCS1A02G0777*) and D (*TraesCS1D02G079800*) subgenome homoeologs. (c) Homoeolog mapping of the two proteomic peptides obtained for a wheat calreticulin-like protein (CRT, NCBI accession no. AAW02798.1). According to the peptide mapping result, this protein was expressed from only the B subgenome homoeolog (*TraesCS2B02G576100*).

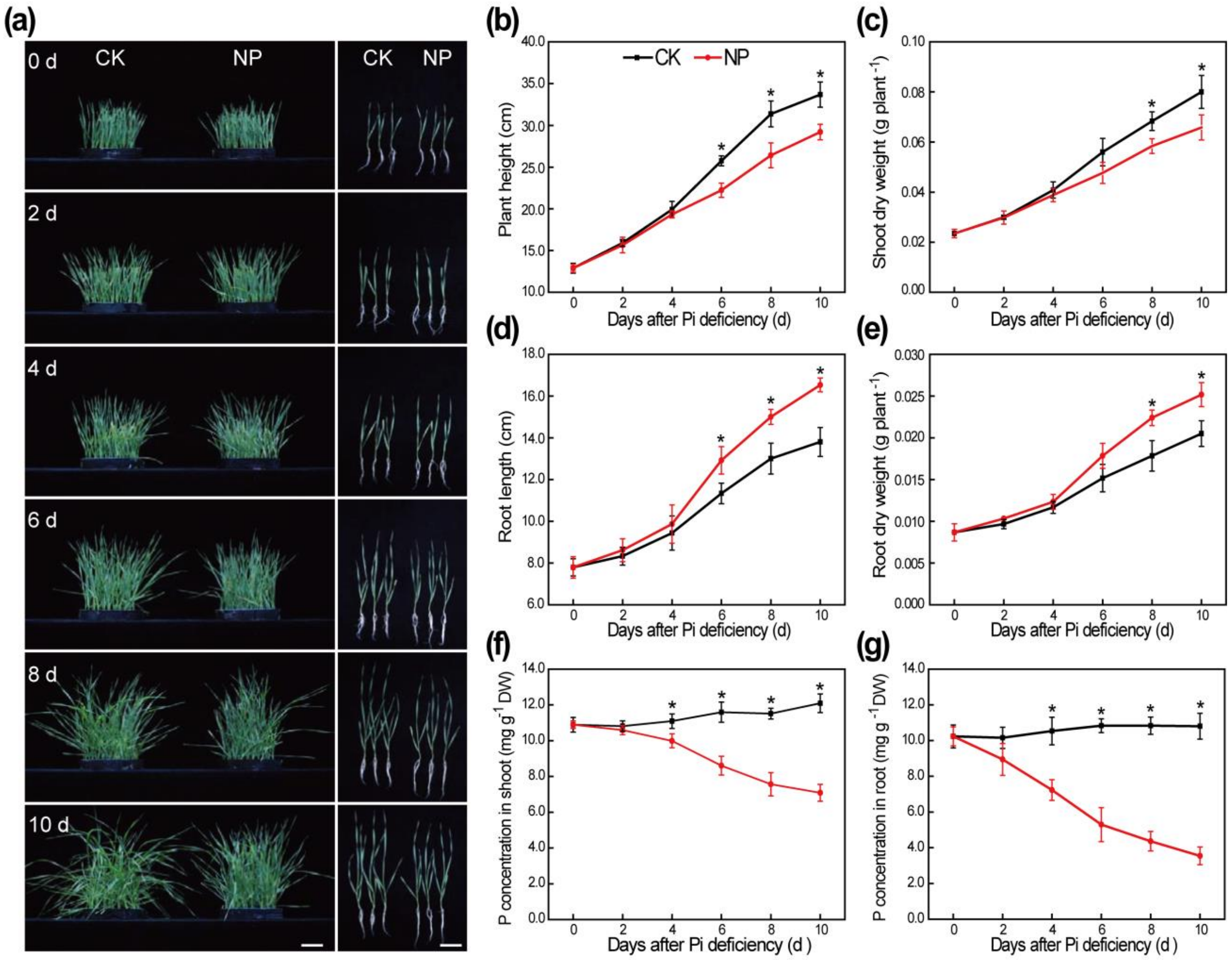

Fig. S2 Phenotype and growth parameters of wheat seedlings cultured under Pi deficient conditions for 10 d. Two-week-old bread wheat seedlings were transferred to Pi sufficient (1 mM, CK) or deficient (0 mM, NP) Hoagland media and cultured for 10 d, with their morphology (a), plant height (b), shoot dry weight (c), root length (d), root dry weight (e), shoot P concentration (f), and root P concentration (g) measured at 2-d interval. Bars, 5 cm. Fresh plants were separated into shoots (leaf and sheath) and roots. Data represent means ± SD of three biological replicates with at least six plants per replicate. Asterisks indicate statistically significant differences ($P < 0.05$, Student's *t*-test).

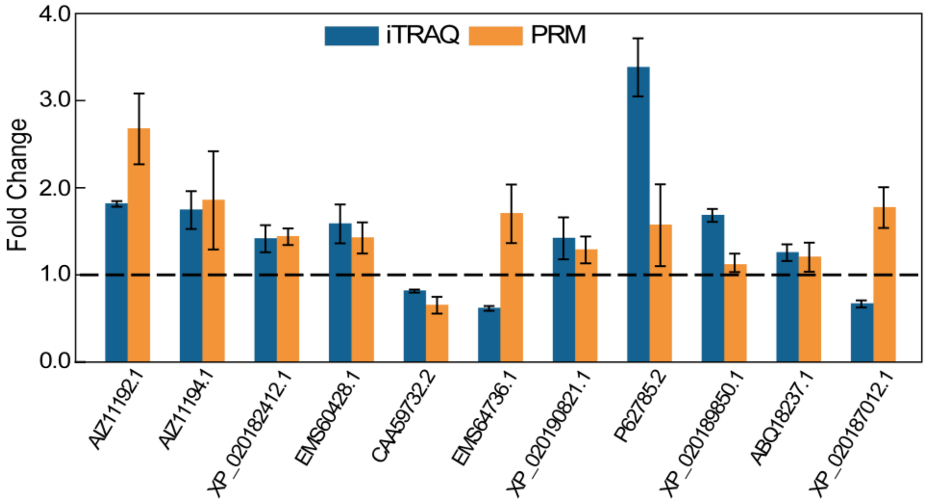

Fig. S3 Comparison of 11 PDRPs with respect to their expression changes induced by Pi deficiency revealed using iTRAQ or PRM approaches. Each fold change value was the mean ± SD of three biological replicates. AIZ11192.1, TaPHT1;9-4B; AIZ11194.1, TaPT2; XP_020182412.1, GDSL esterase; EMS60428.1, calreticulin; CAA59732.2, putative zinc finger protein; EMS64736.1, germin-like protein 8-14; XP_020190821.1, phospho-2-dehydro-3-deoxyheptonate aldolase 2; P62785.2, histone H4; XP_020189850.1, probable leucine-rich repeat receptor-like protein kinase; ABQ18237.1, translationally-controlled tumor protein; XP_020187012.1: long chain acyl-CoA synthetase 4-like. Further details are provided in Dataset S2.

```
1     ATGGCGACTGAACAGCTCAACGTGTTGAAAGCACTGGACGTTGCCAAGACGCAGTTGTACCATTTCAAGGCGGTCGTGATCGCCGGCATG
1      M  A  T  E  Q  L  N  V  L  K  A  L  D  V  A  K  T  Q  L  Y  H  F  K  A  V  V  I  A  G  M
91    GGCTTCTTCACGGACGCCTACGACCTCTTCTGCATCGCCCTCGTCACCAAGCTGCTGGGGCGCATCTACTACACCGACCCTGCCCTCAAC
31     G  F  F  T  D  A  Y  D  L  F  C  I  A  L  V  T  K  L  L  G  R  I  Y  Y  T  D  P  A  L  N
181   GAGCCCGGCCACCTCCCGGCAAATGTGTCGGCCGCCGTGAACGGCGTGGCCCTGTGTGGCACACTTGCCGGCCAGCTCTTCTTCGGCTGG
61     E  P  G  H  L  P  A  N  V  S  A  A  V  N  G  V  A  L  C  G  T  L  A  G  Q  L  F  F  G  W
271   CTCGGTGACAAGCTCGGCCGCAAGAGCGTCTACGGCTTCACGCTCATTCTCATGGTCCTCTGCTCCATCGCGTCTGGGCTCTCGTTTGGA
91     L  G  D  K  L  G  R  K  S  V  Y  G  F  T  L  I  L  M  V  L  C  S  I  A  S  G  L  S  F  G
361   CACGAGGCCAAGGGCGTAATGGGCACGCTATGTTTCTTCCGCTTCTGGCTCGGCTTCGGCGTCGGCGGTGACTACCCTCTGAGCGCCACA
121    H  E  A  K  G  V  M  G  T  L  C  F  F  R  F  W  L  G  F  G  V  G  G  D  Y  P  L  S  A  T
451   ATCATGTCGGAGTATGCTAACAAGAAGACCCGCGGCACCTTTATCGCCGCCGTGTTTGCCATGCAGGGGTTTGGCATCCTATTTGGTACT
151    I  M  S  E  Y  A  N  K  K  T  R  G  T  F  I  A  A  V  F  A  M  Q  G  F  G  I  L  F  G  T
541   ATCGTCACCATCATCGTCTCGTCTGCATTCCGACATGCATTCCCTGCACCGCCATTCTACATTGACGCCGCAGCGTCCATTGGCCCAGAG
181    I  V  T  I  I  V  S  S  A  F  R  H  A  F  P  A  P  P  F  Y  I  D  A  A  A  S  I  G  P  E
631   GCCGACTACGTGTGGCGCATCATCGTCATGTTCGGCACCATCCCGGCCGCCCTGACCTACTACTGGCGCATGAAGATGCCCGAAACTGCG
211    A  D  Y  V  W  R  I  I  V  M  F  G  T  I  P  A  A  L  T  Y  Y  W  R  M  K  M  P  E  T  A
721   CGGTACACAGCACTCATCGCCGGCAACACGAAGCAAGCCACATCAGACATGTCCAAGGTGCTCAACAAGGAGATCTCAGAGGAGGATGTG
241    R  Y  T  A  L  I  A  G  N  T  K  Q  A  T  S  D  M  S  K  V  L  N  K  E  I  S  E  E  D  V
811   CAGGGTGAGCGGGCCACTGGTGATACATGGGGCCTCTTCTCCCGACAGTTCATGAAGCGTCACGGGGTGCACTTGCTAGCGACCACAAGC
271    Q  G  E  R  A  T  G  D  T  W  G  L  F  S  R  Q  F  M  K  R  H  G  V  H  L  L  A  T  T  S
901   ACTTGGTTCCTGCTCGATGTGGCCTTCTATAGCCAGAACCTGTTCCAGAAGGACATCTTCACCAAGATCGGGTGGATCCCGCCAGCCAAG
301    T  W  F  L  L  D  V  A  F  Y  S  Q  N  L  F  Q  K  D  I  F  T  K  I  G  W  I  P  P  A  K
991   ACTATGAATGCATTGGAGGAGTTGTACCGCATCGCCCGCGCCCAAGCGCTCATCGCGCTCTGCGGCACCGTGCCCGGCTACTGGTTCACC
331    T  M  N  A  L  E  E  L  Y  R  I  A  R  A  Q  A  L  I  A  L  C  G  T  V  P  G  Y  W  F  T
1081  GTCGCCTTCATCGACATCATTGGGAGGTTTTGGATCCAGCTTATGGGATTCACCATGATGACCATTTTCATGCTCGCAATCGCCATACCT
361    V  A  F  I  D  I  I  G  R  F  W  I  Q  L  M  G  F  T  M  M  T  I  F  M  L  A  I  A  I  P
1171  TACGACTACTTGGTGAAGCCAGGGCACCACACCGGCTTCGTCGTGCTCTACGGGCTCACTTTCTTCTTCGCCAACTTCGGGCCCAACAGC
391    Y  D  Y  L  V  K  P  G  H  H  T  G  F  V  V  L  Y  G  L  T  F  F  F  A  N  F  G  P  N  S
1261  ACGACCTTCATTGTGCCAGCCGAGATTTTCCCTGCGAGGCTCCGATCCACATGTCATGGTATCTCTGCCGCTACCGGTAAGGCAGGCGCG
421    T  T  F  I  V  P  A  E  I  F  P  A  R  L  R  S  T  C  H  G  I  S  A  A  T  G  K  A  G  A
1351  ATCATTGGCGCGTTCGGGTTCCTGTATGCGTCCCAGGACCAGAAGAAGCCCGAGACCGGTTACTCACGAGGAATCGGCATGCGCAACGCA
451    I  I  G  A  F  G  F  L  Y  A  S  Q  D  Q  K  K  P  E  T  G  Y  S  R  G  I  G  M  R  N  A
1441  CTCTTCGTGCTCGCAGGCACAAATTTCCTGGGCCTGCTCTTTTCCCTGCTGGTGCCGGAGTCCAAGGGCAAGTCACTGGAGGAGCTCTCC
481    L  F  V  L  A  G  T  N  F  L  G  L  L  F  S  L  L  V  P  E  S  K  G  K  S  L  E  E  L  S
1531  AAGGAGAACGTCGGCGACGATGGCATCGAAGCTTAG
511    K  E  N  V  G  D  D  G  I  E  A  *
```

Fig. S4 Nucleotide and deduced amino acid sequences of *TaPHT1;9-4B*.

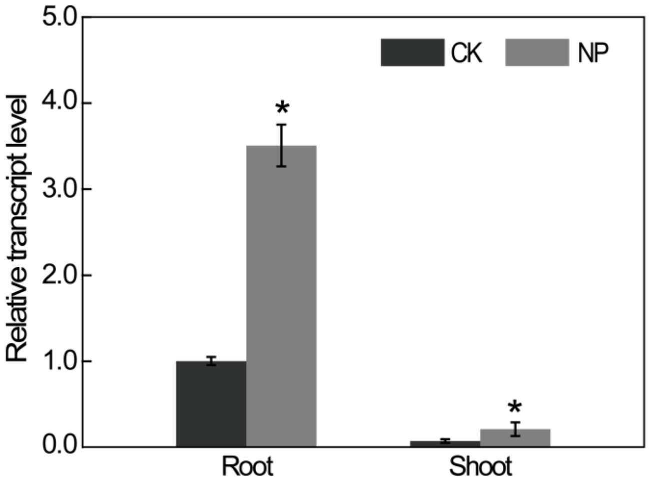

Fig. S5 Transcript level of *TaPHT1;9* in the root and shoot tissues of the wheat plants cultured under Pi sufficient or deficient media for 8 d. Two-week-old wheat seedlings were transferred to Pi sufficient (CK, 1 mM Pi) or deficient (NP, 0 mM Pi) Hoagland media for 8 d. Transcript levels were normalized to the internal control of *TaActin*. The PCR primers for amplifying *TaPHT1;9* were gene specific but recognized all three homoeologs of *TaPHT1;9* (Table S2). Data represent means ± SD of three biological replicates, and asterisks indicate statistically significant differences ($P < 0.05$, Student's *t*-test).

```
TaPHT1;9-4A-CDS ATGGCGACTGAACAGCTCAACGTGTTGAAAGCACTGGACGTTGCCAAGACGCAACTGTACCATTTCAAGGCCGTCGTGATCGCCGGCATGGGCTTCTTCA 100
TaPHT1;9-4B-CDS ATGGCGACTGAACAGCTCAACGTGTTGAAAGCACTGGACGTTGCCAAGACGCAGTTGTACCATTTCAAGGCGGTCGTGATCGCCGGCATGGGCTTCTTCA 100
TaPHT1;9-4D-CDS ATGGCGACTGAACAGCTCAACGTGTTGAAAGCACTCGATGTTGCCAAGACGCAACTGTACCATTTCAAGGCGGTCGTGATCGCCGGCATGGGCTTCTTCA 100

TaPHT1;9-4A-CDS CGGACGCCTACGACCTCTTCTGCATCGCCCTCGTCACCAAGCTGCTGGGACGCATCTACTACACCGACCCTGCCCTCAATGAGCCCGGCCACCTCCCGGC 200
TaPHT1;9-4B-CDS CGGACGCCTACGACCTCTTCTGCATCGCCCTCGTCACCAAGCTGCTGGGGCGCATCTACTACACCGACCCTGCCCTCAACGAGCCCGGCCACCTCCCGGC 200
TaPHT1;9-4D-CDS CGGACGCCTACGACCTCTTCTGCATCGCCCTCGTCACCAAGCTGCTGGGGCGCATCTACTACACCGACCCTGCCCTCAACGAGCCCGGCCACCTCCCGGC 200

TaPHT1;9-4A-CDS AAACGTGTCAGCCGCCGTGAACGGCGTGGCCCTATGCGGCACACTTGCCGGCCAGCTCTTCTTCGGCTGGCTCGGTGACAAGCTCGGCCGCAAGAGCGTC 300
TaPHT1;9-4B-CDS AAATGTGTCGGCCGCCGTGAACGGCGTGGCCCTGTGTGGCACACTTGCCGGCCAGCTCTTCTTCGGCTGGCTCGGTGACAAGCTCGGCCGCAAGAGCGTC 300
TaPHT1;9-4D-CDS AAACGTGTCGGCCGCCGTGAACGGCGTGGCCCTATGCGGCACACTTGCCGGCCAGCTCTTCTTCGGCTGGCTCGGTGACAAGCTCGGCCGCAAGAGCGTC 300

TaPHT1;9-4A-CDS TACGGCTTCACGCTCATCCTCATGGTCCTCTGCTCCATCGCGTCCGGGCTATCGTTTGGACACGAGGCCAAGGGTGTAATGGGGACGCTATGTTTCTTCC 400
TaPHT1;9-4B-CDS TACGGCTTCACGCTCATTCTCATGGTCCTCTGCTCCATCGCGTCTGGGCTCTCGTTTGGACACGAGGCCAAGGGCGTAATGGGCACGCTATGTTTCTTCC 400
TaPHT1;9-4D-CDS TACGGCTTCACGCTCATCCTCATGGTCCTCTGCTCCATCGCGTCCGGGCTCTCGTTTGGACACGAGGCCAAGGGCGTAATGGGGACGCTATGTTTCTTCC 400

TaPHT1;9-4A-CDS GTTTCTGGCTCGGCTTCGGTGTCGGCGGCGACTATCCTCTCAGCGCCACCATCATGTCGGAGTATGCTAACAAGAAGACCCGCGGCACCTTTATCGCCGC 500
TaPHT1;9-4B-CDS GCTTCTGGCTCGGCTTCGGCGTCGGCGGTGACTACCCTCTGAGCGCCACAATCATGTCGGAGTATGCTAACAAGAAGACCCGCGGCACCTTTATCGCCGC 500
TaPHT1;9-4D-CDS GCTTCTGGCTTGGCTTCGGCGTCGGCGGCGACTATCCTCTGAGCGCCACCATCATGTCGGAATATGCTAACAAGAAGACCCGCGGCACCTTTATCGCCGC 500

TaPHT1;9-4A-CDS CGTGTTTGCCATGCAGGGGTTTGGCATCCTATTTGGTACTATCGTCACGATCATCGTCTCGTCCGCATTCCGACATGCATTCCCTGCACCGCCATTCTAC 600
TaPHT1;9-4B-CDS CGTGTTTGCCATGCAGGGGTTTGGCATCCTATTTGGTACTATCGTCACCATCATCGTCTCGTCTGCATTCCGACATGCATTCCCTGCACCGCCATTCTAC 600
TaPHT1;9-4D-CDS TGTGTTTGCCATGCAGGGGTTTGGCATCCTATTTGGTACCATTGTCACGATCATCGTCTCGTCCGCATTCCGACATGCATTCCCTGCACCGCCATTCTAC 600

TaPHT1;9-4A-CDS ATCGACGCCGCGGCATCCATTGGCCCGGAGGCCGACTATGTGTGGCGCATCATCGTCATGTTCGGCACCATCCCGGCTGCCCTGACCTACTACTGGCGCA 700
TaPHT1;9-4B-CDS ATTGACGCCGCAGCGTCCATTGGCCCAGAGGCCGACTACGTGTGGCGCATCATCGTCATGTTCGGCACCATCCCGGCCGCCCTGACCTACTACTGGCGCA 700
TaPHT1;9-4D-CDS ATTGACGCCGCGGCGTCCATTGGCCCGGAGGCCGACTACGTGTGGCGCATCATCGTCATGTTCGGCACCATCCCGGCCGCCCTGACCTACTACTGGCGCA 700

TaPHT1;9-4A-CDS TGAAGATGCCCGAAACTGCACGGTACACGGCACTCATCGCCGGCAACACAAAGCAAGCCACATCAGACATGTCCAAGGTGCTCAACAAGGAGATCTCAGA 800
TaPHT1;9-4B-CDS TGAAGATGCCCGAAACTGCGCGGTACACAGCACTCATCGCCGGCAACACGAAGCAAGCCACATCAGACATGTCCAAGGTGCTCAACAAGGAGATCTCAGA 800
TaPHT1;9-4D-CDS TGAAGATGCCCGAAACTGCGCGGTACACAGCACTCATCGCCGGCAACACGAAGCAAGCCACATCAGACATGTCCAAGGTGCTCAACAAGGAGATCTCAGA 800

TaPHT1;9-4A-CDS AGAGAACGTCCAGGGTGAGCGGGCCACCGGTGATACCTGGGGCCTCTTCTCCCGACAGTTCATGAAGCGCCACGGGGTGCACTTGCTAGCGACCACAAGC 900
TaPHT1;9-4B-CDS GGAGGATGTGCAGGGTGAGCGGGCCACTGGTGATACATGGGGCCTCTTCTCCCGACAGTTCATGAAGCGTCACGGGGTGCACTTGCTAGCGACCACAAGC 900
TaPHT1;9-4D-CDS GGAGAATGTGCAGGGTGAGCGTGCCACTGGTGATACTTGGGGCCTCTTCTCGCGACAGTTCATGAAGCGCCACGGGGTGCACTTGCTAGCGACCACAAGC 900

TaPHT1;9-4A-CDS ACTTGGTTCCTACTCGATGTGGCCTTCTACAGCCAGAACCTGTTCCAGAAGGACATCTTCACCAAGATCGGGTGGATCCCGCCGGCCAAGACTATGAATG 1000
TaPHT1;9-4B-CDS ACTTGGTTCCTGCTCGATGTGGCCTTCTATAGCCAGAACCTGTTCCAGAAGGACATCTTCACCAAGATCGGGTGGATCCCGCCAGCCAAGACTATGAATG 1000
TaPHT1;9-4D-CDS ACTTGGTTCCTGCTCGATGTGGCCTTCTACAGCCAGAACCTGTTCCAAAAGGACATCTTCACCAAGATCGGGTGGATCCCGCCGGCCAAGACTATGAATG 1000

TaPHT1;9-4A-CDS CATTGGAGGAGTTGTACCGCATCGCCCGCGCCCAAGCGCTCATCGCGCTCTGTGGCACCGTGCCTGGCTACTGGTTCACCGTCGCCTTCATCGACATCAT 1100
TaPHT1;9-4B-CDS CATTGGAGGAGTTGTACCGCATCGCCCGCGCCCAAGCGCTCATCGCGCTCTGCGGCACCGTGCCCGGCTACTGGTTCACCGTCGCCTTCATCGACATCAT 1100
TaPHT1;9-4D-CDS CATTGGAGGAGTTGTACCGCATCGCCCGCGCCCAAGCGCTCATCGCGCTCTGCGGCACCGTGCCCGGCTACTGGTTCACCGTCGCCTTCATCGACATTAT 1100

TaPHT1;9-4A-CDS TGGGAGGTTTTGGATCCAGCTCATGGGATTCACCATGATGACCATTTTCATGCTAGCAATTGCCATACCGTACGACTACTTGGTGAAGCCAGGGCACCAC 1200
TaPHT1;9-4B-CDS TGGGAGGTTTTGGATCCAGCTTATGGGATTCACCATGATGACCATTTTCATGCTCGCAATCGCCATACCTTACGACTACTTGGTGAAGCCAGGGCACCAC 1200
TaPHT1;9-4D-CDS TGGGAGGTTTTGGATCCAGCTCATGGGATTCACCATGATGACCATTTTCATGCTCGCAATCGCCATACCTTACGACTACTTGGTGAAGCCAGGGCACCAC 1200

TaPHT1;9-4A-CDS ACCGGTTTCGTCGTGCTCTACGGGCTCACTTTCTTCTTCGCCAACTTCGGTCCCAACAGCACAACTTTCATCGTGCCAGCTGAGATCTTCCCTGCGAGGC 1300
TaPHT1;9-4B-CDS ACCGGCTTCGTCGTGCTCTACGGGCTCACTTTCTTCTTCGCCAACTTCGGGCCCAACAGCACGACCTTCATTGTGCCAGCCGAGATTTTCCCTGCGAGGC 1300
TaPHT1;9-4D-CDS ACCGGCTTCGTCGTGCTCTACGGGCTCACTTTCTTCTTCGCCAACTTCGGCCCCAACAGCACAACCTTCATTGTGCCAGCCGAGATCTTCCCTGCGAGGC 1300

TaPHT1;9-4A-CDS TCCGGTCCACATGCCACGGTATCTCTGCCGCTACTGGTAAGGCGGGCGCGATCATCGGCGCGTTCGGGTTCCTGTATGCATCGCAGGACCAGAAGAAGCC 1400
TaPHT1;9-4B-CDS TCCGATCCACATGTCATGGTATCTCTGCCGCTACCGGTAAGGCAGGCGCGATCATTGGCGCGTTCGGGTTCCTGTATGCGTCCCAGGACCAGAAGAAGCC 1400
TaPHT1;9-4D-CDS TCCGGTCCACATGCCACGGTATCTCTGCTGCTACCGGTAAGGCGGGCGCGATCATCGGCGCGTTCGGGTTCCTGTATGCGTCGCAGGACCAGAAGAAGCC 1400

TaPHT1;9-4A-CDS CGACACCGGCTACTCACGGGGAATTGGCATGCGCAACTCGCTCTTCGTGCTCGCAGGCACAAACTTCCTGGGCCTGCTCTTTTCCCTGCTGGTGCCGGAG 1500
TaPHT1;9-4B-CDS CGAGACCGGTTACTCACGAGGAATCGGCATGCGCAACGCACTCTTCGTGCTCGCAGGCACAAATTTCCTGGGCCTGCTCTTTTCCCTGCTGGTGCCGGAG 1500
TaPHT1;9-4D-CDS CGAGACCGGCTACTCACGGGGAATCGGCATGCGCAACGCACTCTTCGTGCTCGCAGGCACAAATTTCCTGGGCCTGCTCTTTTCCCTGCTGGTGCCGGAG 1500

TaPHT1;9-4A-CDS TCCAAGGGCAAGTCGCTCGAGGAGCTCTCCAAGGAGAACGTCGGCGACGATGGCATCGAAGCTTAG.................................. 1566
TaPHT1;9-4B-CDS TCCAAGGGCAAGTCACTGGAGGAGCTCTCCAAGGAGAACGTCGGCGACGATGGCATCGAAGCTTAG.................................. 1566
TaPHT1;9-4D-CDS TCCAAGGGCAAGTCGCTGGAGGAGCTCTCCAAGGAGAACGTCGGCGACGATGGCATCGAAGCTTAG.................................. 1566
```

Fig. S6 The coding sequences of *TaPHT1;9* homoeologs (4A, 4B, and 4D) in Chinese Spring. These sequences were retrieved from the IWGSC database (http://www.wheatgenome.org/). The fragment used to construct the VIGS vector (BSMV-TaPHT1;9) is underlined.

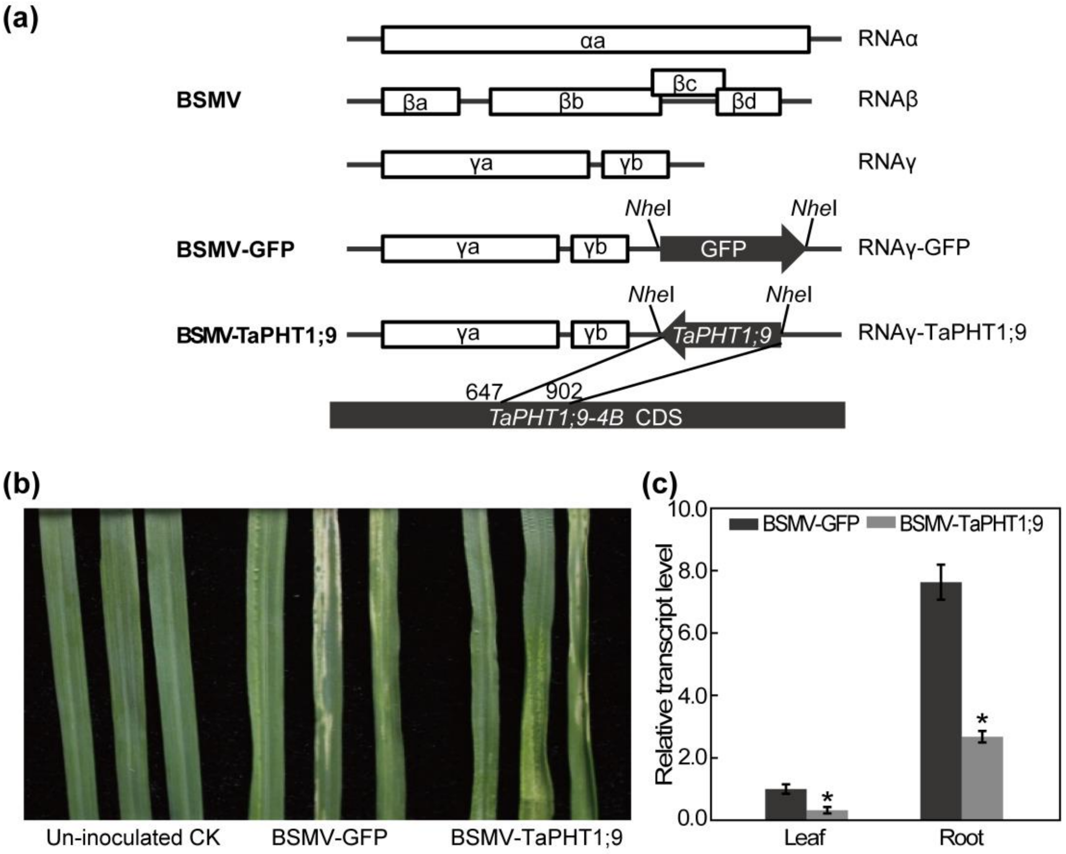

Fig. S7 Functional analysis of *TaPHT1;9* in bread wheat using BSMV-VIGS. (a) Diagrams of the genomic RNAs (RNAα, RNAβ, and RNAγ) of wild type BSMV and the modified versions of RNAγ (RNAγ-GFP and RNAγ-TaPHT1;9) in recombinant BSMV-GFP and BSMV-TaPHT1;9. The orientations of *GFP* or *TaPHT1:9* derived inserts in RNAγ-GFP or RNAγ-TaPHT1;9 are indicated by dark boxes. (b) Comparison of the fourth leaves of the wheat plants infected by BSMV-GFP or BSMV-TaPHT1;9 to those of un-inoculated controls (CK). Chlorosis and mild mosaic symptoms were observed on the leaves infected by BSMV-GFP or BSMV-TaPHT1;9. (c) Relative transcript levels of *TaPHT1;9* in the leaf and root tissues of the wheat plants infected by BSMV-GFP or BSMV-TaPHT1;9 at 7 d after virus inoculation as assessed by qRT-PCR. The infected plants were cultured in the Pi sufficient (1 mM) Hoagland media, and the transcript level of *TaPHT1;9* in the leaf tissues infected by BSMV-GFP was set as 1 to facilitate cross comparison among the four treatments. Amplification of *TaActin* served as an internal control. *TaPHT1;9* expression was significantly decreased in wheat leaves and roots by BSMV-VIGS. Data represent means ± SD of three biological replicates, and asterisks indicate statistically significant differences ($P < 0.05$, Student's t-test). The primers used in qRT-PCR assay are listed in Table S2.

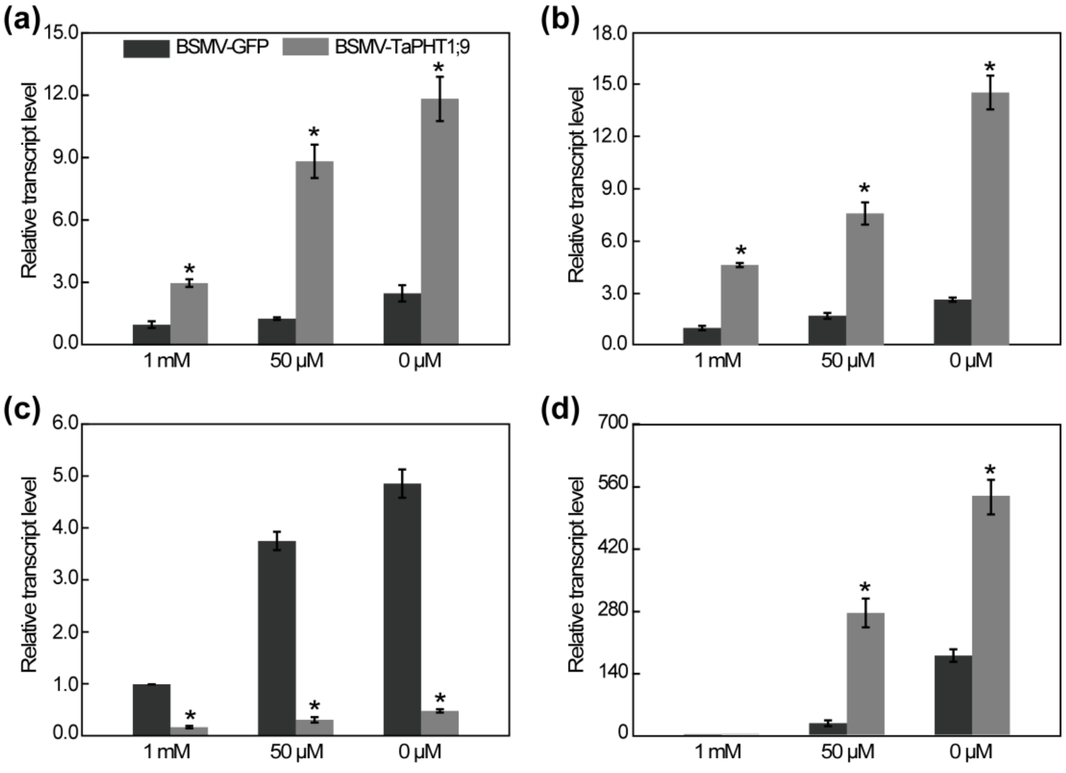

Fig. S8 Evaluation of the transcript levels of three *TaPHT1* genes and *TaIPS1.1* in the roots of the wheat plants infected by BSMV-GFP or BSMV-TaPHT1;9. (a) *TaPHT1;3*. (b) *TaPHT1;6*. (c) *TaPHT1;*9. (d) *TaIPS1.1*. The plants infected by BSMV-GFP (control) or BSMV-TaPHT1;9 (silencing *TaPHT1;9*) were cultured under Pi sufficient (1 mM), low Pi (50 μM) or Pi deficient (0 μM) Hoagland solutions for 10 d before being used for qRT-PCR assays with gene specific primers (Table S2). Transcript levels were normalized to the internal control of *TaActin*. Data represent means ± SD of three biological replicates, and asterisks indicate statistically significant differences ($P < 0.05$, Student's *t*-test).

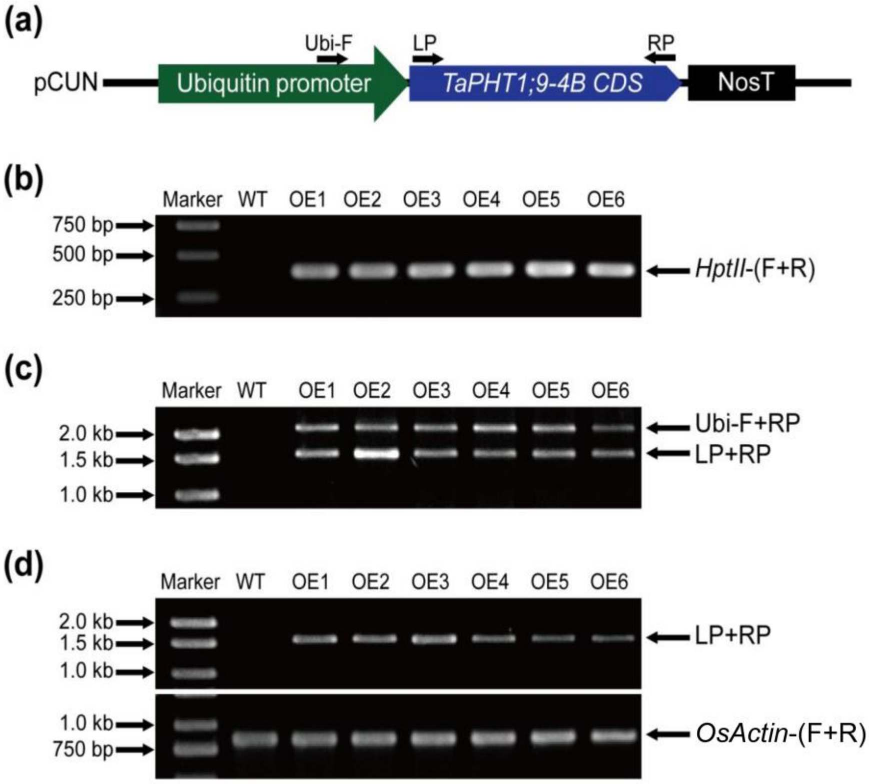

Fig. S9 Molecular identification of transgenic rice lines expressing *TaPHT1;9-4B*. (a) Organization of *TaPHT1;9-4B* overexpression cassette in the pCUN construct used for rice transformation. This cassette was under the control of maize ubiquitin gene promoter (Ubi), with the transcription terminator derived from nopaline synthase gene (NosT). LP, RP, and Ubi-F represent primers used for identifying the rice transformants carrying *TaPHT1;9-4B* expression cassette. (b) Identification of *TaPHT1;9-4B* transgenic lines by PCR amplification of the selection marker gene *Hpt II*. A 409 bp fragment (arrowed) was amplified using the *HptII*-(F+R) primers from the genomic DNA of positive transgenic rice lines (OE1 - OE6). This fragment was not amplified from the untransformed wild type (WT) control. (c) Verification of the presence of *TaPHT1;9-4B* expression cassette in the positive transgenic lines but not WT control. As anticipated, two fragments (2,138 bp and 1,566 bp) were amplified from only the positive lines (OE1 - OE6) using the primer sets Ubi-F+RP and LP+RP, respectively. (d) Detection of *TaPHT1;9-4B* transcripts in the root tissues of positive transgenic lines (OE1 - OE6) by semi-quantitative RT-PCR analysis. A 1566 bp fragment was amplified using LP+RP primers from only the root cDNAs of the positive transgenic lines expressing *TaPHT1;9-4B*. *OsActin* gene (NCBI accession no. AB047313) was used as internal control, with a 826 bp fragment amplified using *OsActin*-(F+R) primers. All primer sequences are listed in Table S2. Two independent *TaPHT1;9-4B* transgenic lines (OE1 and OE3) were selected for further experiments.

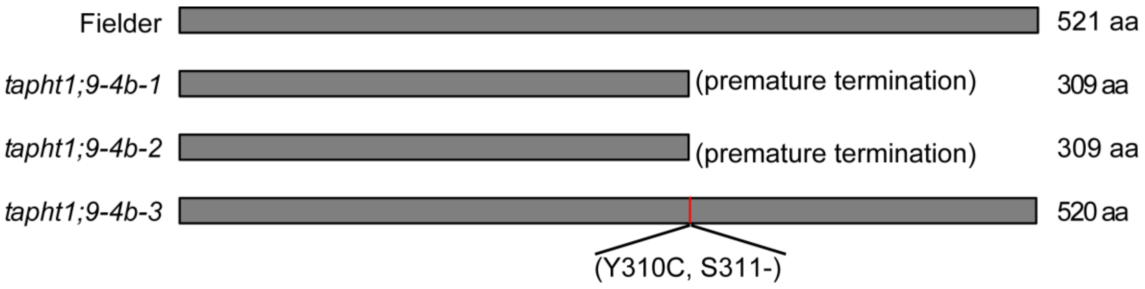

Fig. S10 The effects of nucleotide mutations in three CRISPR mutants on TaPHT1;9-4B protein.

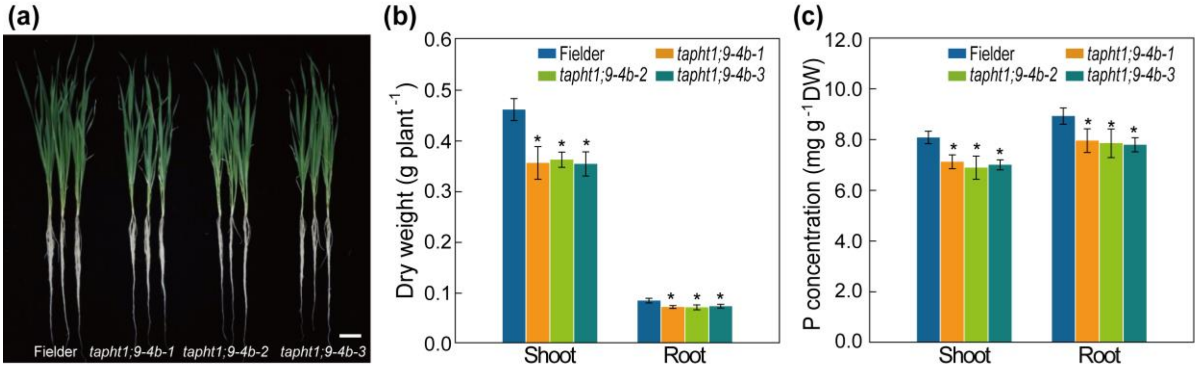

Fig. S11 Phenotypes (a), dry weights (b), and P concentrations (c) of three CRISPR mutants and WT Fielder control cultured under Pi sufficient conditions. Bars, 5 cm. Two-week-old seedlings were transferred to the Pi sufficient (1 mM) medium, and cultured for 21 days before being used for the analysis. Data represent means ± SD of three biological replicates. Asterisks indicate statistically significant differences ($P < 0.05$, Student's *t*-test).

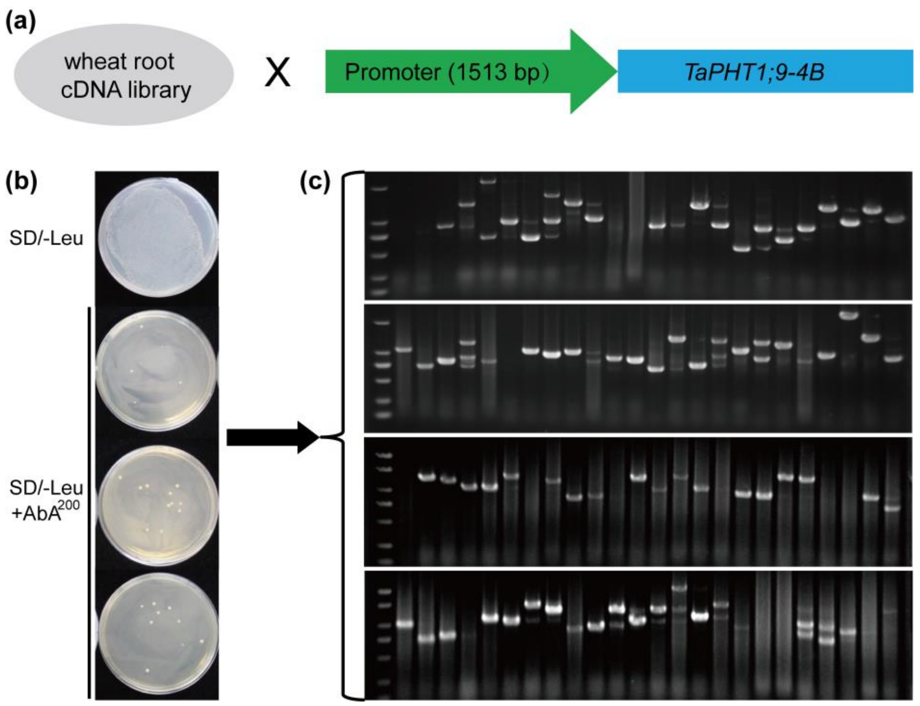

Fig. S12 Y1H screening using *TaPHT1;9-4B* promoter as bait. (a) Schematic diagram of Y1H screening. The cDNA library developed using the total RNAs of Pi starved wheat roots was screened using *TaPHT1;9-4B* promoter as bait. (b) Growth of yeast colonies on the SD/-Leu media containing 0 or 200 ng ml^{-1} aureobasidin A (AbA) at 30 ℃ for 3 d. The colonies showing growth on the AbA containing plates were putative positive clones. (c) PCR analysis of putative positive clones. PCR amplification was performed for the putative positive clones using the primers T7-F and 3-AD (Table S2).

(a)

```
1   ATGGGGAGATCGCCGTGCTGCGAGAAGGCGCACACCAACAAGGGCGCGTGGACGAGGGAGGAGGACGAGCGGCTGGTGGCCCACGTCCGG
1    M  G  R  S  P  C  C  E  K  A  H  T  N  K  G  A  W  T  R  E  E  D  E  R  L  V  A  H  V  R

91  GCGCACGGGGAGGGCTGCTGGCGCTCGCTGCCCAGCGCCGCCGGCCTGCTGCGCTGCGGCAAGAGCTGCCGCCTCAGGTGGATCAACTAC
31   A  H  G  E  G  C  W  R  S  L  P  S  A  A  G  L  L  R  C  G  K  S  C  R  L  R  W  I  N  Y

181 CTCCGCCCCGACCTCAAGCGCGGCAACTTCAGCCGCGACGAGGACGAGCTCATCGTCAAGCTCCATAGCCTCCTCGGCAACAAGTGGTCG
61   L  R  P  D  L  K  R  G  N  F  S  R  D  E  D  E  L  I  V  K  L  H  S  L  L  G  N  K  W  S

271 CTCATCGCCGCGCGCCTGCCCGGGAGGACGGACAACGAGATCAAGAACTACTGGAACACGCACATCCGGAGGAAGCTGCTGGGCAGGGGG
91   L  I  A  A  R  L  P  G  R  T  D  N  E  I  K  N  Y  W  N  T  H  I  R  R  K  L  L  G  R  G

361 ATCGACCCGGTCACGCACCGCCCCCTCACCGACGCCGCCACCGTCTCCTTCGTCCATCCTGCAGAGGCGACCAAGCAACAGGCGACGGAG
121  I  D  P  V  T  H  R  P  L  T  D  A  A  T  V  S  F  V  H  P  A  E  A  T  K  Q  Q  A  T  E

451 GAGAGGAAGCCGCCCAGATGCCCGGACCTCAACCTGGACCTCTGCATCAGCCTGCCGTTCCAACAGGAGGAGGAACGGCCGCCGGCGAGA
151  E  R  K  P  P  R  C  P  D  L  N  L  D  L  C  I  S  L  P  F  Q  Q  E  E  E  R  P  P  A  R

541 GCGTGCGCCAAGCCGGTGAAGATGGAGCAGCTGCAGCAGGGCGGCATCTGCTTCCGCTGCAGCATCCTCAGAGTGAGAGGAGCGGCGACG
181  A  C  A  K  P  V  K  M  E  Q  L  Q  Q  G  G  I  C  F  R  C  S  I  L  R  V  R  G  A  A  T

631 GAGTGCAGCTGCGGCAGCAACTTCCTGGGCCTCAGGGCCGGCATGCTCGACTTCAGAGGCCTCGAGATGAAATAG
211  E  C  S  C  G  S  N  F  L  G  L  R  A  G  M  L  D  F  R  G  L  E  M  K  *
```

(b)

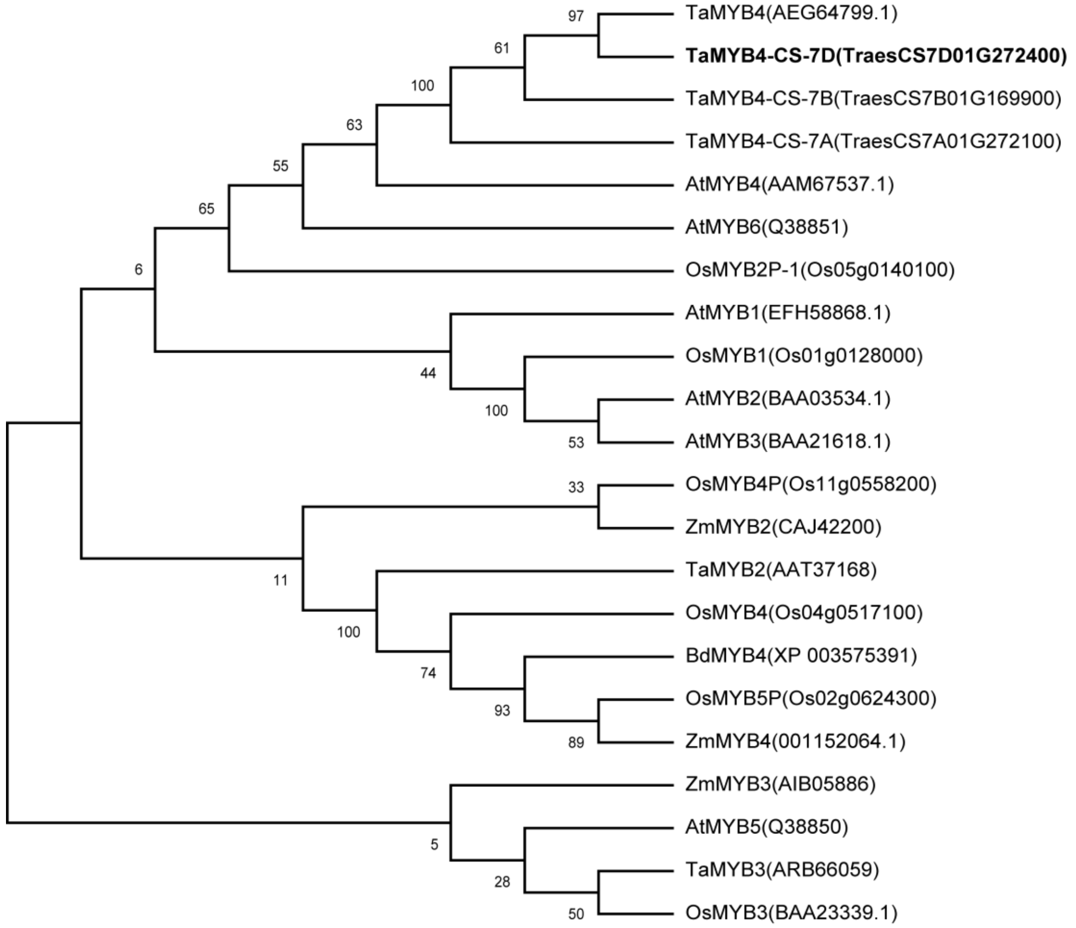

Fig. S13 Sequences and phylogenetic tree of *TaMYB4-7D*. (a) The nucleotide and deduced amino acid sequences of *TaMYB4-7D*. The fragment used for BSMV-VIGS analysis of *TaMYB4* is underlined. (b) Phylogenetic tree of representative plant MYB4 proteins. The tree shown was constructed using the neighbor-joining software with default parameter at the MEGA website (https://www.megasoftware.net/). The TaMYB4-7D homoeolog isolated in this work was identical to its counterpart in CS (TaMYB4-CS-7D), which clustered tightly with the TaMYB4 reported previously (Ma *et al.*, 2011). The bootstrap values were obtained using 1000 permutations. The GenBank accession numbers of the compared MYB4 sequences from various plant species are provided in the brackets.

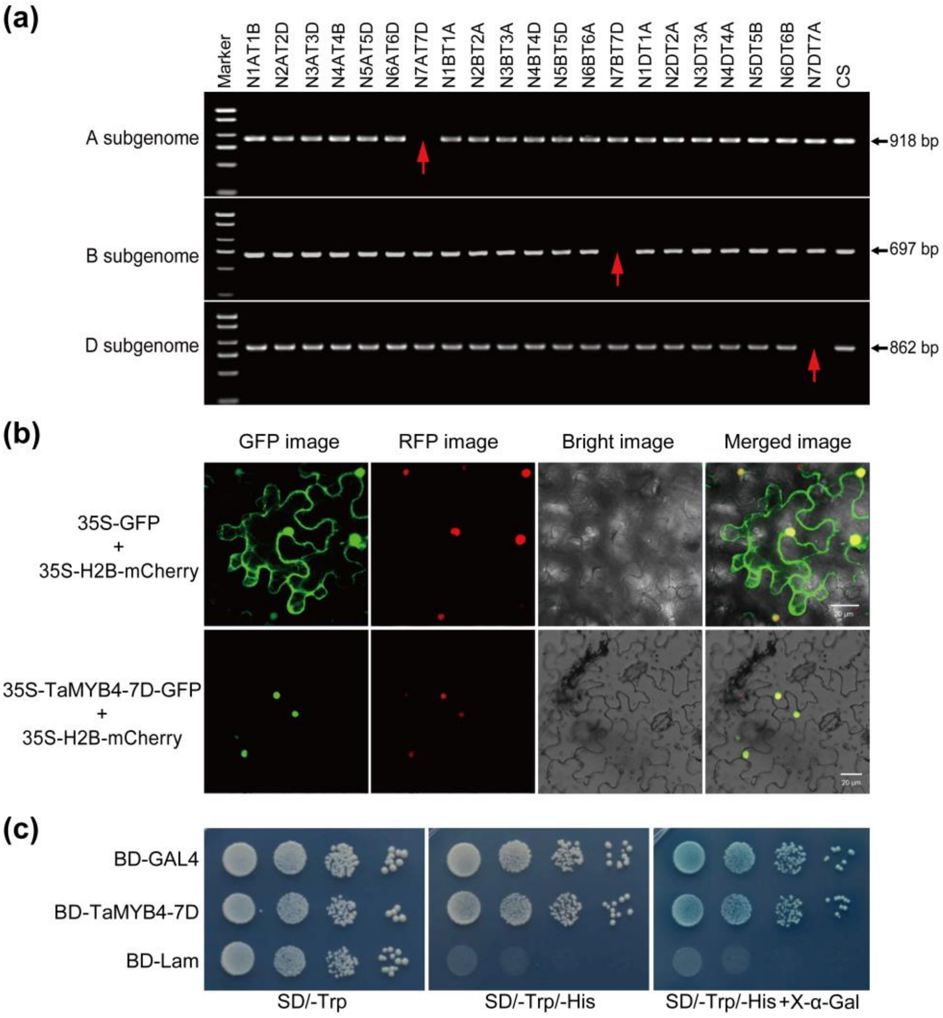

Fig. S14 Chromosomal location, subcellular localization, and transcriptional activation activities of TaMYB4-7D. (a) Chromosomal location of three *TaMYB4* homoeologs as analyzed using the nullisomic-tetrasomic (NT) lines of CS. PCR amplification was performed using the genomic DNA samples of CS and 21 derivative NT lines. Red arrows indicate the lack of anticipated amplicons for the NT lines N7AT7D, N7BT7D, and N7DT7A, thus indicating that the three homoeologs of *TaMYB4* were located on chromosomes 7A, 7B and 7D, respectively. (b) Subcellular localization of TaMYB4-7D in tobacco leaves. TaMYB4-7D-GFP fusion protein or GFP alone (control) were transiently expressed in *N. benthamiana* leaf epidermal cells through agroinfiltration. The images shown were taken under a confocal microscope at 2 d after the infiltration. TaMYB4-7D-GFP fusion protein was found localized in the nucleus. A H2B-mCherry fusion protein was used as a positive nuclear marker. Bar = 20 μm. (c) Analysis of the transcriptional activation activities of TaMYB4-7D in the yeast strain AH109. The construct pGBKT7-TaMYB4-7D (expressing the fusion protein BD-TaMYB4-7D) and the control vectors (pGBKT7-GAL4 and pGBDKT7-Lam) were separately transformed into the yeast cells. The yeast strains carrying pGBKT7-GAL4 (expressing BD-GAL4) or pGBDKT7-Lam (specifying BD-Lam) were used as positive and negative controls, respectively. The transformants were firstly screened on the SD/-Trp medium, followed by analysis of transcriptional activation activities of TaMYB4-7D on the SD/-Trp-His medium and the SD/-Trp-His medium supplemented with X-α-Gal. The datasets displayed were each representative of three independent experiments.

CAGGTTCTGCTCTCACGATGATATAATGCCGCAGCTATATTATGAGCTGTGATTCGCCGGCCGGACCGATTGCAATTTGGCAAAGAGAAACGAAGAAAGTGGGAG

GATGCAAGACAACAATTTAAATAGTACGGAGCACGGCTTTCCATGCTGTGCTCTTGCTGCTTAATCTGCATATGTTTAGGAGTAGTTTGTTACAAAAAACTGTTT

AGTACTGTAGTTCATATTTCAAATGACCAATGTTTATATGGCTCTGTTGCTATTTGTACTGAGCGTTGTCTGAATTCTGAAGTTATTTTCCTTTGTTTCTGTGAC

AACCATTTCATGCACCTAATCCTTCATGAAAATTTCTTCATGTTTCCTATATTAGAACCAAACAACTTTTAATACATATCCCTTGTTTGCAGTTCATGTAGAATT

CAAGTCAAATAACACAAAGTACAAGACATGTTTAACACCATCCACCTAATCCTTCAACAAAATTTCCTAATCCTTTAAGAAAAATTCTAGGTTTTTCCTGTGCTA

GAACCAAGCAATTTCTATGCAGATTCCCGTGTTCTCTATTCATGTAGAATTCAAAATTGTATGGCATCACAATTCTACATTTTTGGCTATGCATTTTTAGTATCA

CGTGGACCAACGAGACCCTTAATCTTTATGTTTATTTGTTTGTATACTATATATAAAAAACTATAAATGTTCTGAAACATGTGGCTGCTGCATTCTATTTTGTTC

TGAAACGTATCTGAATTCTGAAATTGGGTTTAGCAAGCGTTGAAGAACAATTTCAAACATCGTATTTGTATGACAAGCTATCTCAAAAATACTGAAAAGACCGTA

TGTGGCTATGTCTGGAGGACCTAGCACTGCATATCCATTTATCATCAAAATTCTGTATTTTCTTGCTTTCCTCTTGGCCTACCGAAACCGGCGGTGCGCTTACCT

TCTTCCTGCCATGCCATGTAGGTACGGAGGAGAGACAAATCACCAAATCCATGCTTGTAGTGAGAACATAAGATACAGATCATCGTATGATCTTGACCTTTTCTC

CCAAAGCTTGCTTGTGTCTGTACAGTCTGTTCCTTTCAGGTATATTCGCAGCATAACCATGGTCTGGTTTCAGTATTTTCCTCCACACAATTCTATAGTGCGGAA

ACTCAAAGGTCAAACCTGAATACTAAAAGTTGGCATTTCTTAACAATAGTTCTAACAATAGAAATGTACATAGCTGTTGCTGTCAAGGCAAGACATTTGACCCTT

GGGTATATTTTTGTAGGAACCATCCTGATATGTACAATCCGTTGTACTTGTAAGCGATGCCGTAATAGGCAATTTTATACTTACAACTAGCAATGTTGGATTGGC

ATAGTACATTATCTCCTTGCCTTTATGCCTTCACCGTGAATTATGCCTATATATACGTAGCAGAAGCACCTAACAAAGCACACAGGCTAGAGAGCAACAGAAGAA

AGATAGAAAGGAGCAGAGTTTAGCTGACAGCTCGCCGGCGGCCATG

Fig. S15 Sequence of *TaPHT1;9-4B* promoter. The predicted MYB binding sites (MBSs) and the translation start codon (ATG) are marked by green and red colors, respectively.

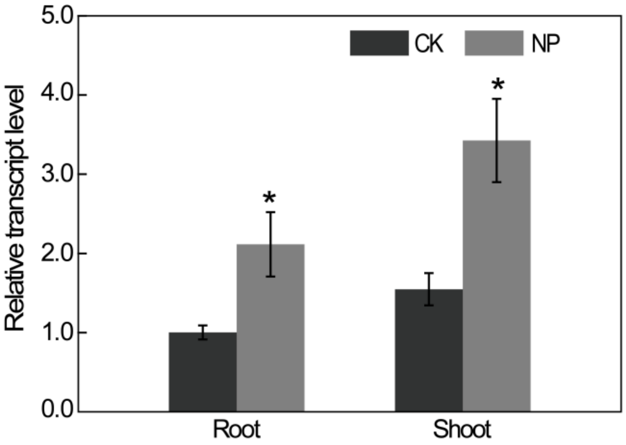

Fig. S16 Transcript level of *TaMYB4* in the root and shoot tissues of the wheat plants cultured under Pi sufficient or deficient media for 8 d. Two-week-old wheat seedlings were separately transferred to Pi sufficient (CK, 1 mM Pi) and deficient (NP, 0 mM Pi) Hoagland media for 8 d before being used for qRT-PCR assays using gene specific primers (Table S2). Transcript levels were normalized to the internal control of *TaActin*. Data represent means ± SD of three biological replicates, and asterisks indicate statistically significant differences ($P < 0.05$, Student's t-test).

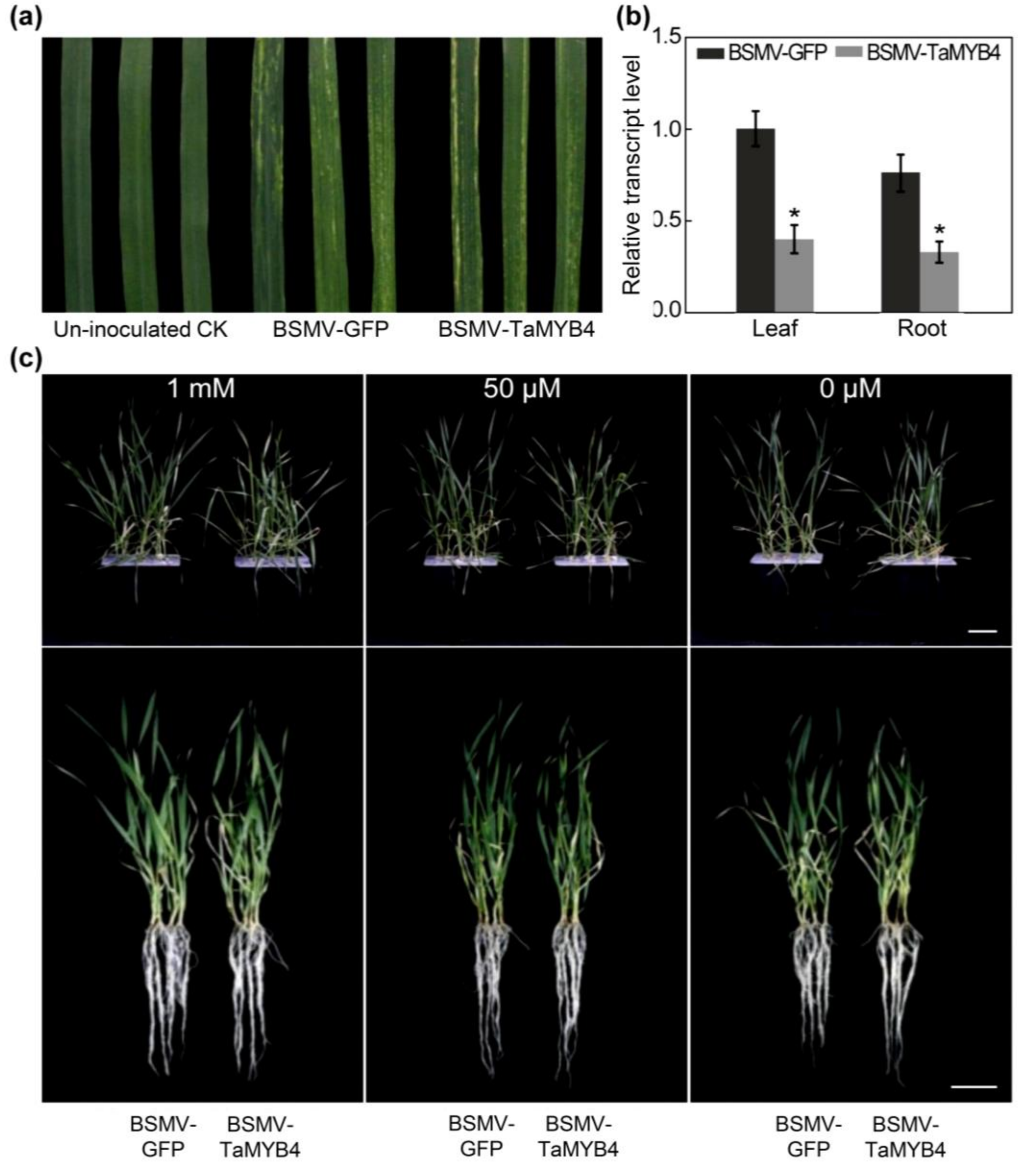

Fig. S17 Analysis of the bread wheat plants with *TaMYB4* expression silenced by BSMV-VIGS. (a) Viral symptoms, including chlorosis and mild mosaic, on the fourth leaves of the plants infected by BSMV-TaMYB4 or BSMV-GFP recorded at 7 d post inoculation. No such symptoms were observed on the un-inoculated controls (CK). (b) The transcript levels of *TaMYB4* in the leaf and root tissues of the plants infected by BSMV-GFP or BSMV-TaMYB4 as analyzed by qRT-PCR at 7 d post virus inoculation. The transcript level of *TaMYB4* in the leaf tissues infected by BSMV-GFP was set as 1 to facilitate cross comparison among the four treatments. Amplification of *TaActin* was used as an internal control. *TaMYB4* expression was significantly lowered in wheat leaves and roots by BSMV-VIGS. (c) Phenotypes of the plants infected by BSMV-GFP or BSMV-TaMYB4 after culturing under Pi sufficient (1 mM), low Pi (50 μM), or Pi deficient (0 μM) conditions for 10 d. Bars, 5 cm. Data represent means ± SD of three biological replicates; asterisks indicate statistically significant differences ($P < 0.05$, Student's *t*-test). The primer sequences are listed in Table S2.

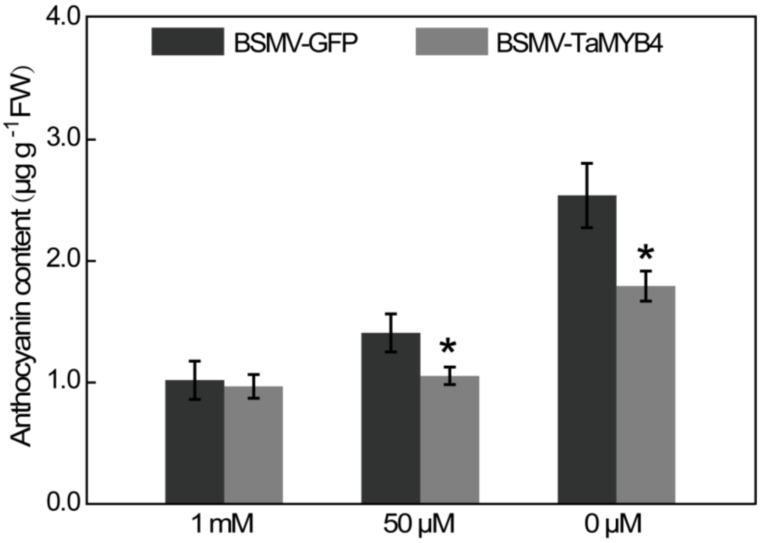

Fig. S18 Evaluation of foliar anthocyanin contents in the wheat plants infected by BSMV-GFP or BSMV-TaMYB4. The wheat plants infected by BSMV-GFP (control) or BSMV-TaMYB4 (silencing *TaMYB4*) were cultured under Pi sufficient (1 mM), low Pi (50 μM) or Pi deficient (0 μM) Hoagland solutions for 10 d before being used for measuring anthocyanin contents. Data represent means ± SD of three biological replicates, and asterisks indicate statistically significant differences ($P < 0.05$, Student's *t*-test).

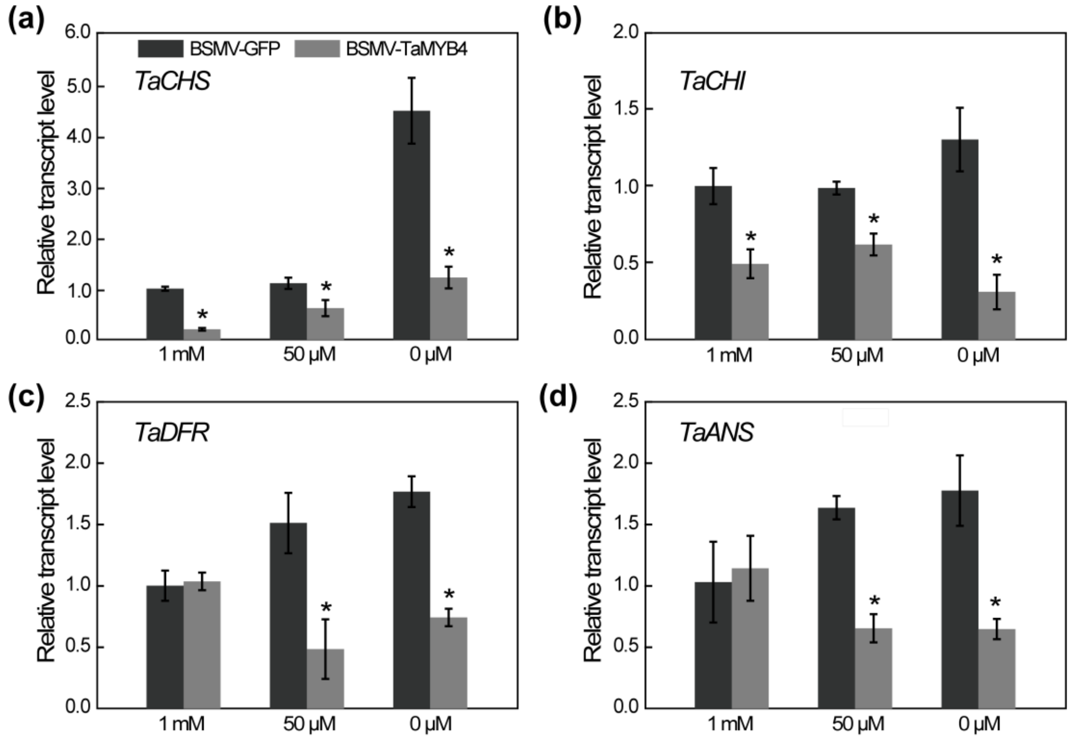

Fig. S19 Evaluation of the transcript levels of four anthocyanin biosynthesis genes in the leaves of BSMV-GFP or BSMV-TaMYB4 infected wheat plants. (a) *TaCHS* (encoding chalcone synthase, gi: AB187025), (b) *TaCHI* (coding for chalcone isomerase, gi: AB187026), (c) *TaDFR* (specifying dihydroflavonol 4-reductase, gi: AY373831), (d) *TaANS* (encoding anthocyanidin synthase, gi: MF620095). The wheat plants infected by BSMV-GFP (control) or BSMV-TaMYB4 (silencing *TaMYB4*) were cultured under Pi sufficient (1 mM), low Pi (50 μM), or Pi deficient (0 μM) media for 10 d before being used for qRT-PCR assays with gene specific primers (Table S2). Transcript levels were normalized to the internal control of *TaActin*. Data represent means ± SD of three biological replicates, with asterisks indicating statistically significant differences ($P < 0.05$, Student's t-test).

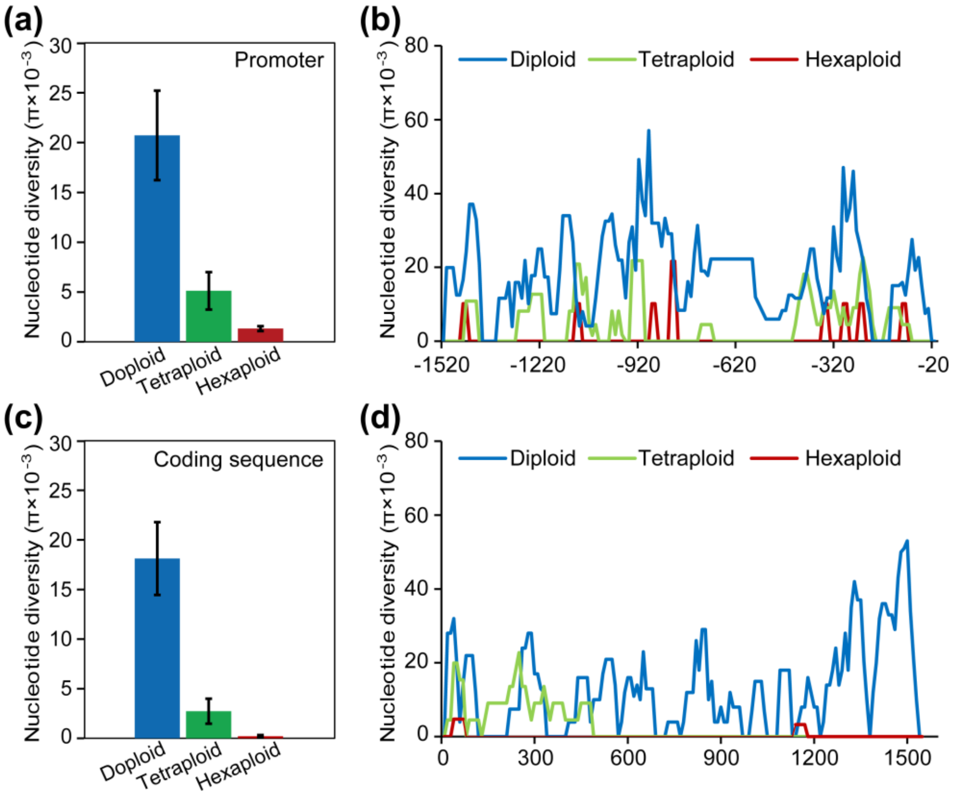

Fig. S20 Analysis of nucleotide diversity of *PHT1;9-4B* promoter and its genomic coding sequence in bread wheat and relatives. (a and b) π values (a) and their distribution patterns (b) along *PHT1;9-4B* promoter region in diploid (blue), tetraploid (green), and hexaploid (red) wheat accessions. (c and d) π values (c) and their distribution patterns (d) along *PHT1;9-4B* genomic coding region in diploid (blue), tetraploid (green), and hexaploid (red) wheat accessions. Data in (a) and (c) represent means ± SD of nucleotide differences among different sequences. The X-axes in (b) and (d) indicate the nucleotide positions in the promoter or genomic coding sequences relative to the translation start codon (ATG).

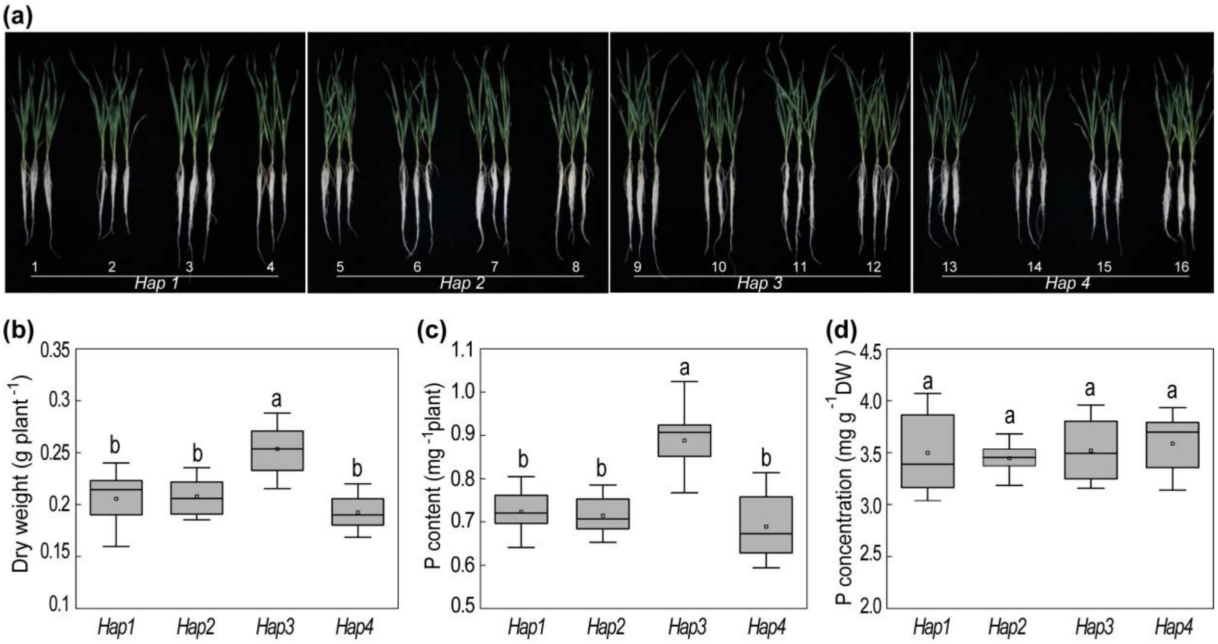

Fig. S21 Phenotypes (a), dry weights (b), P contents (c), and P concentrations (d) of the 16 wheat varieties with different promoter haplotypes (*Hap1-4*) of *TaPHT1;9-4B* cultured under low Pi conditions. Two-week-old seedlings were transferred to the low Pi (50 μM) medium, and cultured for 14 days before being used for the analysis. Bars, 5 cm. Data represent means ± SD of three biological replicates. Different letters indicate statistically significant differences (P < 0.05, one-way ANOVA followed by Duncan's multiple range test).

```
Hap1-PHT1;9-4B-Pro GCTATTTGTACTGAGCGTTGTCTGAATTCTGAAGTTATTTTCCTTTGTTTCTGTGACAACCATTTCATGCACCTAATCCTTCATGAAAATTTCTTC -1160
Hap2-PHT1;9-4B-Pro GCTATTTGTACTGAGCGTTGTCTGAATTCTGAAGTTATTTTCCTTTGTTTCTGTGACAACCATTTCATGCACCTAATCCTTCATGAAAATTTCTTC -1160
Hap3-PHT1;9-4B-Pro GCTATTTGTACTGAGCGTTGTCTGAATTCTGAAGTTATTTTCCTTTGTTTCTGTGACAACCATTTCATGCACCTAATCCTTCATGAAAATTTCTTC -1160
HaP4-PHT1;9-4B-Pro GCTATTTGTACTGAGCGTTGTCTGAATTCTGAAGTTATTTTCCTTTGTTTCTGTGACAACCATTTCATGCACCTAATCCTTCATGAAAATTTCTTC -1160

Hap1-PHT1;9-4B-Pro ATGTTTCCTATATTAGAACCAAACAACTTTTAATACATATCCCTTGTTTGCAGTTCATGTAGAATTCAAGTCAAATAACACAAAGTACAAGACATG -1064
Hap2-PHT1;9-4B-Pro ATGTTTCCTATATTAGAACCAAACAACTTTTAATACATATCCCTTGTTTGCAGTTCATGTAGAATTCAAGTCAAATAACACAAAGTACAAGACATG -1064
Hap3-PHT1;9-4B-Pro ATGTTTCCTATATTAGAACCAAACAACTTTTAATACATATCCCTTGTTTGCAGTTCATGTAGAATTCAAGTCAAATAACACAAAGTACAAGACATG -1064
Hap4-PHT1;9-4B-Pro ATGTTTCCTATATTAGAACCAAACAACTTTTAATACATATCCCTTGTTTGCAGTTCATGTATAATTCAAGTCAAATAACACAAAGTACAAGACATG -1064

Hap1-PHT1;9-4B-Pro TTTAACACCATCCACCTAATCCTTCAACAAAATTTCCTAATCCTTTAAGAAAAATTCTAGGTTTTTCCTGTGCTAGAACCAAGCAATTTCTATGCA  -968
Hap2-PHT1;9-4B-Pro TTTAACACCATCCACCTAATCCTTCAACAAAATTTCCTAATCCTTTAAGAAAAATTCTAGGTTTTTCCTGTGCTAGAACCAAGCAATTTCTATGCA  -968
Hap3-PHT1;9-4B-Pro TTTAACACCATCCACCTAATCCTTCAACAAAATTTCCTAATCCTTTAAGAAAAATTCTAGGTTTTTCCTGTGCTAGAACCAAGCAATTTCTATGCA  -968
Hap4-PHT1;9-4B-Pro TTTAACACCATCCACCTAATCCTTCAACAAAATTTCCTAATCCTTTAAGAAAAATTCTAGGTTTTTCCTGTGCTAGAACCAAGCAATTTCTATGCA  -968

Hap1-PHT1;9-4B-Pro GATTCCCGTGTTCTCTATTCATGTAGAATTCAAAATTGTATGGCATCACAATTCTACATTTTTGGCTATGCATTTTTAGTATCACGTGGACCAACG  -872
Hap2-PHT1;9-4B-Pro GATTCCCGTGTTCTCTATTCATGTAGAATTCAAAATTGTATGGCATCACAATTCTACATTTTTGGCTATGCATTTTTAGTATCACGTGGACCAACG  -872
Hap3-PHT1;9-4B-Pro GATTCCCGTGTTCTCTATTCATGTAGAATTCAAAATTGTATGGCATCACAATTCTACATTTTTGGCTATGCATTTTTAGTATCACGTGGACCAACG  -872
Hap4-PHT1;9-4B-Pro GATTCCCGTGTTCTCTATTCATGTAGAATTCAAAATTGTATGGCATCACAATTCTACATTTTTGGCTATGCATTTTTAGTATCACGTGGACCAACG  -872

Hap1-PHT1;9-4B-Pro AGACCCTTAATCTTTATGTTTATTTGTTTGTATACTATATATAAAAAACTATAAATGTTCTGAAACATGTGGCTGCTGCATTCTATTTTGTTCTGA  -776
Hap2-PHT1;9-4B-Pro AGACCCTTAATCTGTATGTTTATTTGTTTGTATACTATATATAAAAAACTATAAATGTTCTGAAACATGTGGCTGCTGCATTCTATTTTGTTCTGA  -776
Hap3-PHT1;9-4B-Pro AGACCCTTAATCTTTATGTTTATTTGTTTGTATACTATATATAAAAAACTATAAATGTTCTGAAACATGTGGGTGGTGCATTCTATTTTGTTCTGA  -776
Hap4-PHT1;9-4B-Pro AGACCCTTAATCTTTATGTTTATTTGTTTGTATACTATATATAAAAAACTATAAATGTTCTGAAACATGTGGCTGCTGCATTCTATTTTGTTCTGA  -776

Hap1-PHT1;9-4B-Pro AACGTATCTGAATTCTGAAATTGGGTTTAGCAAGCGTTGAAGAACAATTTCAAACATCGTATTTGTATGACAAGCTATCTCAAAAATACTGAAAAG  -680
Hap2-PHT1;9-4B-Pro AACGTATCTGAATTCTGAAATTGGGTTTAGCAAGCGTTGAAGAACAATTTCAAACATCGTATTTGTATGACAAGCTATCTCAAAAATACTGAAAAG  -680
Hap3-PHT1;9-4B-Pro AACGTATCTGAATTCTGAAATTGGGTTTAGCAAGCGTTGAAGAACAATTTCAAACATCGTATTTGTATGACAAGCTATCTCAAAAATACTGAAAAG  -680
Hap4-PHT1;9-4B-Pro AACGTATCTGAATTCTGAAATTGGGTTTAGCAAGCGTTGAAGAACAATTTCAAACATCGTATTTGTATGACAAGCTATCTCAAAAATACTGAAAAG  -680

Hap1-PHT1;9-4B-Pro ACCGTATGTGGCTATGTCTGGAGGACCTAGCACTGCATATCCATTTATCATCAAAATTCTGTATTTTCTTGCTTTCCTCTTGGCCTACCGAAACCG  -584
Hap2-PHT1;9-4B-Pro ACCGTATGTGGCTATGTCTGGAGGACCTAGCACTGCATATCCATTTATCATCAAAATTCTGTATTTTCTTGCTTTCCTCTTGGCCTACCGAAACCG  -584
Hap3-PHT1;9-4B-Pro ACCGTATGTGGCTATGTCTGGAGGACCTAGCACTGCATATCCATTTATCATCAAAATTCTGTATTTTCTTGCTTTCCTCTTGGCCTACCGAAACCG  -584
Hap4-PHT1;9-4B-Pro ACCGTATGTGGCTATGTCTGGAGGACCTAGCACTGCATATCCATTTATCATCAAAATTCTGTATTTTCTTGCTTTCCTCTTGGCCTACCGAAACCG  -584

Hap1-PHT1;9-4B-Pro GCGGTGCGCTTACCTTCTTCCTGCCATGCCA                                                                   -553
Hap2-PHT1;9-4B-Pro GCGGTGCGCTTACCTTCTTCCTGCCATGCCA                                                                   -553
Hap3-PHT1;9-4B-Pro GCGGTGCGCTTACCTTCTTCCTGCCATGCCA                                                                   -553
Hap4-PHT1;9-4B-Pro GCGGTGCGCTTACCTTCTTCCTGCCATGCCA                                                                   -553
```

Fig. S22 Nucleotide sequence comparison of the DNA fragments used to differentiate four *TaPHT1;9-4B* promoter haplotypes (*Hap1* to *Hap4*). The aligned fragments were all 703 bp, which were located from -1255 bp to -553 bp upstream of the start codon of *TaPHT1;9-4B*. The five underlined nucleotides were used to develop the CAPS marker CAPS-799.

(a)

AGAAATGTCACAAATTCCAAGAATATTTGGGAAATTCAAAAGTTGTTTGTGTTTCCAAATTTTGTTTTGAAATTTGAAAAATTGTTCCAGTTCAGTTTTTTGGGT
AGTTTAAAAAATGTTTCCGTTCCAAAAAAATATGTTCGCGTCTCCAAAATTCATTTGTGTGTTGAAAAAGTGTTTGAGTTTTCTCAAAATTTGTAAAATAAAACT
AATAGTGTTCGCGTTTGATAAAATATTCACCTTTTGTTTTTGCTATTTGGGAAATCGTAAAGTATGTTTTTCAAACAGTCTTATACTTTCTTTAGTTCTTGTAGG
TACCCGCTGCAGTTTGGTCAAATGAGCTCGTCCATCCTAGTGGCTAGTAGGGTGCGCGTGCGAGTCGCCAGTGCTGAGTTTGCACCCTACTATTTTTTGCAATTT
ATCACCTGGTATTTGCAGCGCACTGCAATGGGCCGGCCCAATCAGCGCACCGACGACGTGCCTGTGCGAAAGTGCAGCATTTTGACGCAAAATGCGTCACATAGG
AACTCCCTTAATATTTTGTCTCTGATGTTGATAGGTTGCATTCAGATCATATTATTTCCCATGCCGGATGATATCCTTGTCAACGATGGAGCATGGAAATTCCCC
AGATGTAACAGCTTTACATGCGGTGAAACTTCATTCTCGCGGGAGCCTAGACAAGAGAACATATAATAATAATATCAATATTCGCGGTGACACCCAGCTCTCCTC
GGCTACTTATACCAGACAGCCGGCCCATTATTTCTGCACCACAACACAAGGAAGATTTCCGGCCGGCCGGCACCGTCGTCTAGCTCTCACACTCGCCCCGTTGCC
CTGTGCAGCAGCAGCAGCAGCATCATG

(b)

CATTTCTCCCTGGCTTGACTGGCCGTTGATGAAGCTGCAGGAGCCAGCCCCCTAGCTAGCTATCTGTCTGCCTAGCTGCTTCGTCGGTGAGTCCTGCTTCCTCGC
CCGCTCGCTCGCTGGCCGGACTTGTATTAACAATACTATTTATTTCCCTTCTCAATTGGTGATCGGTTTTCAGTTTGGTAGTAGTAGTAGCAGCAGATTCTTTTG
TGATCACTGGACTTGCACATGCATGATTCCGGCTCTTAAACTGCTGCAGCACATCATCAGTTGTTGGTAGCTAGGAGTAGCTCCGGTGGTGCCCCGAAGTCGAGA
AAAGCCAAGAGAATCTTCCAAATTTCGTCTTTCTTTCTGACTAGCAAGTTGTGGTGAGGTGAGTGGAGGCAGTATCCTTTCCTCCGGAACTGAAACTCAACTCAA
AAGGCAGGCGGATCAAATCAACCCAACCCCACCTGCACCTGCAGCGAACTGACTCACTGACTGGCTCTGTTTTCTTGTCTTCTTCAGATCAGATGAGCCATCGAT
CCATCACGGAGCTCGATCGCTCCGTAGCCACGTCGCACTCCATTCTCTTTTCTTTTCTTTTCTTTTCTTTTCAAAAGTAGCCCAGGCAAGGCAATATATTTACAC
TTGTGTGTGCGCAACTAGTTCTATATATTTTTTTTCCTTTGAAAAGTCCACACCCCCCCATGCATGCGATGCGAGTGTCGAACAGTGCCCCGTCACGTCACCTTA
TAACTTGCCCCAAATTTCTTCACGCCTGCCTGACTCCTTGATCGTGACAAGAGCCTTCATTGGTTTCCTCTTTTCCAACCCTACCTCAACAATCACAACCGTACG
CACGAATATCTCATCTCGCCTGAAAAATAACGGATTTCTCTTTTCTTTTTATTATTATTAGAAAAGCAAAAATTGCAGATATGATTTTCTCGCCAACAAAATTGG
AATAAGATAACATGCCGGGCGAATATTCCCCCGGAGCGTAGAACAAAAACAACTGTCAGCCGCTTCGACTCGCCAGTAAAATCGTAACAGCCAAGGTTTATTCCC
AACGATCCCACCAAAATACCTTTTGATCCCAGCGTTCACAGCAGTCAACAACCAAGCCCAAGAGTCCCGGGGAAGCAGCAGGAGCCGCAGTATTTATCTCATCTC
TCATCCCACCCGACGCCCTCCATCCTCCTCCCACCCCACCCTAAAATCTCCAATCTTCTCTTGACAGGAACAAGAGATCCCCGGCGGCGATCCCCGGCGAGAGAT
CAGTCAAGGAGGGCTCGCGGCGATG

(c)

GTTTCCTGTGTTACAACCTACATCCAAACAACTTCTAATACATACCCCTTGTTTTCAGTTCATGTAGGATTTAAGTCAAATGACACAAAGTACAAGACATCTTTA
ACACCATGCACCTAATCCTTCAAGATTTTTTTTTACATTTGTCCTGTGGTACAGCCAAACAATGTCTATACAGATTCCCGTGTTCTATATTCATGTAGAATTCAA
AATTATATGGTGTCACAATTCTACATATTTGCTATGCGTTTCTAGTATCATGTGGACCAACGAGACCCTTAATCTGTATATGTCTATTAGTTTATATATGTAAAA
TGATCTCAAAAGTTTTATTTGTATGAGTATGACAAGCTATCTCAGAAATACGAAAGAAAAAAAAAAGTATGTGGCTGTCGGCAGGACCTAGCACTGCATATCCATT
TATCATCGAAGTTTTTTTTTTTTTCCCTCTGGCTTACTGAAATTGGCAGAGTGCCTACCTTCTTCCTGGCATACCATGTAGGTATCGAGGAGAGAACAAATTAACC
CCATATTTCAAGAAAGGTCACCTGTTCCGTACTAGTAGGGAGAACGTAAGATACAACTCATCGTATAATCTTGACCTTTTCTCCCAAAGCTTGCTTGTGTCTGTA
CATTCTGTTCCTTTCAGGTATATTCCCAGCATAACCATGATCTGGTTTCAGTATTTTTATCCCCACAATTCTATAGTACCGAAACTCAAAGGTCAAACCTGAAAA
CTAAAAGTTGGCATTTCTTAACAATCTTTCTAATAATAGAAATGTACCCCGACATAGCTGTAGCTCTCAAGGCAAGACATTTGACTCTGCGTATAATTTTTGTAA
GAACCATCCTGATATGTACAATCCGTTGTACTTGTAAGCGATGCCGTAATAGGCAATTTTATACTTACAACTAGCAATGTTGGATTGGCATACCACAATATTGTA
TGTCTTCACCCTGGATTATGCCTGCTATATATACGTATTAGAAGCACCGTACAAAGCACACAAACAAGAGAGAACCAGAAGAAGTACAGAAGGGGGCAGAAGTTT
AGTTGAGAGATCGCCGGCGGCCATG

Fig. S23 Nucleotide sequence of the promoter region of *TaPHT1;3-5B*, *TaPHT1;6-5B*, and *TaPT2*. The promoter region of *TaPHT1;3-5B* (a), *TaPHT1;6-5B* (b), and *TaPT2* (c) analyzed in this work was 864 bp, 1,282 bp, and 1,072 bp, respectively. The predicted MYB binding sites (MBSs) and the translational start codon (ATG) are highlighted with green and red colors, respectively.

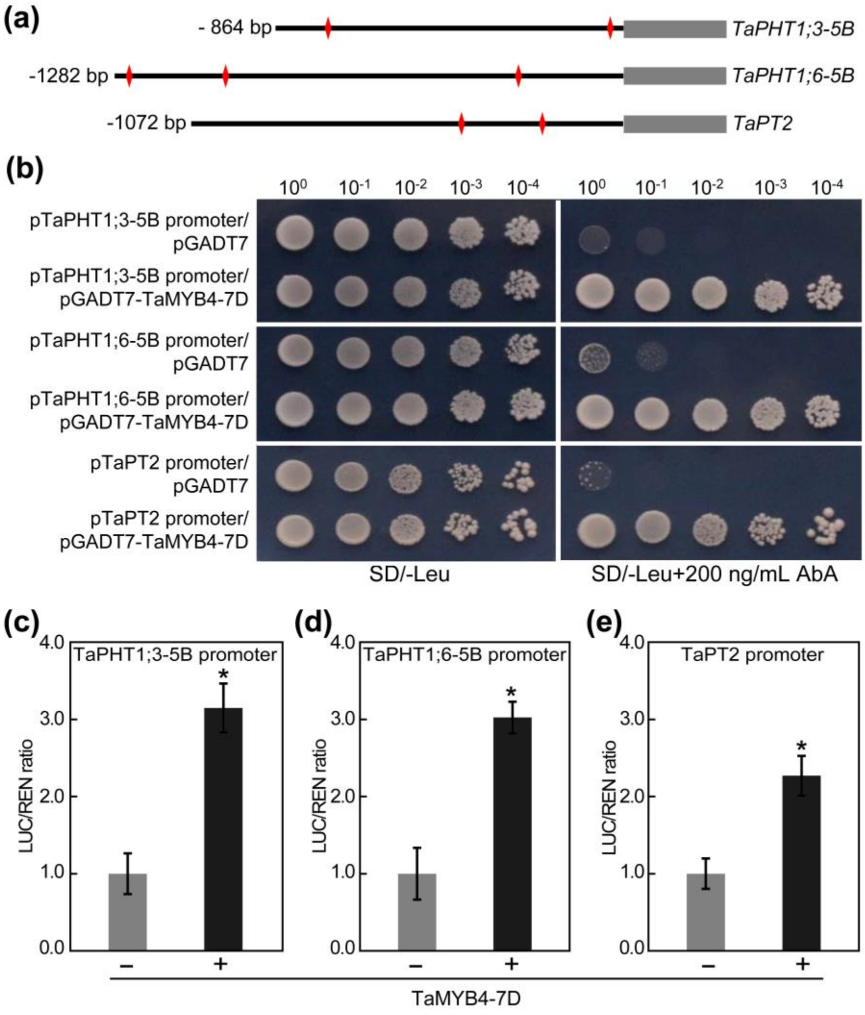

Fig. S24 Binding of TaMYB4-7D to the promoter region of *TaPHT1;3-5B*, *TaPHT1;6-5B*, and *TaPT2*. (a) Schematic representation of the promoter region of *TaPHT1;3-5B*, *TaPHT1;6-5B*, and *TaPT2* analyzed in this work. The predicted MYB binding sites are indicated by red diamonds. (b) Binding of TaMYB4-7D to the promoter region of *TaPHT1;3-5B*, *TaPHT1;6-5B*, and *TaPT2* as demonstrated using Y1H assay. The yeast cells co-transformed with the bait vector (pTaPHT1;3-5B promoter, pTaPHT1;6-5B promoter, or pTaPT2 promoter) and the test prey construct (pGADT7-TaMYB4-7D) grew well on the SD/-Leu plates containing 200 ng ml^{-1} aureobasidin A (AbA), but those co-transformed with the bait vector (pTaPHT1;3-5B promoter, pTaPHT1;6-5B promoter, or pTaPT2 promoter) and the empty prey construct (pGADT7) failed to grow under the same condition, indicating positive binding of TaMYB4-7D to the promoter region of *TaPHT1;3-5B*, *TaPHT1;6-5B*, and *TaPT2*. The yeast cells were cultured at 30 ℃, with the graphs shown taken at 3 d post plating the cells on the indicated media. (c-e) Ratios of LUC/REN activities obtained in the dual luciferase assays conducted for the promoter region of *TaPHT1;3-5B* (c), *TaPHT1;6-5B* (d), and *TaPT2* (e) in *N. benthamiana* leaves. In all three cases, the ratio of LUC/REN activities was significant higher in the presence (+) of TaMYB4-7D compared with the control in which TaMYB4-7D was absent (-). LUC and REN activities were measured at 2 d after agroinfiltration of the desired constructs in *N. benthamiana* leaves. Data represent means ± SD of three biological replicates. Asterisks indicate statistically significant differences (P < 0.05, Student's t-test). The datasets shown in (b) to (e) were each representative of two independent experiments.

References

Ma QH, Wang C, Zhu HH. 2011. TaMYB4 cloned from wheat regulates lignin biosynthesis through negatively controlling the transcripts of both cinnamyl alcohol dehydrogenase and cinnamoyl-CoA reductase genes. *Biochimie* **93:** 1179-1186.
