# Supplementary material for: TaPHT1;9‐4B and its transcriptional regulator TaMYB4‐7D contribute to phosphate uptake and plant growth in bread wheat
Source: New Phytol. 2021 Jul 2;231(5):1968–83. doi: 10.1111/nph.17534 (PMC8489284; doi:10.1111/nph.17534)
Supplement: Supplementary file 1 — Dataset S1 Identification and analysis of phosphate deficiency responsive proteins (PDRPs) in bread wheat roots using iTRAQ‐based proteomics. Dataset S2 Quantification analysis of 11 Pi‐deficiency responsive proteins (PDRPs) using PRM analysis in bread wheat roots. Dataset S3 Mapping PDRPs to the genomic loci of Chinese Spring. Fig. S1 Examples illustrating homoeolog mapping of proteomic peptides in this work. Fig. S2 Phenotype and growth parameters of wheat seedlings cultured under Pi‐deficient conditions for 10 d. Fig. S3 Comparison of 11 PDRPs with respect to their expression changes induced by Pi deficiency revealed using iTRAQ or PRM approaches. Fig. S4 Nucleotide and deduced amino acid sequence of TaPHT1;9‐4B. Fig. S5 Transcript level of TaPHT1;9 in the root and shoot tissues of the wheat plants cultured under Pi‐sufficient or ‐deficient media for 8 d. Fig. S6 The coding sequences of TaPHT1;9 homoeologs (4A, 4B and 4D) in Chinese Spring. Fig. S7 Functional analysis of TaPHT1;9 in bread wheat using BSMV‐VIGS. Fig. S8 Evaluation of the transcript levels of three TaPHT1 genes and TaIPS1.1 in the roots of the wheat plants infected by BSMV‐GFP or BSMV‐TaPHT1;9. Fig. S9 Molecular identification of transgenic rice lines expressing TaPHT1;9‐4B. Fig. S10 The effects of nucleotide mutations in three CRISPR mutants on TaPHT1;9‐4B protein. Fig. S11 Phenotypes, dry weights and P concentrations of three CRISPR mutants and WT Fielder control cultured under Pi‐sufficient conditions. Fig. S12 Y1H screening using TaPHT1;9‐4B promoter as bait. Fig. S13 Sequences and phylogenetic tree of TaMYB4‐7D. Fig. S14 Chromosomal location, subcellular localisation and transcriptional activation activities of TaMYB4‐7D. Fig. S15 Sequence of TaPHT1;9‐4B promoter. Fig. S16 Transcript level of TaMYB4 in the root and shoot tissues of the wheat plants cultured under Pi‐sufficient or ‐deficient media for 8 d. Fig. S17 Analysis of the bread wheat plants with TaMYB4 expression silenced by BSMV‐VIGS. [file NPH-231--s001.zip › nph17534-sup-0005-MethodsS1.pdf]

New Phytologist Supporting Information

Article title: TaPHT1;9-4B and its transcriptional regulator TaMYB4-7D contribute to phosphate uptake and plant growth in bread wheat

Authors: Pengfei Wang, Gezi Li, Guangwei Li, Shasha Yuan, Chenyang Wang, Yingxin Xie, Tiancai Guo, Guozhang Kang, Daowen Wang.

Article acceptance date: 25 May 2021.

Methods S1 Additional description of methods

Plant growth conditions and measurement of P concentration and anthocyanin content

Cv Zhoumai 18 seeds were sterilized with 0.01% mercuric chloride and washed thoroughly with distilled water. They were germinated in glass dishes (15 cm diameter; 60 seeds per dish) containing full-strength Hoagland solution and incubated in an FPG-300C-30D illumination chamber (Ningbo Laifu Technology Co. Ltd. China) at 25°C : 15°C, day : night temperature regime and with a 14 h:10 h, light : dark photoperiod, 60% day : 75% night relative humidity, and 250 µmol $m^{-2}s^{-1}$ light intensity. Two-week-old wheat seedlings were divided into two groups, one remained in the Hoagland solution containing sufficient Pi (1 mM, as control), and the other transferred to Pi deficient Hoagland medium (0 mM Pi, with KH_2PO_4 replaced by KCl). These seedlings were grown for 10 d, with nutrient solutions replenished at 8:00 am every day. The dry weight of root and shoot samples was determined after drying at 60°C for 72 h. To measure total P concentration, each sample (approximately 0.2 g crushed dry materials) was digested with H_2SO_4-H_2O_2 at 300°C. After cooling, the digested sample was diluted to 100 ml in distilled water. P concentration in the solution was measured using the molybdate-blue colorimetric method as detailed by Chen *et al.* (2007a). Anthocyanin contents in the leaves of the plants infected by BSMV-MYB4 or BSMV-GFP were determined as described previously (Ahmed *et al.,* 2009).

Identification and validation of PDRPs

Total proteins from the sampled roots of Pi sufficient (control) and Pi deficient wheat seedlings were extracted using the trichloroacetic acid (TCA)/acetone method. Three biological replicates with at least six plants per replicate were designed for both the control and the Pi starved roots. For each replicate, 300 µg total proteins were reduced, alkylated, and digested according to the filter-aided sample preparation procedure as described in our previous study (Kang *et al.,* 2015).

The digested peptide mixtures were labeled using the 8-plex iTRAQ reagent (Applied Biosystems, Foster, CA, USA) according to the manufacturer's instructions, with three independent 8-plex iTRAQ reagent biological replicates performed. Protein samples were labeled as 113–121 in each independent 8-plex iTRAQ reagent, respectively. 113-control roots and 115-Pi-deficient roots were used in this study, and the remaining six labels (114, 116–121) were used in other experiments. The labeled peptides were separated and desalted using strong cation exchange chromatography and a C18 Cartridge, respectively (Sigma, St. Louis, MO, USA), and then subjected to LC-MS/MS analysis. The cHiPLC Nanoflex microchip system (Eksigent Technologies, Dublin, CA, USA) with nanoLC-MS/MS (Nano Ultra 2D Plus, Eksigent, USA) equipped with an AB SCIEX TripleTOF 5600 MS (Toronto, Concord, Canada) was used for MS analysis. The acquired raw data files from the cHiPLC Nanoflex microchip system were converted into MGF files using Proteome Discoverer 1.4 (Thermo Electron, San Jose, CA), and the MGF files were searched against the non-redundant NCBI-Triticeae protein database (https://www.ncbi.nlm.nih.gov/taxonomy/?term= Triticeae, version 10/06/2018, 192,314 entries) for protein identification using the MASCOT engine software (Matrix Science, London, UK; version 2.2). The cRAP database (ftp://ftp.thegpm.org/fasta/cRAP) was next used to filter the identified protein species (Torabi *et al.,* 2009). For protein identification, the following parameters were used: ion search type = MS/MS, digestion enzyme = trypsin, fixed modification = carbamidomethyl (C), iTRAQ8plex (N-term), and iTRAQ8plex (K), variable modification = oxidation (M), mass values = monoisotopic, max missed cleavage = 2, fragment mass tolerance = 0.1 Da, peptide mass tolerance = ± 20 ppm, matched unique peptides ≥1, peptide false discovery rate (FDR) ≤ 0.01 (Sandberg *et al.,* 2012).

The unique peptide employed for abundance quantification of each identified protein species was automatically selected with the ProGroup algorithm (AB SCIEX) to calculate the reporter peak area. Bias correction for unequal mixing of the different labeled samples was performed to normalize protein quantification; this correction was based on the assumption that the expression of most protein species did not have prominent change. Only the protein species identified in all three biological replicates were considered for further analysis; the abundance of each identified protein was compared between Pi-deficient and Pi-sufficient roots to calculate the ratios of expression change for all PDRPs, and significant differences were evaluated using Duncan's multiple range test ($P < 0.05$). This multiple testing correction was then used to adjust P values for controlling the false discovery rate (FDR) and the Benjamini and Hochberg (BH) test (Li *et al.,* 2017). The mass spectrometry proteomics data obtained in this work have been deposited in the ProteomeXchange Consortium *via* the PRIDE partner repository (Ternent *et al.,* 2014).

To validate the expression changes of PDRPs, a PRM experiment was performed for a set of wheat root protein samples prepared as those used in the iTRAQ analysis. The experiment was executed on a Q-Exactive HF mass spectrometer equipped with an EASY nLC-1200 system (Thermo, USA) following the method described previously (Zauber *et al.*, 2018). The protein peptides to be identified and quantified by PRM were similarly prepared as those used in iTRAQ. Two known peptides (ADVTPADFSEWSK and DGLDAASYYAPVR) were included as internal controls. The resulting data were analyzed via the Skyline software (3.5.0). Three independent biological replicates were performed.

Identification of homoeolog-specific protein species

To identify homoeolog-specific protein species, the above peptide data were searched against the translated proteomes of A, B, and D subgenomes of CS (IWGSC *et al.,* 2018, RefSeq 1.0 version, Ensembl version 97.4), respectively, using BLASTP (version 2.6) and by applying a stringent sequence identity cut-off of 100%, with A, B, or D homoeolog specific peptides identified according to Hu *et al.* (2014). For example, among the five peptides obtained for a wheat flavonoid *O*-methyltransferase-like protein, one (VPSDGVVNYVAGDMFHTVPPAQAVMLK), two (QTAFPLAVTSR and ALPVPSPFEHVHAAAPFDESMTLLDHETDK), and one (LGGTAS-LADLMAALSLPPPK) were specific to the A (TraesCS5A02G521000), B (TraesCS4B02G352200) and D (TraesCS4D02G346800) subgenome homoeologs, respectively, with the remaining one (QIAFPLAVTSR) shared by all three subgenome homoeologs (Fig. S1a). Of the five peptides identified for a wheat peroxidase 2 protein, two (GCDASVLLDPTPENPK and GPDPIVPNNIRDEDAGAVAAAVPGFLPK) were mapped specifically to the A subgenome homoeolog (TraesCS1A02G0777), and the other three (LDGVHSHAAEAQQDLPDSTFTIAELI-QNFK, GPDPIVPNNIRDEDAGAVAATIPGFLPK, and GCDASVLLDPTPENPHTEK) were found specific to the D subgenome homoeolog (TraesCS1D02G079800), with no peptide mapped specifically to the B subgenome homoeolog (Fig. S1b). For the two peptides (KPEDWDDKEYIP-DPEDVKPEGYDDIPR and APMIANPDFKDDPYIYAFNSLK) identified for a wheat calreticulin-like protein, they were all mapped to the B subgenome homoeolog (TraesCS2B02G576100) (Fig. S1c). This calreticulin-like protein showed single homoeolog biased expression because no protein products were detected for its A and D subgenome homoeologs (Hu *et al.,* 2015). Finally, the significance of unbalanced expression among the distinct homoeologs of a protein was assessed by Student's *t*-test ($P < 0.05$) (Powell, *et al.*, 2017; Ramirez-Gonzalez *et al.,* 2018).

Yeast complementation assay

The yeast mutant MB192 with mutated high affinity Pi transporter PHO84 and the yeast expression vector p112A1NE were used for functional complementation assay of TaPHT1;9-4B following the protocol described previously (Bun-Ya *et al.,* 1991; Teng *et al.,* 2017). TaPHT1;9-4B CDS was subcloned into p112A1NE, with the resultant construct Yp112-TaPHT1;9-4B transformed into MB192 cells. These cells, as well as those of untransformed MB192 and wild type control, were grown to the logarithmic phase in YNB liquid medium, followed by harvesting and washing in Pi-free YNB medium. Subsequently, the three yeast strains were each inoculated into the YNB liquid media containing different concentrations of Pi (20, 60, or 100 μM), and cultured for 10 h at 30°C. Bromcresol purple was added to the media and acted as a pH indicator; a change of medium color from brown to yellow indicated acidification, which was correlated with the growth rate of yeast cells. In addition, the cells carrying Yp112-TaPHT1;9-4B and the controls were grown in the YNB liquid medium containing 60 μM Pi, and sampled at 5 time points (6, 12, 18, 24, and 30 h) for measuring optical density at 600 nm. To detect the effects of pH on Pi uptake, the cells carrying Yp112-TaPHT1;9-4B and the controls were cultured in YNB liquid media with different pH values (4, 5, 6, 6.5, 7, and 8) while maintaining a fixed concentration of K_2HPO_4 (60 μM) for 24 h, followed by measuring the optical density at 600 nm. For each experiment, three biological replicates were measured for each of the three yeast strains.

BSMV-VIGS mediated transient silencing of *TaPHT1;9* and *TaMYB4*

A conserved cDNA fragment (256 bp, from +647 to +902, +1 from the start codon ATG), which shares 98.04% similarities among three *TaPHT1;9* homoeologs (Fig. S6), was used to construct a silencing vector (BSMV-TaPHT1;9). This fragment was inserted into the *Nhe* I site of BSMV-γ plasmid in an antisense orientation. BSMV-GFP was used as a negative control (Lee *et al.,* 2015). The primers used for the construction are listed in Table S2 and the BSMV-VIGS vectors are illustrated in Fig. S7a. *In vitro* transcription of viral RNAs was performed as described in our previous studies (Ma *et al.,* 2012; Liu *et al.,* 2016). The plasmids of BSMV-α, BSMV-β, and BSMV-γ derivatives (BSMV-TaPHT1;9 or BSMV-GFP) were linearized with *Mlu*I and *Spe*I, respectively. RNA synthesis was performed using the RiboMAX™ Large Scale RNA Production System-T7 kit (Promega, Fitchburg, WI, USA) according to the manufacturer's instructions. The transcripts of RNA-α, RNA-β and RNA-BSMV-TaPHT1;9 (or RNA-BSMV-GFP) were mixed in a 1:1:1 ratio and subsequently diluted with 9 volumes of diethyl pyrocarbonate (DEPC)-treated water. Next, 12 volumes of 2× GKP buffer (50 mM glycine, 30 mM, pH 9.2 dipotassium

hydrogen phosphate, 1% bentonite, and 1% Celite) were added to the diluted transcript mixture for subsequent inoculation. Transcript inoculation was performed on the second leaves of two-week-old wheat seedlings (cv Zhoumai 18), and the inoculated plants were cultured in Pi sufficient (1 mM) Hoagland solution. At 8 d after inoculation, BSMV infection symptoms, i.e., chlorosis and mild mosaic on the leaves, were observed. The transcript levels of *TaPHT1;9* in the root and leaf tissues, which represented the total expression of all three homoeologs, were measured by qRT-PCR with the primers capable of amplifying all three homoeoalleles (Table S2). Subsequently, wheat seedlings infected by BSMV-TaPHT1;9 or BSMV-GFP were separately transferred to Pi sufficient (1 mM), low Pi (50 μM), or Pi deficient (0 μM) Hoagland solution for 10 d. Representative growth parameters (plant height, root length, and root or shoot dry weights) and P concentrations of the seedlings were determined.

The above protocol was also followed for silencing the expression of *TaMYB4* in Zhoumai 18 plants. In this case, the cDNA fragment used for preparing the silencing virus (BSMV-TaMYB4) came from the coding sequence of *TaMYB4-7D*, which was 217 bp, and exhibited 96.1 - 96.7% similarities to its corresponding regions in *TaMYB4-7A* and *-7B*.

CRISPR/Cas9 mediated editing of *TaPHT1;9-4B*

Owing to very high sequence similarity (> 98%) among the three homoeologs of *TaPHT1;9*, it was difficult to design a sgRNA specifically targeting *TaPHT1;9-4B*. Therefore, we chose to use a sgRNA of 20 nucleotides in size, which had one SNP between *TaPHT1;9-4B* and the other two homoeologs (*TaPHT1;9-4A* and *-4D*) (Fig. 4a). The sgRNA was inserted into the *pWMBX110-SpCas9* vector as described previously (Ma *et al.*, 2015; Liu *et al.*, 2020). The immature embryos of the wheat cultivar Fielder were used for *Agrobacterium*-mediated transformation to obtain transgenic plants (Wang *et al.*, 2017). Genomic DNA of T_0 plants was used for amplifying *TaPHT1;9-4A, -4B* and *-4D* with homoeolog specific primers, which were designed using polymorphic sequences flanking the 5' and 3' ends of the three homoeologs (Table S2). The amplified products were sequenced directly to identify CRISPR/Cas9-mediated mutations. The T_1 seeds harvested from individual T_0 plants were screened for homozygous mutants by sequencing *TaPHT1;9-4B* amplicons, with the homozygous mutant T_1 plants used for subsequent analysis. The two-week-old homozygous CRISPR mutant and the WT Fielder control plants, uniform in developmental stage and size, were transferred to Pi sufficient (1 mM), low Pi (50 μM), or Pi deficient (0 μM) Hoagland solutions for 21 d. Then their root or shoot dry weights and P concentrations were determined.

Ectopic expression of *TaPHT1;9-4B* homoeolog in rice

The coding sequence (CDS) of *TaPHT1;9-4B* was amplified and inserted into the pCUN1301 vector carrying an expression cassette directed by the Ubi1 promoter of maize ubiquitin 1 gene (Chen *et al.,* 2007b), yielding the T-DNA transformation construct pUbi1-TaPHT1;9-4B. This construct was transferred to the *Agrobacterium tumefaciens* strain EHA105, which was then used for *Agrobacterium*-mediated rice transformation (Hiei *et al.,* 1994; Upadhyaya *et al.*, 2000). Transgenic rice plants expressing *TaPHT1;9-4B* were identified by hygromycin resistance selection and PCR analysis as described previously (Wang *et al.,* 2015a). Two independent T_2 transgenic rice lines were selected for further analysis. Their seedlings were grown in an environmental chamber for 2 weeks in the IRRI nutrient solution for rice culture, with a 16 h: 8 h, light : dark photoperiod, a 30°C : 22°C, day : night temperature regime, a light intensity of 250 μmol $m^{-2}s^{-1}$, and 70% relative humidity. Afterwards, the transgenic rice seedlings were transferred into Pi sufficient (300 μM), low Pi (10 μM), and Pi deficient (0 μM) IRRI solutions, respectively (Sun *et al.,* 2012). The solution was refreshed every 3 d. At 3 weeks after transfer, the growth parameters and P concentrations were determined as described above using three biological replicates for both the control and treated plants.

Y1H assays

The promoter region (1,513 bp) of *TaPHT1;9-4B* was amplified and subcloned into the pAbAi vector to produce a bait construct, which was then linearized using *Bst*BI digestion and integrated into the *URA3-52* locus of the Y1H Gold yeast genome to generate a Y1H bait strain (Wang *et al.,* 2015b). The cDNA library used in Y1H screening was generated with total RNAs extracted from the roots of the bread wheat seedlings cultured under Pi deficient conditions for 8 day using SMART cDNA synthesis technology [TaKaRa Biotechnology (Dalian) Co., Ltd., Dalian, China]. The cDNA library and the linearized pGADT7-Rec vector were co-transformed into the Y1H bait strain containing *TaPHT1;9-4B* promoter, with the subsequent experimental steps performed using the Matchmaker Gold Yeast One-Hybrid Library Screening System kit (Clontech, Palo Alto, CA, USA) (Sun *et al,.* 2015). Approximately 1.0 million yeast transformants were screened, and potential positives that contained the prey proteins were isolated. Their plasmids were extracted, amplified in *Escherichia coli*, and sequenced commercially.

Y1H assay was also employed to validate the interaction between *TaPHT1;9-4B* promoter and TaMYB4-7D transcription factor. The coding sequence of TaMYB4-7D was cloned into the pGADT7 vector to produce the pGADT7-TaMYB4-7D construct. This construct and the empty

vector (as control) were separately transformed into the above Y1H bait strain, followed by selection on a synthetic dropout (SD)/-Leu plate containing 200 ng ml^{-1} aureobasidin (AbA). The selected yeast strains were separately incubated on the plates containing different concentrations of AbA (i.e., 0, 200, 300, and 500 ng ml^{-1}) (Kim *et al.,* 2007; Li *et al.,* 2018).

Further Y1H assays were performed to determine if TaMYB4-7D may specifically bind to the MBSs in *TaPHT1;9-4B* promoter. Sequence analysis indicated that *TaPHT1;9-4B* promoter contained 4 MBSs (-1260 to -1255, CTGTTG; -1199 to -1194, CAACCA; -285 to -280, CTGTTG; -215 to -210, CCGTTG) (Fig. S15). Four 46 bp promoter fragments containing different MBSs (P1, -1280 to -1235 bp; P2, -1219 to -1174 bp; P3, -305 to -260 bp; P4, -235 to -190 bp) and their mutants (mP1 to mP4) were prepared (Fig. 5e). They were used to generate 8 Y1H bait strains. The pGADT7-TaMYB4-7D construct was introduced into the 8 bait strains, with the co-transformants selected on the synthetic dropout (SD)/-Leu plates containing 150 ng ml^{-1} AbA.

Dual-luciferase assay

The dual-luciferase assay between *TaPHT1;9-4B* promoter and TaMYB4-7D was performed as previously described (Hellens *et al.,* 2005; Mao *et al.,* 2018). The coding sequence of TaMYB4-7D was subcloned into the pCAMBIA1301 vector under the control of 35S promoter, which yielded the effector construct. *TaPHT1;9-4B* promoter was inserted into the pGreenII0800-LUC vector, resulting in the reporter construct. The primers used in these cloning experiments are listed in Table S2. The effector and reporter constructs were each transformed into the *Agrobacterium* strain GV3101. The resultant strains were used to infiltrate the leaves of *N. benthamiana*. After the infiltration, the tobacco plants were incubated at 25°C for 3 d, with the firefly luciferase (LUC) and renilla luciferase (REN) activities in the tobacco leaves measured using the Dual-Luciferase Reporter Assay System (Promega, Fitchburg, WI, USA) according to the manufacturer's instructions. The promoter activities shown by the reporter construct were determined by calculating the ratio of LUC to REN. The tobacco leaves co-infiltrated with the *Agrobacterium* cells carrying the reporter construct or the empty pCAMBIA1301 vector were used as a control for the dual luciferase assay.

Nucleotide diversity analysis of *TaPHT1;9-4B* and *TaMYB4-7D*

Genomic DNA samples were isolated from 62 bread wheat lines (21 landraces and 41 modern cultivars), 11 tetraploid wheat accessions, and 28 diploid wheat relatives (*Ae. bicornis*, *Ae.*

longissima, *Ae. sharonensis*, *Ae. speltoides*, and *Ae. tauschii*) (Table S1). They were used for amplifying the promoter and coding sequences of *TaPHT1;9* or *TaMYB4* from polyploidy wheat lines using homoeolog specific primer sets (Table S2); these primers were also effective for obtaining the orthologous sequences of *TaPHT1;9* or *TaMYB4* in diploid *Aegilops* materials. The resultant PCR products were cloned into the pEASY-Blunt vector (CB101-02, TransGen, Beijing, China) and sequenced with at least three independent clones. The resulting sequences were aligned by Clustal Omega (https://www. ebi.ac.uk/Tools/msa/clustalo/), and SNPs were identified by DNASTAR (http://www.dnastar.com/). Nucleotide diversity (π) analysis was carried out using DnaSP 5.10 (http://www.ub.edu/dnasp/).

Haplotype analysis

Haplotype analysis was carried out for the promoter region of *TaPHT1;9-4B* based on 9 SNPs. The resultant 4 haplotypes (*Hap1* to *Hap4*) were assayed for promoter activities using β-glucuronidase (GUS) assay. Different promoter haplotypes were respectively inserted into the pCAMBIA1301 vector containing the GUS reporter gene by PCR mediated cloning with the primers listed in Table S2. The constructs were individually delivered into germinating wheat grains using particle bombardment, with the GUS staining as described before (Wang *et al.,* 2013; Yang *et al.,* 2016). GUS activities of 4 haplotype (*Hap1* to *Hap4*) promoters were determined according to a previously described method (Zheng *et al*., 2007). The extracts (100 μl) from the transformed wheat grains were added to 1 ml of assay buffer (50 mM phosphate buffer, pH 7.0, 10 mM EDTA, 0.1 % TritonX-100, 10 mM *β*-mercaptoethanol, and 1 mM MUG), and incubated at 37 °C. At different incubation time points (0, 5, 10, 20, 30, and 60 min), 200 μl samples were removed and placed in 800 μl stop buffer (200 μM Na_2CO_3). GUS activities were determined by using a Microplate Reader (Synergy HTX, BioTek), and calculated as micromoles 4-MU per min per mg protein. The actual GUS measurement values were directly used for comparing the promoter activities of the four haplotypes. Subsequently, two separate experiments were conducted to investigate potential associations of the four promoter haplotypes with *TaPHT1;9-4B* transcript level as well as the growth performance, biomass, and P accumulation of 16 commercial bread wheat cultivars. In the first experiment, two-week-old wheat seedlings of the 16 cultivars were grown hydroponically in low Pi (50 μM) Hoagland solution for 10 d, followed by examining the transcript level of *TaPHT1;9-4B* and total P content. The second experiment was conducted similarly, except that the plants were cultured in low Pi (50 μM) medium for 14 d to facilitate more reliable assessment of growth phenotypes, dry weights, P concentration, and P content.

Development of CAPS marker

The two G/C SNPs at the positions -799 and -796 relative to the start codon ATG of *TaPHT1;9-4B* coding sequence were employed to develop a specific CAPS marker (CAPS-799) for *Hap3* with the primers CAPS-799F/R (Table S2). The PCR amplicons resulted from using CAPS-799F/R were digested using the restriction endonuclease *Fnu*4HI, yielding a band pattern diagnostic for *Hap3*.

Chromosomal assignment of genes and subcellular localization of proteins

Chromosomal locations of *TaPHT1;9-4B* or *TaMYB4-7D* were investigated by PCR amplification of genomic DNA samples from CS and its nullisomic-tetrasomic lines (Aramrak *et al.,* 2015). Subgenome specific primer pairs for *TaPHT1;9* (*TaPHT1;9-4A*, *-4B*, and *-4D*) and *TaMYB4* (*TaMYB4-7A*, *-7B*, and *-7D*) homoeologs were designed based on polymorphisms in their genomic sequences (Table S2). To reveal the subcellular localization of TaPHT1;9-4B and TaMYB4-7D, their coding sequence without stop codon was each inserted upstream of that of GFP in a modified pCAMBIA1301 vector to create the fusion constructs pTaPHT1;9-4B-GFP and pTaMYB4-7D-GFP. These constructs were introduced into *N. benthamiana* leaf cells by *Agroinfiltration* (Gao et al., 2018). In these localization assays, we used AtRCI2B-RFP and H2B-mCherry fusion proteins as positive markers for the plasma membrane and nucleus, respectively, according to previous studies (Medina *et al*., 2007; Rosa *et al*., 2014). AtRCI2B is a small, highly hydrophobic protein that has been proved to reside in plant plasma membrane (Medina *et al*., 2007). GFP and RFP signals were examined using a confocal laser scanning microscope (FV10-ASW, Olympus, Tokyo, Japan).

qRT-PCR

Gene expression was evaluated by qRT-PCR with a Light Cycler 480 Real-Time PCR System (Roche Diagnostics Ltd., West Sussex, UK) using the SYBR Premix ExTaq (Perfect Real Time) Kit (Takara Biotechnology Co. Ltd., Dalian, China). Transcript levels were calculated using the $2^{-\Delta\Delta Ct}$ method (Schmittgen & Livak, 2008), with the amplification of *TaActin* as an internal control. In general, three biological replicates were analyzed per sample; the transcript level of the control was set as 1 to facilitate cross comparison of expression profiles among different treatments (or groups of plants).

References

Ahmed N, Maekawa M, Noda K. 2009. Anthocyanin accumulation and expression pattern of anthocyanin biosynthesis genes in developing wheat coleoptiles. *Biologia Plantarum* **53:**223–228.

Aramrak A, Kidwell KK, Steber C, Burke IC. 2015. Molecular and phylogenetic characterization of the homoeologous *EPSP Synthase* genes of allohexaploid wheat, *Triticum aestivum* (L.). *BMC Genomics* **16:** 844.

Bun-Ya M, Nishimura M, Harashima S, Oshima Y. 1991. The *PHO84* gene of *Saccharomyces cerevisiae* encodes an inorganic phosphate transporter. *Molecular and Cell Biology* **11:** 3229–3238.

Chen AQ, Hu J, Sun SB, Xu GH. 2007a. Conservation and divergence of both phosphate- and mycorrhiza-regulated physiological responses and expression patterns of phosphate transporters in solanaceous species. *New Phytologist* **173:** 817–831.

Chen R, Zhao X, Shao Z, Wei Z, Wang Y, Zhu L, Zhao J, Sun M, He R, He G. 2007b. Rice UDP-glucose pyrophosphorylase1 is essential for pollen callose deposition and its co-suppression results in a new type of thermo-sensitive genic male sterility. *The Plant Cell* **19:** 847–861.

Gao T, Li GZ, Wang CR, Dong J, Yuan SS, Wang YH, Kang GZ. 2018. Function of the ERFL1a transcription factor in wheat responses to water deficiency. *International Journal of Molecular Sciences* **19:** 1465.

Hellens RP, Allan AC, Friel EN, Templeton MD, Karunairetnam S, Laing WA. 2005. Transient plant expression vectors for functional genomics, quantification of promoter activity and RNA silencing. *Plant Methods* **1:** 13.

Hiei Y, Ohta S, Komari T, Kumashiro T. 1994. Efficient transformation of rice (*Oryza sativa* L.) mediated by *Agrobacterium* and sequence analysis of the boundaries of the T-DNA. *Plant Journal for Cell & Molecular Biology* **6:** 271–282.

Hu G, Koh J, Yoo MJ, Pathak D, Chen S, Wendel JF. 2014. Proteomics profiling of fiber development and domestication in upland cotton (*Gossypium hirsutum* L.). *Planta* **240:** 1237–1251.

Hu G, Koh J, Yoo MJ, Chen S, Wendel JF. 2015. Gene-expression novelty in allopolyploid cotton: a proteomic perspective. *Genetics* **200:** 91–104.

Kang G, Li G, Wang L, Wei L, Yang Y, Wang P, Yang Y, Wang Y, Feng W, Wang C, Guo T. 2015. Hg-responsive proteins identified in wheat seedlings using iTRAQ analysis and the role of ABA in Hg stress. *Journal of Proteome Research* **14:** 249–267.

Kim MJ, Kim JK, Shin JS, Suh MC. 2007. The SebHLH transcription factor mediates trans-activation of the SeFAD2 gene promoter through binding to E- and G-box elements. *Plant Molecular Biology* **64:** 453–466.

Lee WS, Rudd JJ, Kanyuka K. 2015. Virus induced gene silencing (VIGS) for functional analysis of wheat genes involved in *Zymoseptoria tritici* susceptibility and resistance. *Fungal Genetics and Biology* **79:** 84–88.

Li G, Wu Y, Liu G, Xiao X, Wang P, Gao T, Xu M, Han Q, Wang Y, Guo TC, Kang G. 2017. Large-scale proteomics combined with transgenic experiments demonstrates an important role of jasmonic acid in potassium deficiency response in wheat and rice. *Molecular & Cellular Proteomics* **16:** 1889–1905.

Li GZ, Wang ZQ, Yokosho K, Ding B, Fan W, Gong QQ, Li GX, Wu YR, Yang JL, Ma JF, Zheng SJ. 2018. Transcription factor WRKY22 promotes aluminum tolerance via activation of *OsFRDL4* expression and enhancement of citrate secretion in rice (*Oryza sativa*). *New Phytologist* **219:** 149–162.

Liu G, Wu Y, Xu M, Gao T, Wang P, Wang L. Guo T, Kang G. 2016. Virus-induced gene silencing identifies an important role of the TaRSR1 transcription factor in starch synthesis in bread wheat. *International Journal of Molecular Sciences* **17:**1557.

Liu H, Wang K, Jia Z, Gong Q, Lin Z, Du L, Pei X, Ye X. 2020. Efficient induction of haploid plants in wheat by editing of TaMTL using an optimized *Agrobacterium*-mediated CRISPR system. *Journal of Experimental Botany* **71:**1337–1349.

Mao C, Ding J, Zhang B, Xi D, Ming F. 2018. OsNAC2 positively affects salt-induced cell death and binds to the *OsAP*37 and *OsCOX11* promoters. *The Plant Journal* **94:** 454–468.

Ma HZ, Liu GQ, Li CW, Kang GZ, Guo TC. 2012. Identification of the *TaBTF3* gene in wheat (*Triticum aestivum L.*) and the effect of its silencing on wheat chloroplast, mitochondria and mesophyll cell development. *Biochemical and biophysical research communications* **426:** 608–614.

Ma X, Zhang Q, Zhu Q, Liu W, Chen Y, Qiu R, Wang B, Yang Z, Li H, Lin Y, et al. 2015. A robust CRISPR/Cas9 system for convenient, high-efficiency multiplex genome editing in monocot and dicot plants. *Molecular Plant* **8:**1274–1284.

Medina J, Ballesteros ML, Salinas J. 2007. Phylogenetic and functional analysis of *Arabidopsis RCI2* genes. *Journal of Experimental Botany* **58:** 4333–4346.

Powell JJ, Fitzgerald TL, Stiller J, Berkman PJ, Gardiner DM, Manners JM, Henry RJ, Kazan K. 2017. The defence-associated transcriptome of hexaploid wheat displays homoeolog expression and induction bias. *Plant Biotechnology Journal* **15:** 533–543.

Ramírez-González RH, Borrill P, Lang D, Harrington SA, Brinton J, Venturini L, Davey M, Jacobs J, van Ex F, Pasha A,et al. 2018. The transcriptional landscape of polyploid wheat. *Science* **361:**6403.

Rosa S, Ntoukakis V, Ohmido N, Pendle A, Abranches, R, Shaw P. 2014. Cell differentiation and development in *Arabidopsis* are associated with changes in histone dynamics at the single-cell level. *Plant Cell* **26:**4821–4833.

Sandberg A, Lindell G, Källström BN, Branca RM, Danielsson KG, Dahlberg M, Larson B, Forshed J, Lehtiö J. 2012. Tumor proteomics by multivariate analysis on individual pathway data for characterization of vulvar cancer phenotypes. *Molecular & Cellular Proteomics* **11:** M112.016998.

Schmittgen TD, Livak KJ. 2008. Analyzing real-time PCR data by the comparative C_T method. *Nature Protocols*, **3:**1101–1108.

Sun L, Song L, Zhang Y, Zheng Z, Liu D. 2015. Arabidopsis PHL2 and PHL1 act redundantly as the key components of the central regulatory system controlling transcriptional responses to phosphate starvation *Plant Physiology* **170:** 499–512.

Sun S, Gu M, Cao Y, Huang X, Zhang X, Ai P, Zhao J, Fan X, Xu G. 2012. A constitutive expressed phosphate transporter, OsPht1;1, modulates phosphate uptake and translocation in phosphate-replete rice. *Plant Physiology* **159:** 1571–1581.

Teng W, Zhao YY, Zhao XQ, He X, Ma WY, Deng Y, Chen XP, Tong YP. 2017. Genome-wide identification, characterization, and expression analysis of PHT1 phosphate transporters in wheat. *Frontiers in Plant Science* **8:** 543.

Ternent T, Csordas A, Oi D, Gómez-Baena G, Beynon RJ, Jones AR, Hermjakob HH, Vizcaino JA. 2014. How to submit MS proteomics data to proteomeXchange via the PRIDE database. *Proteomics* **14:** 2233–2241.

Torabi S, Wissuwa M, Heidari M, Naghavi MR, Gilany K, Hajirezaei MR, Omidi M, Yazdi-Samadi B, Ismail AM, Salekdeh GH. 2009. A comparative proteome approach to decipher the mechanism of rice adaptation to phosphorus deficiency. *Proteomics* **9:** 159–170.

Upadhyaya NM, Surin B, Ramm K, Gaudron J, Taylor W. 2000. Agrobacterium-mediated transformation of Australian rice cultivars Jarrah and Amaroo using modified promoters and selectable markers. *Australian Journal of Plant Physiology* **27:** 201–210.

Wang K, Liu HY, Du LP, Ye XG. 2017. Generation of marker-free transgenic hexaploid wheat via an *Agrobacterium*-mediated co-transformation strategy in commercial Chinese wheat varieties. Plant Biotechnology Journal **15:**1–13.

Wang K, Zhang X, Zhao Y, Chen F, Xia G. 2013. Structure, variation and expression analysis of glutenin gene promoters from *Triticum aestivum* cultivar Chinese Spring shows the distal region of promoter 1Bx7 is key regulatory sequence. *Gene* **527:** 484–490.

Wang R, Jing W, Xiao L, Jin Y, Shen L, Zhang W. 2015b. The rice high-affinity potassium transporter1;1 is involved in salt tolerance and regulated by an MYB-type transcription factor. *Plant Physiology* **168:** 1076–1090.

Wang ZQ, Li GZ, Gong QQ, Li GX, Zheng SJ. 2015a. *OsTCTP*, encoding a translationally controlled tumor protein, plays an important role in mercury tolerance in rice. *BMC Plant Biology* **15:**123.

Yang Q, Deng M, Zhang LL, Zhang XW, Wang LN, Chen H, Ma J, Qi PF, Jiang QT, Lan XJ, e*t al.* 2016. A super twin T-DNA vector that allows independent gene expression during *Agrobacterium*-mediated transformation. *Plasmid* **87–88:** 58–64.

Zauber H, Kirchner M, Selbach M. 2018. Picky: a simple online PRM and SRM method designer for targeted proteomics. *Nature Methods* **15:** 156–157.
